# Stochastic Hybrid Systems in Cellular Neuroscience

**DOI:** 10.1186/s13408-018-0067-7

**Published:** 2018-08-22

**Authors:** Paul C. Bressloff, James N. Maclaurin

**Affiliations:** Department of Mathematics, University of Utah, Salt Lake City, USA

## Abstract

We review recent work on the theory and applications of stochastic hybrid systems in cellular neuroscience. A stochastic hybrid system or piecewise deterministic Markov process involves the coupling between a piecewise deterministic differential equation and a time-homogeneous Markov chain on some discrete space. The latter typically represents some random switching process. We begin by summarizing the basic theory of stochastic hybrid systems, including various approximation schemes in the fast switching (weak noise) limit. In subsequent sections, we consider various applications of stochastic hybrid systems, including stochastic ion channels and membrane voltage fluctuations, stochastic gap junctions and diffusion in randomly switching environments, and intracellular transport in axons and dendrites. Finally, we describe recent work on phase reduction methods for stochastic hybrid limit cycle oscillators.

## Introduction

There are a growing number of problems in cell biology that involve the coupling between a piecewise deterministic differential equation and a time-homogeneous Markov chain on some discrete space *Γ*, resulting in a stochastic hybrid system, also known as a piecewise deterministic Markov process (PDMP) [37]. Typically, the phase space of the dynamical system is taken to be $\mathbb {R}^{d}$ for finite *d*. One important example at the single-cell level is the occurrence of membrane voltage fluctuations in neurons due to the stochastic opening and closing of ion channels [2, 25, 30, 32, 54, 64, 80, 109, 114, 117]. Here the discrete states of the ion channels evolve according to a continuous-time Markov process with voltage-dependent transition rates and, in-between discrete jumps in the ion channel states, the membrane voltage evolves according to a deterministic equation that depends on the current state of the ion channels. In the limit that the number of ion channels goes to infinity, we can apply the law of large numbers and recover classical Hodgkin–Huxley-type equations. However, finite-size effects can result in the noise-induced spontaneous firing of a neuron due to channel fluctuations. Another important example is a gene regulatory network, where the continuous variable is the concentration of a protein product, and the discrete variable represents the activation state of the gene [79, 83, 108, 110, 131]. Stochastic switching between active and inactive gene states can allow a gene regulatory network to switch between graded and binary responses, exhibit translational/transcriptional bursting, and support metastability (noise-induced switching between states that are stable in the deterministic limit). If random switching persists at the phenotypic level, then this can confer certain advantages to cell populations growing in a changing environment, as exemplified by bacterial persistence in response to antibiotics. A third example occurs within the context of motor-driven intracellular transport [23]. One often finds that motor-cargo complexes randomly switch between different velocity states such as anterograde versus retrograde motion, which can be modeled in terms of a special type of stochastic hybrid system known as a velocity jump process.

In many of the examples mentioned, we find that the transition rates between the discrete states $n\in\varGamma$ are much faster than the relaxation rates of the piecewise deterministic dynamics for $x\in \mathbb {R}^{d}$. Thus, there is a separation of time-scales between the discrete and continuous processes, so that if *t* is the characteristic time-scale of the relaxation dynamics, then *εt* is the characteristic time-scale of the Markov chain for some small positive parameter *ε*. Assuming that the Markov chain is ergodic, in the limit $\varepsilon \rightarrow0$, we obtain a deterministic dynamical system in which one averages the piecewise dynamics with respect to the corresponding unique stationary measure. This then raises the important problem of characterizing how the law of the underlying stochastic process approaches this deterministic limit in the case of weak noise, $0<\varepsilon \ll1$.

The notion of a stochastic hybrid system can also be extended to piecewise deterministic partial differential equations (PDEs), that is, infinite-dimensional dynamical systems. One example concerns molecular diffusion in cellular and subcellular domains with randomly switching exterior or interior boundaries [12, 17–19, 92]. The latter are generated by the random opening and closing of gates (ion channels or gap junctions) within the plasma membrane. In this case, we have a diffusion equation with boundary conditions that depend on the current discrete states of the gates; the particle concentration thus evolves piecewise, in between the opening or closing of a gate. One way to analyze these stochastic hybrid PDEs is to discretize space using finite-differences (method of lines) so that we have a standard PDMP on a finite-dimensional space. Diffusion in randomly switching environments also has applications to the branched network of tracheal tubes forming the passive respiration system in insects [18, 92] and volume neurotransmission [90].

This tutorial review develops the theory and applications of stochastic hybrid systems within the context of cellular neuroscience. A complementary review that mainly considers gene regulatory networks can be found elsewhere [14]. In Sect. 2, we summarize the basic theory of stochastic hybrid systems, In subsequent sections, we consider various applications of stochastic hybrid systems, including stochastic ion channels and membrane voltage fluctuations (Sect. 3), stochastic gap junctions and diffusion in randomly switching environments (Sect. 4), and intracellular transport in axons and dendrites (Sect. 5). Finally, in Sect. 6, we present recent work on phase reduction methods for stochastic hybrid limit cycle oscillators.

## Stochastic Hybrid Systems

In this section, we review the basic theory of stochastic hybrid systems. We start with the notion of a piecewise deterministic differential equation, which can be used to generate sample paths of the stochastic process. We then describe how the probability distribution of sample paths can be determined by solving a differential Chapman–Kolmogorov (CK) equation (Sect. 2.1). In many applications, including the stochastic ion channel models of Sect. 3, there is a separation of time-scales between a fast $O(1/\varepsilon )$ switching process and a slow $O(1)$ continuous dynamics. In the fast switching limit $\varepsilon \rightarrow0$, we obtain a deterministic dynamical system. In Sect. 2.2, we use an asymptotic expansion in *ε* to show how the CK equation can be approximated by the Fokker–Planck (FP) equation with an $O(\varepsilon )$ diffusion term (Sect. 2.2). Finally, in Sect. 2.3, we consider methods for analyzing escape problems in stochastic hybrid systems. We assume that the deterministic system is bistable so that, in the absence of noise, the long-time stable state of the system depends on the initial conditions. On the other hand, for finite switching rates, the resulting fluctuations can induce transitions between the metastable states. In the case of weak noise (fast switching $0 <\varepsilon \ll1$), transitions are rare events involving large fluctuations that are in the tails of the underlying probability density function. This means that estimates of mean first passage times (MFPTs) and other statistical quantities can develop exponentially large errors under the diffusion approximation. We describe a more accurate method for calculating MFPTs based on a WKB analysis.

We begin with the definition of a stochastic hybrid system and, in particular, a piecewise deterministic Markov process (PDMP) [37, 53, 84]. For illustration, consider a system whose states are described by a pair $(x,n) \in\varSigma\times\{0,\ldots,N_{0}-1\}$, where *x* is a continuous variable in a connected bounded domain $\varSigma\subset \mathbb {R}^{d}$ with regular boundary *∂Ω*, and *n* is a discrete stochastic variable taking values in the finite set $\varGamma\equiv\{ 0,\ldots,N_{0}-1\}$. (It is possible to have a set of discrete variables, although we can always relabel the internal states so that they are effectively indexed by a single integer. We can also consider generalizations of the continuous process, in which the ODE (2.1) is replaced by a stochastic differential equation (SDE) or even a partial differential equation (PDE). To allow for such possibilities, we will refer to all of these processes as examples of a stochastic hybrid system.) When the internal state is *n*, the system evolves according to the ordinary differential equation (ODE)

2.1 $$ \dot{x}=F_{n}(x), $$

where the vector field $F_{n}: \mathbb {R}\to \mathbb {R}$ is a continuous function, locally Lipschitz. That is, given a compact subset $\mathscr {K}$ of *Σ*, there exists a positive constant $K_{n}$ such that

2.2 $$ \bigl\vert F_{n}(x)-F_{n}(y) \bigr\vert \leq K_{n} |x-y|,\quad \forall x,y\in\varSigma. $$

We assume that the dynamics of *x* is confined to the domain *Σ* so that existence and uniqueness of a trajectory holds for each *n*. For fixed *x*, the discrete stochastic variable evolves according to a homogeneous continuous-time Markov chain with transition matrix $\mathbf{W}(x)$ and corresponding generator $\mathbf{A}(x)$, which are related according to

2.3 $$ A_{nm}(x)=W_{nm}(x)-\delta_{n,m} \sum _{k} W_{kn}(x). $$

The matrix $\mathbf{A}(x)$ is also taken to be Lipschitz. We make the further assumption that the chain is irreducible for all $x\in\varSigma$, that is, for fixed *x*, there is a nonzero probability of transitioning, possibly in more than one step, from any state to any other state of the Markov chain. This implies the existence of a unique invariant probability distribution on *Γ* for fixed $x\in\varSigma $, denoted by $\rho(x)$, such that

2.4 $$ \sum_{m\in\varGamma}A_{nm}(x) \rho_{m}(x)=0,\quad \forall n \in\varGamma. $$

**Fig. 1:**
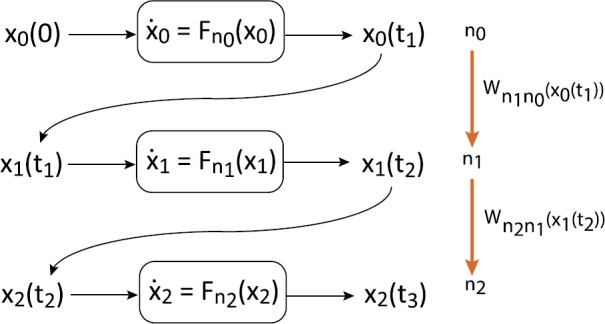
Schematic illustration of a piecewise deterministic Markov process

Let us decompose the transition matrix of the Markov chain as

$$W_{nm}(x)=P_{nm}(x)\lambda_{m}(x) $$

with $\sum_{n\neq m}P_{nm}(x)=1$ for all *x*. Hence $\lambda_{m}(x)$ determines the jump times from the state *m*, whereas $P_{nm}(x)$ determines the probability distribution that when it jumps, the new state is *n* for $n\neq m$. The hybrid evolution of the system with respect to $x(t)$ and $n(t)$ can then be described as follows; see Fig. 1. Suppose the system starts at time zero in the state $(x_{0}, n_{0})$. Call $x_{0}(t)$ the solution of (2.1) with $n=n_{0}$ such that $x_{0}(0)=x_{0}$. Let $t_{1}$ be the random variable (stopping time) such that

$$\mathbb {P}(t_{1} < t) =1- \exp \biggl( - \int_{0}^{t}\lambda_{n_{0}} \bigl(x_{0}\bigl(t'\bigr)\bigr)\,dt' \biggr). $$

Then in the random time interval $s\in[0, t_{1})$ the state of the system is $(x_{0}(s),n_{0})$. Now draw a value of $t_{1}$ from $\mathbb {P}(t_{1} < t)$, choose an internal state $n_{1} \in\varGamma$ with probability $P_{n_{1}n_{0}}(x_{0}(t_{1}))$, and call $x_{1}(t)$ the solution of the following Cauchy problem on $[t_{1},\infty)$:

$$\textstyle\begin{cases} \dot{x}_{1}(t) = F_{n_{1}}(x_{1}(t)), \quad t \geq t_{1}, \\ x_{1}(t_{1}) = x_{0}(t_{1}). \end{cases} $$

Iterating this procedure, we can construct a sequence of increasing jumping times $(t_{k})_{k \geq0}$ (setting $t_{0}=0$) and a corresponding sequence of internal states $(n_{k})_{k \geq0}$. The evolution $(x(t), n(t))$ is then defined as

2.5 $$ \bigl(x(t),n(t)\bigr)=\bigl(x_{k}(t),n_{k} \bigr) \quad \mbox{if } t_{k} \leq t < t_{k+1}. $$

Note that the path $x(t)$ is continuous and piecewise $C^{1}$. To have a well-defined dynamics on $[0,T]$, it is necessary that almost surely the system makes a finite number of jumps in the time interval $[0,T]$. This is guaranteed in our case. This formulation is the basis of a simulation algorithm for PDMPs [2, 150].

### Chapman–Kolmogorov Equation

Let $X(t)$ and $N(t)$ denote the stochastic continuous and discrete variables, respectively, at time *t*, $t>0$, given the initial conditions $X(0)=x_{0}$, $N(0)=n_{0}$. Introduce the probability density $p_{n}(x,t|x_{0},n_{0},0) $ with

$$\mathbb {P}\bigl\{ X(t)\in(x,x+dx), N(t) =n|x_{0},n_{0}\bigr\} =p_{n}(x,t|x_{0},n_{0},0) \,dx. $$

It follows that *p* evolves according to the forward differential Chapman–Kolmogorov (CK) equation [10, 61]

2.6 $$ \frac{\partial p_{n}}{\partial t}=-\nabla\cdot\bigl[F_{n}(x)p_{n}(x,t) \bigr]+\frac {1}{\varepsilon}\sum_{m\in\varGamma}A_{nm} p_{m}(x,t). $$

For notational convenience, we have dropped the explicit dependence on initial conditions. The first term on the right-hand side represents the probability flow associated with the piecewise deterministic dynamics for a given *n*, whereas the second term represents jumps in the discrete state *n*. Note that we have rescaled the matrix **A** by introducing the dimensionless parameter $\varepsilon>0$. This is motivated by the observation that one often finds a separation of time-scales between the relaxation time for the dynamics of the continuous variable *x* and the rate of switching between the different discrete states *n*. The fast switching limit then corresponds to the case $\varepsilon \rightarrow0$. Let us now define the averaged vector field $\overline {F}: \mathbb {R}^{d} \to \mathbb {R}^{d}$ by

2.7 $$ \overline{F}(x)=\sum_{n \in\varGamma} \rho_{n}(x) F_{n}(x). $$

Intuitively speaking, we would expect the stochastic hybrid system (2.1) to reduce to the deterministic dynamical system

2.8 $$ \textstyle\begin{cases} \dot{x}(t) = \overline{F}(x(t)), \\ x(0) = x_{0}, \end{cases} $$

in the fast switching limit $\varepsilon\rightarrow0$. That is, for sufficiently small *ε*, the Markov chain undergoes many jumps over a small time interval *Δt* during which $\varDelta x\approx0$, and thus the relative frequency of each discrete state *n* is approximately $p_{n}^{*}(x)$. This can be made precise in terms of a law of large numbers for stochastic hybrid systems [51, 84].

It remains to specify boundary conditions for the CK equation. For illustration, suppose that $d=1$ (one-dimensional continuous dynamics) with $\varSigma=[0,L]$ and assume that there exists an integer *m*, $1\leq m \leq N_{0}-1$, such that $F_{n}(0)=0$ for $0\leq n \leq m-1$ and $F_{n}(L)=0$ for $m\leq n\leq N_{0}-1$. No-flux boundary conditions at the ends $x=0,L$ take the form $J(0,t)=J(L,t)=0$ with

2.9 $$ J(x,t)=\sum_{n=0}^{N_{0}-1}F_{n}(x) p_{n}(x,t) . $$

It follows that $p_{n}(0,t)=0$ for $m\leq n\leq N_{0}-1$ and $p_{n}(L,t)=0$ for $0\leq n\leq m-1$. In the analysis of metastability (Sect. 2.3), it will be necessary to impose an absorbing boundary condition at some interior point $x_{*}$ of the domain *Σ*, that is,

$$p_{n}(x_{*},t)=0,\quad 0\leq n\leq m-1. $$

In contrast to the no-flux conditions, there are nonzero fluxes through $x_{*}$.

In general, it is difficult to obtain an analytical steady-state solution of (2.6), assuming that it exists, unless $d=1$ and $N_{0}=2$ [46, 79]. The one-dimensional CK equation takes the form

2.10 $$ \frac{\partial p_{n}}{\partial t}=-\frac{\partial}{\partial x} \bigl[F_{n}(x)p_{n}(x,t) \bigr]+\frac{1}{\varepsilon}\sum_{m\in\varGamma}A_{nm}(x) p_{m}(x,t). $$

In the two-state case ($N_{0}=2$),

$$ \mathbf{A}(x)=\left ( \begin{matrix} -\alpha(x) &\beta(x) \\ \alpha(x) &-\beta(x) \end{matrix} \right ) $$

for a pair of transition rates $\alpha(x)$, $\beta(x)$, so that the steady-state version of (2.10) reduces to the pair of equations

2.11 $$\begin{aligned} 0&=-\varepsilon \frac{\partial}{\partial x}\bigl(F_{0}(x)p_{0}(x) \bigr)+\beta (x)p_{1}(x)-\alpha(x)p_{0}(x), \end{aligned}$$

2.12 $$\begin{aligned} 0&=-\varepsilon \frac{\partial}{\partial x}\bigl(F_{1}(x)p_{1}(x)\bigr)- \beta (x)p_{1}(x)+\alpha(x)p_{0}(x). \end{aligned}$$

Adding the pair of equations yields

2.13 $$ \frac{\partial}{\partial x}\bigl(F_{0}(x)p_{0}(x)\bigr)+ \frac{\partial}{\partial x}\bigl(F_{1}(x)p_{1}(x)\bigr)=0, $$

that is,

$$F_{0}(x)p_{0}(x)+F_{1}(x)p_{1}(x)=c $$

for some constant *c*. The reflecting boundary conditions imply that $c=0$. Since $F_{n}(x)$ is nonzero for all $x\in\varSigma$, we can express $p_{1}(x)$ in terms of $p_{0}(x)$:

2.14 $$ p_{1}(x)=-\frac{F_{0}(x)p_{0}(x)}{F_{1}(x)}. $$

Substituting into equation (2.11) gives

2.15 $$ 0=\varepsilon \frac{\partial}{\partial x}\bigl(F_{0}(x)p_{0}(x)\bigr)+ \biggl(\frac {\beta(x)}{F_{1}(x)}+\frac{\alpha(x)}{F_{0}(x)} \biggr)F_{0}(x)p_{0}(x). $$

This yields the solutions

2.16 $$ p_{n}(x)=\frac{1}{Z|F_{n}(x)|}\exp \biggl(-\frac{1}{\varepsilon } \int _{x_{*}}^{x} \biggl(\frac{\beta(y)}{F_{1}(y)}+ \frac{\alpha (y)}{F_{0}(y)} \biggr)\,dy \biggr), $$

where $x_{*}\in\varSigma$ is arbitrary and assuming that the normalization factor *Z* exists.

### Quasi-Steady-State (QSS) Diffusion Approximation

For small but nonzero *ε*, we can use perturbation theory to derive lowest order corrections to the deterministic mean field equation, which leads to the Langevin equation with noise amplitude $O(\sqrt{\varepsilon})$. More specifically, perturbations of the mean-field equation (2.8) can be analyzed using a quasi-steady-state (QSS) diffusion or adiabatic approximation, in which the CK equation (2.6) is approximated by the Fokker–Planck (FP) equation for the total density $C(x,t)=\sum_{n} p_{n}(x,t)$. The QSS approximation was first developed from a probabilistic perspective by Papanicolaou [119]. It has subsequently been applied to a wide range of problems in biology, including models of intracellular transport in axons [57, 123] and dendrites [111–113] and bacterial chemotaxis [73, 74, 116]. There have also been more recent probabilistic treatments of the adiabatic limit, which have been applied to various stochastic neuron models [118]. Finally, note that it is also possible to obtain a diffusion limit by taking the number of discrete states $N_{0}$ to be large [30, 117].

The basic steps of the QSS reduction are as follows:

(a) Decompose the probability density as

$$ p_{n}(x,t)=C(x,t)\rho_{n}(x) +\varepsilon w_{n}(x,t), $$

where $\sum_{n} p_{n}(x,t) =C(x,t)$ is the marginal probability density for the continuous variables *x*, and $\sum_{n} w_{n}(x,t)=0$. Substituting into equation (2.6) yields

$$\begin{aligned} \begin{aligned} \rho_{n}(x)\frac{\partial C}{\partial t}+\varepsilon \frac{\partial w_{n} }{\partial t}&=-\nabla\cdot \bigl(F_{n}(x)\bigl[\rho_{n}(x)C+\varepsilon w_{n} \bigr] \bigr) \\ &\quad {}+\frac{1}{\varepsilon }\sum_{m \in\varGamma} A_{nm}(x) \bigl[\rho _{m}(x)C+\varepsilon w_{m} \bigr]. \end{aligned} \end{aligned}$$

Summing both sides with respect to *n* then gives

2.17 $$ \frac{\partial C}{\partial t}=-\nabla\cdot \bigl[\overline{F}(x)C \bigr]- \varepsilon \sum_{n\in\varGamma} \nabla\cdot \bigl[ F_{n}(x)w_{n} \bigr], $$

where $\overline{F}(x)$ is the mean vector field of equation (2.7).

(b) Using the equation for *C* and the fact that $\mathbf{A}(x)\rho(x) = 0$, we have

$$\begin{aligned} \varepsilon \frac{\partial w_{n}}{\partial t} =&\sum_{m \in\varGamma} A_{nm}(x) w_{m}- \nabla\cdot \bigl[F_{n}(x) \rho_{n}(x) C \bigr]+\rho _{n}(x)\nabla\cdot \bigl[ \overline{F}(x)C \bigr] \\ &{} -\varepsilon \biggl[\nabla\cdot \bigl(F_{n}(x)\omega_{n} \bigr)- \rho_{n}(x)\sum_{m\in\varGamma} \nabla\cdot \bigl[ F_{m}(x)w_{m} \bigr] \biggr]. \end{aligned}$$

(c) Introduce the asymptotic expansion

$$w_{n}\sim{w}_{n}^{(0)}+\varepsilon {w}_{n}^{(1)}+ \varepsilon ^{2} {w}_{n}^{(2)}+\cdots $$

and collect $O(1)$ terms:

2.18 $$ \sum_{m \in\varGamma} A_{nm}(x) w^{(0)}_{m} = \nabla\cdot \bigl[\rho _{n}(x) F_{n}(x)C(x,t) \bigr]- \rho_{n}(x) \nabla\cdot \bigl[\overline {F}(x)C \bigr]. $$

The Fredholm alternative theorem (see the end of Sect. 2.3) shows that this has a solution, which is unique on imposing the condition $\sum_{n} w^{(0)}_{n}(x,t)=0$:

2.19 $$ w^{(0)}_{m}(x)=\sum _{n \in\varGamma} A^{\dagger}_{mn}(x) \bigl( \nabla\cdot \bigl[\rho_{n}(x) F_{n}(x)C(x,t) \bigr]- \rho_{n}(x) \nabla \cdot \bigl[\overline{F}(x)C \bigr] \bigr), $$

where $\mathbf{A}^{\dagger}$ is the pseudoinverse of the generator **A**. We typically have to determine the pseudoinverse of **A** numerically.

(d) Combining equations (2.19) and (2.17) shows that *C* evolves according to the Itô Fokker–Planck (FP) equation

2.20 $$ \frac{\partial C}{\partial t}=- \nabla\cdot \bigl[\overline{F}(x) C \bigr]- \varepsilon \nabla\cdot\bigl[{\mathscr {V}}(x)C\bigr]+\varepsilon \sum _{i,j=1}^{d}\frac{\partial^{2} D_{ij}(x) C}{\partial x_{i}\, \partial x_{j}}, $$

where the $O(\varepsilon )$ correction to the drift, ${\mathscr {V}}(x)$, and the diffusion matrix ${D}(x)$ are given by

2.21a $$ {\mathscr {V}}=\sum_{n,m} \bigl\{ ( \rho_{n} F_{n})\nabla\cdot\bigl(F_{m} A^{\dagger}_{mn}\bigr)-\overline{F}\nabla\cdot \bigl(F_{m} A^{\dagger}_{mn}\rho _{n}\bigr) \bigr\} $$

and

$$ D_{ij}(x) = \sum_{m,n\in\varGamma}F_{m,i}(x)A^{\dagger}_{mn}(x) \rho _{n}(x)\bigl[\overline{F}_{j}(x)-F_{n,j}(x) \bigr]. $$

Since $\sum_{m}A_{mn}^{\dagger}=0$, we can rewrite the diffusion matrix as

2.21b $$ D_{ij}(x) = \sum_{m,n\in\varGamma} \bigl[F_{m,i}(x) -\overline {F}_{i}(x)\bigr]A^{\dagger}_{mn}(x) \rho_{n}(x)\bigl[\overline{F}_{j}(x)-F_{n,j}(x) \bigr]. $$

In the one-dimensional case, the CK equation (2.10) reduces to the one-dimensional Itô FP equation

2.22 $$ \frac{\partial C}{\partial t}=- \frac{\partial}{\partial x}\bigl(\bigl[ \overline{F}(x)+\varepsilon {\mathscr {V}}(x)\bigr] C\bigr)+\varepsilon \frac {\partial^{2}}{\partial x^{2}} \bigl(D(x)C \bigr) $$

with the diffusion coefficient $D(x)$ given by

2.23 $$ D(x)=\sum_{n\in\varGamma}Z_{n}(x)F_{n}(x), $$

where $Z_{n}(x)$ is the unique solution to

2.24 $$ \sum_{m\in\varGamma} A_{nm}(x)Z_{m}(x)= \bigl[\overline{F}(x)-F_{n}(x)\bigr]\rho _{n}(x), \qquad \sum_{m}Z_{m}(x)=0. $$

For $N_{0}>2$, we typically have to solve equation (2.24) numerically in order to find the pseudoinverse of **A**. However, in the special case of a two-state discrete process ($n=0,1$), we have the explicit solution

2.25 $$ D(x)=\frac{\beta(x)[F_{0}(x)-\overline{F}(x)]F_{0}(x) +\alpha (x)[F_{1}(x)-\overline{F}(x)]F_{1}(x)}{[\alpha(x)+\beta(x)]^{2}}. $$

At a fixed point $x_{*}$ of the deterministic equation $\dot {x}=\overline{F}(x)$, we have $\overline{F}(x_{*})=0$ and $\beta (x_{*})F_{0}(x_{*})=-\alpha(x_{*})F_{1}(x_{*})$. This gives the reduced expression

2.26 $$ D(x_{*})=\frac{|F_{0}(x_{*})F_{1}(x_{*})|}{\alpha(x_{*})+\beta(x_{*})}. $$

One subtle point is the nature of boundary conditions under the QSS reduction, since the FP equation is a second-order parabolic PDE, whereas the original CK equation is an $N_{0}$th-order hyperbolic PDE. It follows that, for $N_{0}>2$, there is a mismatch in the number of boundary conditions between the CK and FP equations. This implies that the QSS reduction may break down in a small neighborhood of the boundary, as reflected by the existence of boundary layers [152]. One way to eliminate the existence of boundary layers is to ensure that the boundary conditions of the CK equation are compatible with the QSS reduction.

### Metastability in Stochastic Hybrid Systems

Several examples of stochastic hybrid systems are known to exhibit multistability in the fast-switching limit $\varepsilon \rightarrow0$ [14]. That is, the deterministic equation (2.8) supports more than one stable equilibrium. In the absence of noise, the particular state of the system is determined by initial conditions. On the other hand, when noise is included by taking into account the stochastic switching, fluctuations can induce transitions between the metastable states. If the noise is weak (fast switching $0 <\varepsilon \ll1$), then transitions are rare events involving large fluctuations that are in the tails of the underlying probability density function. This means that estimates of mean transition times and other statistical quantities can be sensitive to any approximations, including the Gaussian approximation based on the QSS approximation of Sect. 2.3, and can sometimes lead to exponentially large errors.

The analysis of metastability has a long history [70], particularly within the context of SDEs with weak noise. The underlying idea is that the mean rate to transition from a metastable state in the weak noise limit can be identified with the principal eigenvalue of the generator of the underlying stochastic process, which is a second-order differential operator in the case of a Fokker–Planck equation. Calculating the eigenvalue typically involves obtaining a Wentzel–Kramers–Brillouin (WKB) approximation of a quasistationary solution and then using singular perturbation theory to match the solution to an absorbing boundary condition [69, 97, 99, 103, 130]. The latter is defined on the boundary that marks the region beyond which the system rapidly relaxes to another metastable state, becomes extinct, or escapes to infinity. In one-dimensional systems ($d=1$), this boundary is simply an unstable fixed point, whereas in higher-dimensions ($d>1$), it is generically a $(d-1)$-submanifold. In the weak noise limit, the most likely paths of escape through an absorbing boundary are rare events, occurring in the tails of the associated functional probability distribution. From a mathematical perspective, the rigorous analysis of the tails of a distribution is known as large deviation theory [39, 53, 55, 138], which provides a rigorous probabilistic framework for interpreting the WKB solution in terms of optimal fluctuational paths. The analysis of metastability in chemical master equations has been developed along analogous lines to SDEs, combining WKB methods and large deviation principles[43, 45, 49, 53, 69, 75, 85, 124] with path-integral or operator methods [40, 41, 121, 128, 143]. The study of metastability in stochastic hybrid systems is more recent, and much of the theory has been developed in a series of papers on stochastic ion channels [25, 109, 114, 115], gene networks [108, 110], and stochastic neural networks [24]. Again there is a strong connection between WKB methods, large deviation principles [15, 51, 84], and formal path-integral methods [11, 26], although the connection is now more subtle.

For illustration, we will focus on a one-dimensional stochastic hybrid system and develop the theory using WKB methods. First, suppose that the deterministic equation (2.8) is written as

**Fig. 2:**
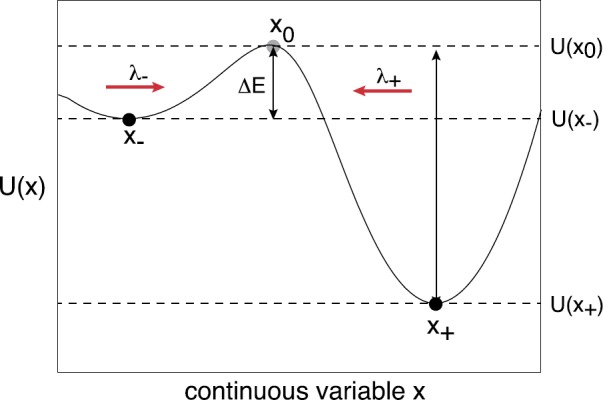
Sketch of a double-well potential of a bistable deterministic system in $\mathbb {R}$

2.27 $$ \dot{x}=\overline{F}(x)=-\frac{dU(x)}{dx} $$

with the potential $U(x)$ having two minima (stable equilibria) separated by a single maximum (unstable equilibrium), as illustrated in Fig. 2. To calculate the mean escape rate from the metastable state $x_{-}$, say, the CK equation (2.6) is supplemented by an absorbing boundary condition at $x=x_{0}$. The initial condition is taken to be $p_{n}(x,0|y,0)=\delta(x-y)\rho _{n}(y)$, where *y* is in a neighborhood of $x_{-}$, and $\rho_{n}(y)$ is the stationary distribution of the switching process. Let $T(y)$ denote the (stochastic) first passage time for which the system first reaches $x_{0}$, given that it started at *y*. The distribution of first passage times $f(t,y)$ is related to the survival probability that the system has not yet reached $x_{0}$:

2.28 $$ S(t,y)= \int_{\varSigma}\sum_{n\in\varGamma}p_{n}(x,t|y,0) \,dx. $$

That is, $\operatorname{Prob}\{t>T| X(0)=y\} =S(y,t)$, and the first passage time density $f(y,t)=-\partial S/\partial t$. Substituting for $\partial p_{n}/\partial t$ using the CK equation (2.10) shows that

2.29 $$ f(y,t) = \int_{ \varSigma} \biggl[\sum_{n \in\varGamma} \frac{\partial [F_{n}(x) p_{n}(x,t|y,0)]}{\partial x} \biggr]\,dx= \sum_{n \in\varGamma }p_{n}(x_{0},t|y,0) F_{n}(x_{0}) $$

with $\varGamma=\{0,1\}$ for the two-state model. We have used $\sum_{n \in\varGamma}{A}_{nm}(x)=0$ and the asymptotic limit $F_{n}(x)p_{n}(x,t|y,0)\rightarrow0$ as $x\rightarrow\pm\infty$. The mean first passage time (MFPT) $\tau(y)$ is then given by

$$ \tau(y)= \bigl\langle T(y)\bigr\rangle \equiv \int_{0}^{\infty}f(y,t)t\,dt = \int _{0}^{\infty} S(y,t)\,dt. $$

It turns out that for small *ε*, the MFPT has an *Arrhenius-like* form analogous to SDEs [69]:

$$ \tau(x_{-})=\frac{4\pi\varGamma(x_{0},x_{-})}{\sqrt{|\varPhi''(x_{0})|\varPhi ''(x_{-})}}\mathrm{e}^{[\varPhi(x_{0})-\varPhi(x_{-})]/\varepsilon }, $$

where $\varPhi(x)$ is known as the *quasipotential* or stochastic potential, and *Γ* is a prefactor. One important observation is that the escape time is exponentially sensitive to the precise form of *Φ*. If we were first to carry out the QSS reduction of Sect. 2.3 and then use a standard analysis of the one-dimensional FP equation in order to estimate the MFPT [61], then we would find that $\varGamma=1$ and, to $O(1)$,

2.30 $$ \varPhi_{\mathrm{QSS}}(x)= - \int^{x}\frac{\overline{F}(x')}{D(x')}\,dx' $$

with $D(x)$ given by equation (2.25). In particular, if $D(x)$ is independent of *x*, then $\varPhi(x)=U(x)/D$ with $U(x)$ the deterministic potential. The escape time then depends on the barrier height *ΔE* shown in Fig. 2. As we have already commented, the Gaussian approximation may not accurately capture the statistics of rare events that dominate noise-induced escape. This is reflected by the observation that $\varPhi_{\mathrm{QSS}}(x)$ can differ significantly from the true quasipotential. A much better estimate can be obtained using WKB.

To apply the WKB method, we can exploit the fact that, in the weak noise limit ($\varepsilon \ll1$), the flux through the absorbing boundary is exponentially small. This has major implications for the spectral decomposition of the solution to the CK equation with an absorbing boundary at $x=x_{0}$. More specifically, consider the eigenfunction expansion

2.31 $$ p_{n}(x,t)=\sum_{r}C_{r} \mathrm{e}^{-\lambda_{\varepsilon }^{(r)}t}{\phi }^{(r)}_{\varepsilon }(x,n), $$

where $(-\lambda_{\varepsilon }^{(r)},\phi_{\varepsilon }^{(r)}(x))$ is an eigenpair of the matrix-valued linear operator

$$\mathbb {L}_{\varepsilon }= -\frac{\partial}{\partial x}\operatorname {diag}\bigl(F_{n}(x)\bigr)+ \frac {1}{\varepsilon }\mathbf{A}(x) $$

appearing on the right-hand side of (2.6), that is,

2.32 $$ \mathbb {L}_{\varepsilon }{\phi}^{(r)}_{\varepsilon }=-\lambda_{\varepsilon }^{(r)}{ \phi}^{(r)}_{\varepsilon } $$

together with the absorbing boundary conditions ${\phi}^{(r)}_{\varepsilon }(x_{0},n)=0 $ for all *n*. We also assume that the eigenvalues $\lambda_{\varepsilon }^{(r)}$ all have positive definite real parts and the smallest eigenvalue $\lambda_{\varepsilon}^{(0)}$ is real and simple, so that we can introduce the ordering $0<\lambda _{\varepsilon }^{(0)}<\operatorname{Re}[\lambda_{\varepsilon }^{(1)}]\leq\operatorname {Re}[\lambda_{\varepsilon }^{(2)}]\leq\cdots$. The exponentially slow rate of escape through $x_{0}$ in the weak-noise limit means that $\lambda_{\varepsilon }^{(0)}$ is exponentially small, $\lambda _{\varepsilon }^{(0)}\sim\mathrm{e}^{-C/\varepsilon }$, whereas $\operatorname {Re}[\lambda_{\varepsilon }^{(r)}]$ is only weakly dependent on *ε* for $r\geq1$. Under these assumptions, we have the *quasistationary* approximation for large *t*:

2.33 $$ p_{n}(x,t)\sim C_{0} \mathrm{e}^{-\lambda_{\varepsilon }^{(0)} t}\phi _{\varepsilon }^{(0)}(x,n) . $$

Substituting such an approximation into equation (2.29) and suppressing the initial conditions give

2.34 $$ f(t)\sim C_{0} \mathrm{e}^{-\lambda_{\varepsilon }^{(0)} t}\sum _{n\in \varGamma} { F_{n}}(x_{0}) { \phi}_{\varepsilon }^{(0)}(x_{0},n), $$

and thus

2.35 $$ \lambda_{\varepsilon }^{(0)}= \frac{\sum_{n\in\varGamma}F_{n}(x_{0}){ \phi }_{\varepsilon }^{(0)}(x_{0},n)}{ \sum_{n}\int_{\varSigma}\phi ^{(0)}_{\varepsilon }(x,n)\,dx}. $$

Since $\lambda_{\varepsilon }^{(0)}$ is exponentially small, we can take the quasistationary solution ${ \phi}_{\varepsilon }^{(0)}$ to satisfy the time-independent CK equation. We then seek a WKB approximation of the quasistationary solution by making the ansatz

2.36 $$ \phi_{\varepsilon}^{(0)}(x,n)\sim{Z}_{n}(x) \exp \biggl(-\frac{\varPhi (x)}{\varepsilon} \biggr), $$

where $\varPhi(x)$ is the WKB quasipotential. Substituting into the time-independent version of equation (2.10) yields

2.37 $$ \sum_{m } \bigl(A_{nm}(x)+ \varPhi'(x)\delta_{n,m}F_{m}(x) \bigr)Z_{m}(x) =\varepsilon\frac{dF_{n}(x)Z_{n}(x)}{d x}, $$

where $\varPhi'=d\varPhi/dx$. Introducing the asymptotic expansions $\varPhi\sim\varPhi_{0}+\varepsilon \varPhi_{1}$ and $Z\sim Z^{(0)}+\varepsilon Z^{(1)}$, the leading order equation is

2.38 $$ \sum_{m \in\varGamma} A_{nm}(x)Z^{(0)}_{m}(x)+ \varPhi_{0}'(x)F_{n}(x)Z^{(0)}_{n}(x)=0. $$

Positivity of the quasistationary density $\phi_{\varepsilon}^{(0)}$ requires positivity of the corresponding solution $\mathbf{Z}^{(0)}$. One positive solution is the trivial solution $\mathbf{Z}^{(0)}(x)=\rho(x)$ for all $x\in\varSigma$, where *ρ* is the unique right eigenvector of **A**, for which $\varPhi_{0}'=0$. Establishing the existence of a nontrivial positive solution requires more work and is related to the fact that the connection of the WKB solution to optimal fluctuational paths and large deviation principles is less direct in the case of stochastic hybrid systems.

It turns out that we have to consider the eigenvalue problem [11, 15, 25, 51, 84]

2.39 $$ \sum_{m\in\varGamma} \bigl[A_{nm}(x) + q \delta_{n,m}F_{m}(x) \bigr]R_{m}(x, q) =\varLambda(x, q)R_{n}(x, q). $$

Assuming that $\mathbf{A}(x)$ is irreducible for all *x*, we can use the Perron–Frobenius theorem (see the end of Sect. 2.3) to show that, for fixed $(x,q)$, there exists a unique eigenvalue $\varLambda_{0}(x, q)$ with a positive eigenvector $R_{n}^{(0)}(x, q)$. The optimal fluctuational paths are obtained by identifying the Perron eigenvalue $\varLambda_{0}(x, q)$ as a Hamiltonian and finding zero energy solutions to Hamilton’s equations

2.40 $$ \dot{x} = \frac{\partial{H}}{\partial q}, \qquad \dot{q} = -\frac {\partial{H}}{\partial x},\qquad {H}(x, q) = \varLambda_{0}(x, q). $$

This can be established using large deviation theory or path-integrals. In the latter case, we can show that a path-integral representation of the density $p(x,\tau)$ is

$$ p(x,\tau)= \int _{x(0)=x_{0}}^{x(\tau)=x} \exp \biggl(-\frac{1}{\varepsilon }{ \int_{0}^{\tau}\bigl[q\dot{x}-\varLambda _{0}(x,q)\bigr]\,dt} \biggr){\mathscr {D}}[q,x] $$

for some appropriate measure ${\mathscr {D}}[q,x]$. Applying steepest descents to the path integral then yields a variational principle in which optimal paths minimize the action

$$ S[x,q]= \int_{0}^{\tau} \bigl[q\dot{x}-\varLambda_{0}(x,q) \bigr]\,dt . $$

Comparison of equation (2.38) with equation (2.39) then shows that there exists a nontrivial positive solution of equation (2.38) given by $Z_{n}^{(0)}(x)=R_{n}^{(0)}(x, q)$ with $q=\varPhi _{0}'(x)$ and $\varPhi_{0}$ satisfies the corresponding Hamilton–Jacobi equation

2.41 $$ \varLambda_{0}\bigl(x,\varPhi_{0}'(x)\bigr)=0. $$

Note that since $\varPhi'_{0}(x)$ vanishes at $x=x_{0}$, it follows that $\mathbf{Z}^{(0)}(x_{0})=\rho(x_{0}) $, and similarly for the other fixed points. Deterministic mean field equations and optimal paths of escape from a metastable state both correspond to zero energy solutions. Along zero-energy paths,

$$ S[x,q]\equiv \int_{-\infty}^{\tau} \bigl[q\dot{x}-\varLambda _{0}(x,q) \bigr]\,dt= \int_{-{\infty}}^{\tau} \varPhi_{0}'(x) \dot{x}\,dt = \int_{x_{s}}^{x} \varPhi'_{0}(x) \,dx. $$

#### Calculation of Principal Eigenvalue

To calculate the principal eigenvalue, it is necessary to determine the first-order correction $\varPhi_{1}$ to the quasipotential of the WKB solution (2.36). Proceeding to the next order in the asymptotic expansion of equation (2.37), we have

2.42 $$\begin{aligned} &\sum_{m} \bigl(A_{nm}(x)+ \varPhi_{0}'(x)\delta_{n,m}F_{n}(x) \bigr)Z^{(1)}_{m}(x) \\ &\quad =\frac{dF_{n}(x)Z_{n}^{(0)}(x)}{d x}-\varPhi _{1}'(x)F_{n}(x))Z_{n}^{(0)}(x). \end{aligned}$$

For fixed *x* and WKB potential $\varPhi_{0}$, the matrix operator

$$\bar{A}_{nm}(x)=A_{nm}(x)+\varPhi_{0}'(x) \delta_{n,m}F_{m}(x) $$

on the left-hand side of this equation has a one-dimensional null space spanned by the positive WKB solution $\mathbf{Z}^{(0)}(x)$. The Fredholm alternative theorem (see Sect. 2.2) then implies that the right-hand side of (2.42) is orthogonal to the left null vector *S* of *Ā*. That is, we have the solvability condition

$$ \sum_{n \in\varGamma}S_{n}(x) \biggl[ \frac{dF_{n}(x)Z_{n}^{(0)}(x)}{d x}-\varPhi _{1}'(x)F_{n}(x)Z_{n}^{(0)}(x) \biggr]=0 $$

with *S* satisfying

2.43 $$ \sum_{n \in\varGamma}S_{n}(x) \bigl(A_{nm}(x)+\varPhi_{0}'(x)\delta _{n,m}F_{m}(x) \bigr)=0. $$

Given $\mathbf{Z}^{(0)},\mathbf{S}$, and $\varPhi_{0}$, the solvability condition yields the following equation for $\varPhi_{1}$:

2.44 $$ \varPhi_{1}'(x)=\frac{\sum_{n \in\varGamma }S_{n}(x)[F_{n}(x)Z_{n}^{(0)}(x)]'}{\sum_{n \in\varGamma}S_{n}(x)F_{n}(x)Z_{n}^{(0)}(x)}. $$

Combining the various results and defining

2.45 $$ k(x)=\exp \bigl(-\varPhi_{1}(x) \bigr) $$

give to leading order in *ε*,

2.46 $$ \phi_{\varepsilon}^{(0)}(x,n)\sim{ \mathscr {N}}k(x)\exp \biggl(-\frac {\varPhi_{0}(x)}{\varepsilon} \biggr)Z_{n}^{(0)}(x), $$

where we choose $\sum_{n} Z_{n}^{(0)}(x)=1$ for all *x*, and ${\mathscr {N}}$ is the normalization factor,

$$ {\mathscr {N}}= \biggl[ \int_{\varSigma}k(x)\exp \biggl(-\frac{\varPhi _{0}(x)}{\varepsilon} \biggr) \biggr]^{-1}. $$

The latter can be approximated using Laplace’s method to give

2.47 $$ {\mathscr {N}}\sim\frac{1}{k(x_{-})}\sqrt{\frac{|\varPhi_{0}''(x_{-})|}{2\pi \varepsilon}}\exp \biggl( \frac{\varPhi_{0}(x_{-})}{\varepsilon} \biggr). $$

The final step is to use singular perturbation theory to match the outer quasistationary solution to the absorbing boundary condition at $x_{0}$. The analysis is quite involved [80, 108], so here we simply quote the result for the 1D model:

2.48 $$ \lambda_{\varepsilon }^{(0)} \sim\frac{1}{\pi}\frac{k(x_{0})D(x_{0})}{k(x_{-})} \sqrt{\varPhi _{0}''(x_{-}) \bigl\vert \varPhi_{0}''(x_{0}) \bigr\vert }\exp \biggl\{ -\frac{\varPhi_{0}(x_{0})-\varPhi _{0}(x_{-})}{\varepsilon} \biggr\} $$

with $D(x)$ the effective diffusion coefficient (2.23) obtained using a QSS reduction.

#### Two-State Model

We now illustrate the above theory for the simple two-state model of equation (2.10). The specific version of the linear equation (2.39) can be written as the two-dimensional system

2.49 $$ \left ( \begin{matrix} -\alpha(x)+qF_{0}(x)& \beta(x)\\ \alpha(x) & -\beta (x)+qF_{1}(x) \end{matrix} \right )\left ( \begin{matrix} R_{0} \\ R_{1} \end{matrix} \right )=\varLambda \left ( \begin{matrix} R_{0} \\ R_{1} \end{matrix} \right ). $$

The corresponding characteristic equation is

$$\begin{aligned} 0&=\varLambda^{2}+\varLambda\bigl[\alpha(x)+\beta(x)-q\bigl(F_{0}(x)+F_{1}(x) \bigr)\bigr] \\ &\quad {} +\bigl(qF_{1}(x)-\beta(x)\bigr) \bigl(qF_{0}(x)- \alpha(x)\bigr)-\beta(x)\alpha(x). \end{aligned}$$

It follows that the Perron eigenvalue is given by

2.50 $$ \varLambda_{0}(x,q)=\frac{1}{2} \bigl[ \varSigma(x,q)+\sqrt{ \varSigma(x,q)^{2}- 4h(x,q)} \bigr], $$

where

$$\varSigma(x,q)=q\bigl(F_{0}(x)+F_{1}(x)\bigr)-\bigl[\alpha(x)+ \beta(x)\bigr], $$

and

$$h(x,q)=q^{2}F_{1}(x)F_{0}(x)-q\bigl[ \beta(x)F_{0}(x)+\alpha(x)F_{1}(x)\bigr]. $$

A little algebra shows that

$$\begin{aligned} {\mathscr {D}}(x,q)&\equiv\varSigma(x,q)^{2}- 4h(x,q) \\ &=\bigl[q(F_{0}-F_{1})-\bigl(\alpha(x)-\beta(x)\bigr) \bigr]^{2}+\alpha(x)\beta(x)>0, \end{aligned}$$

so that, as expected, $\varLambda_{0}$ is real. The quasipotential $\varPhi _{0}(x)$ satisfies the HJ equation $\varLambda_{0}(x,q)=0$ with $q=\varPhi _{0}'(x)$, which reduces to the conditions

2.51 $$ h\bigl(x,\varPhi_{0}'(x)\bigr)=0,\qquad \varSigma\bigl(x, \varPhi_{0}'(x)\bigr)< 0. $$

This has two solutions: the classical deterministic solution $q=0$ with $\varPhi_{0}'(x)=0$ and a nontrivial solution whose quasipotential satisfies

2.52 $$ \varPhi_{0}'(x)=\frac{\beta(x)}{F_{1}(x)}+ \frac{\alpha(x)}{F_{0}(x)}. $$

(Note that $F_{n}(x)$ does not vanish anywhere and $F_{0}(x)F_{1}(x)<0$.) The quasipotential can be determined by numerically integrating with respect to *x*. The resulting quasipotential differs significantly from the one obtained by carrying out a QSS diffusion approximation of the stochastic hybrid system along the lines outlined in Sect. 2.2.

For this simple model, it is also straightforward to determine the various prefactors in equation (2.48). For example, the normalized positive eigenvector $\mathbf{Z}^{(0)}$ has the components

$$Z_{0}^{(0)}=\frac{F_{1}(x)}{F_{1}(x)-F_{0}(x)}, \qquad Z_{1}^{(0)}= \frac {-F_{0}(x)}{F_{1}(x)-F_{0}(x)} . $$

Since $F_{0}(x)<0$ and $F_{1}(x)>0$ for $x\in\varSigma$, it follows from equation (2.52) that $Z_{0}^{(0)}$ is positive. The components of the adjoint eigenvector **S** satisfy

$$\frac{S_{1}}{S_{0}}=\frac{-\alpha+\varPhi_{0}'(x)F_{0}(x)}{\alpha}=\frac {-\beta+\varPhi_{0}'(x)F_{1}(x)}{\beta}. $$

It then follows from equation (2.44) that the first correction to the quasipotential satisfies

2.53 $$ \varPhi_{1}'(x)=\frac{1}{F_{0}(x)F_{1}(x)}\frac{d}{dx} \bigl(F_{0}(x)F_{1}(x)\bigr). $$

Hence

2.54 $$ k(x)\equiv\mathrm{e}^{-\varPhi_{1}(x)}=\frac{1}{|F_{0}(x)|F_{1}(x)}. $$

Finally, $D(x_{0})$ is given by equation (2.26).

#### Fredholm Alternative Theorem

Consider an *M*-dimensional linear inhomogeneous equation $\mathbf{A}\mathbf{z}=\mathbf{b}$ with $\mathbf{z},\mathbf{b}\in{\mathbb {R}}^{M}$. Suppose that the $M\times M$ matrix **A** has a nontrivial null-space and let **u** be a null vector of the adjoint matrix $\mathbf{A}^{\dagger}$, that is, $\mathbf{A}^{\dagger}\mathbf{u}=0$. The Fredholm alternative theorem for finite-dimensional vector spaces states that the inhomogeneous equation has a (nonunique) solution for **z** if and only if $\mathbf{u}\cdot \mathbf{b}=0$ for all null vectors **u**. Let us apply this theorem to equation (2.18) for fixed *x*, *t*. The one-dimensional null-space is spanned by the vector with components $u_{n}=1$, since $\sum_{n}u_{n}A_{nm}=\sum_{n}A^{\dagger}_{mn}u_{n}=0$. Hence equation (2.18) has a solution, provided that

$$ 0=\sum_{n} \biggl[ \frac{\partial[F_{n}(x)p_{n}^{*}(x)C(x,t)]}{\partial x}-p_{n}^{*}(x) \frac{\partial\overline{F}(x)C}{\partial x} \biggr]. $$

This immediately follows since $\sum_{n}p_{n}(x)=1$ and $\sum_{n}p_{n}^{*}(x)F_{n}(x)= \overline{F}(x)$ for all *x*.

#### Perron–Frobenius Theorem

If **T** is an irreducible positive finite matrix, then

1. there is a simple eigenvalue $\lambda_{0}$ of **T** that is real and positive, with positive left and right eigenvectors;
2. the remaining eigenvalues *λ* satisfy $|\lambda|<\lambda_{0}$.

If $T_{nm}=W_{nm}/\sum_{k}W_{km}$, then $\lambda_{0}=1$, where **W** is an irreducible transition matrix, then the left positive eigenvector is $\psi=(1,\ldots,1)$, and the right positive eigenvector is the stationary distribution *ρ*. In the case of the matrix operator $\mathbf{L}(x)$ with components $L_{nm}(x):=A_{nm}(x)+qF_{n}(x)\delta _{n,m}$, which appears in the eigenvalue equation (2.39), it is clear that not all components of the matrix are positive for a given $x\in\varSigma$. However, taking $\zeta>\sup_{x\in\varSigma}\|\mathbf{L}(x)\|_{\infty}$, the matrix $\mathbf{L}(x)+\zeta\mathbf{I}$ satisfies the conditions of the Perron–Frobenius theorem for all $x\in\varSigma$.

## Stochastic Ion Channels and Membrane Voltage Fluctuations

**Fig. 3:**
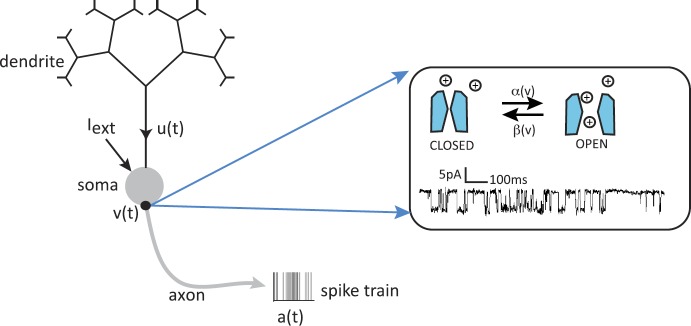
Opening and closing of ion channels underlying initiation and propagation of an action potential

The generation and propagation of a neuronal action potential arises from nonlinearities associated with active membrane conductances. Ions can diffuse in and out of the cell through ion specific channels embedded in the cell membrane; see Fig. 3. Ion pumps within the cell membrane maintain concentration gradients such that there is a higher concentration of Na^+^ and Ca^2+^ outside the cell and a higher concentration of K^+^ inside the cell. The membrane current through a specific channel varies approximately linearly with changes in the voltage *v* relative to some equilibrium or reversal potential, which is the potential at which there is a balance between the opposing effects of diffusion and electrical forces. (We will focus on a space-clamped model of a neuron whose cell body is taken to be an isopotential.) Summing over all channel types, the total membrane current (flow of positive ions) leaving the cell through the cell membrane is

3.1 $$ I_{\mathrm{con}} =\sum_{s} g_{s}(v-V_{s}), $$

where $g_{s}$ is the conductance due to channels of type *s*, and $V_{s}$ is the corresponding reversal potential.

Recordings of the current flowing through single channels indicate that channels fluctuate rapidly between open and closed states in a stochastic fashion. Nevertheless, most models of a neuron use deterministic descriptions of conductance changes, under the assumption that there are a large number of approximately independent channels of each type. It then follows from the law of large numbers that the fraction of channels open at any given time is approximately equal to the probability that any one channel is in an open state. The conductance $g_{s}$ for ion channels of type *s* is thus taken to be the product $g_{s}=\bar{g}_{s} P_{s}$ where $\bar{g}_{s}$ is equal to the density of channels in the membrane multiplied by the conductance of a single channel, and $P_{s}$ is the fraction of open channels. The voltage-dependence of the probabilities $P_{s}$ in the case of a delayed-rectifier K^+^ current and a fast Na^+^ current were originally obtained by Hodgkin and Huxley [76] as part of their Nobel prize winning work on the generation of action potentials in the squid giant axon. The delayed-rectifier K^+^ current is responsible for terminating an action potential by repolarizing a neuron. We find that opening of the K^+^ channel requires structural changes in four identical and independent subunits so that $P_{\mathrm{K}} = n^{4}$ where *n* is the probability that any one gate subunit has opened. In the case of the fast Na^+^ current, which is responsible for the rapid depolarization of a cell leading to action potential generation, the probability of an open channel takes the form $P_{\mathrm{Na}}=m^{3} h$ where $m^{3}$ is the probability that an activating gate is open and *h* is the probability that an inactivating gate is open. Depolarization causes *m* to increase and *h* to decrease, whereas hyperpolarization has the opposite effect.

The dynamics of the gating variables *m*, *n*, *h* are usually formulated in terms of a simple kinetic scheme that describes voltage-dependent transitions of each gating subunit between open and closed states. More specifically, for each $Y \in\{m,n,h \}$,

3.2 $$ \frac{dY}{dt}=\alpha_{Y}(v) (1-Y)-\beta_{Y}(v) Y, $$

where $\alpha_{Y}(v)$ is the rate of the transition $\mathit{closed} \rightarrow \mathit{open}$, and $\beta_{Y}(v)$ is the rate of the reverse transition $\mathit{open} \rightarrow \mathit{closed}$. From basic thermodynamic arguments, the opening and closing rates are expected to be exponential functions of the voltage *v*:

$$\alpha_{Y}(v)=A_{Y}\mathrm{e}^{-B_{Y}v},\qquad \beta_{Y}(v)=A'_{Y}\mathrm{e}^{-B'_{Y}v}. $$

Hodgkin and Huxley originally fitted exponential-like functions to the experimental data obtained from the squid axon. The corresponding conductance-based model (in the absence of synaptic inputs) can then be written in the form

3.3 $$ C\frac{dv}{dt} = f(v,m,n,h) + I_{\mathrm{ext}} $$

with

3.4 $$ f(v,m,n,h)=-\bar{g}_{\mathrm{Na}}m^{3}h(v-V_{\mathrm{Na}})- \bar{g}_{\mathrm{K}}n^{4}(v-V_{\mathrm{K}})-\bar{g}_{\mathrm{L}}(v-V_{\mathrm{L}}). $$

Here $I_{\mathrm{L}}=g_{\mathrm{L}}(v - V_{\mathrm{L}})$ is called a leak current, which represents the passive flow of ions through nongated channels.

### Morris–Lecar Model

It is often convenient to consider a simplified planar model of a neuron, which tracks the membrane voltage *v*, and a recovery variable *w* that represents the fraction of open potassium channels. The advantage of a two-dimensional model is that we can use phase-plane analysis to develop a geometric picture of neuronal spiking. One well-known example is the Morris–Lecar (ML) model [100]. Although this model was originally developed to model Ca^2+^ spikes in molluscs, it has been widely used to study neural excitability for Na^+^ spikes [48], since it exhibits many of the same bifurcation scenarios as more complex models. The ML model has also been used to investigate subthreshold membrane potential oscillations (STOs) due to persistent Na^+^ currents [27, 145]. Another advantage of the ML model is that it is straightforward to incorporate intrinsic channel noise [80, 109, 114, 132]. To capture the fluctuations in membrane potential from stochastic switching in voltage-gated ion channels, we will consider a stochastic version of the ML model that includes both discrete jump processes (to represent the opening and closing of Ca^2+^ or Na^+^ ion channels) and a two-dimensional continuous-time piecewise process (to represent the membrane potential and recovery variable *w*). We thus have an explicit example of a two-dimensional PDMP. (We can also consider fluctuations in the opening and closing of the K^+^ ion channels, in which *w* is replaced by an additional discrete stochastic variable, representing the fraction of open K^+^ channels [114, 132]. This would yield a one-dimensional PDMP for the voltage alone.)

#### Deterministic Model

First, consider a deterministic version of the ML model [100] consisting of a fast inward calcium current (Ca^2+^), a slow outward potassium current (K^+^), a leak current (L), and an applied current ($I_{\mathrm{app}}$). (In [80, 114] the inward current is interpreted as a Na^+^ current, but the same parameter values as the original ML model are used.) For simplicity, each ion channel is treated as a two-state system that switches between an open and a closed state—the more detailed subunit structure of ion channels is neglected [64]. The membrane voltage *v* evolves as

3.5 $$ \begin{aligned} C_{m}\frac{dv}{dt}&=a_{\infty}(v)f_{\mathrm{Ca}}(v)+wf_{K}(v)+f_{\mathrm{L}}(v)+I_{\mathrm{app}}, \\ \frac{dw}{dt}&=(1-w)\alpha_{K}(v)-w\beta_{K}(v)= \frac{\phi}{\tau (v)}\bigl[w_{\infty}(v)-w\bigr], \end{aligned} $$

where *w* is the K^+^ gating variable. It is assumed that Ca^2+^ channels are in quasi-steady state $a_{\infty}(v)$, thus eliminating the fraction of open Ca^2+^ channels as a variable. For $i=\mathrm{K},\mathrm{Ca},{\mathrm{L}}$, let $f_{i}=g_{i}(V_{i}-v)$, where $g_{i}$ are ion conductances, and $V_{i}$ are reversal potentials. Opening and closing rates of ion channels depend only on membrane potential *v* are represented by *α* and *β*, respectively, so that

3.6 $$ a_{\infty}(v)=\frac{\alpha_{\mathrm{Ca}}(v)}{\alpha_{\mathrm{Ca}}(v)+\beta_{\mathrm{Ca}}(v)}. $$

For the ML model,

3.7 $$ \alpha_{\mathrm{Ca}}(v)=\beta_{\mathrm{Ca}} \exp \biggl( \frac {2[v-v_{\mathrm{Ca},1}]}{v_{\mathrm{Ca},2}} \biggr) $$

with $\beta_{\mathrm{Ca}}$, $v_{\mathrm{Ca},1}$, $v_{\mathrm{Ca}2}$ constant. The transition rates $\alpha_{\mathrm{K}}(v)$ and $\beta_{\mathrm{K}}(v)$ are chosen such that

3.8 $$ w_{\infty}(v)=\frac{1}{2} \biggl(1+\mbox{tanh} \biggl[ \frac {v-v_{\mathrm{K},1}}{v_{\mathrm{K},2}} \biggr] \biggr), \tau(v)=\cosh \biggl[ \frac {v-v_{\mathrm{K},1}}{2v_{\mathrm{K},2}} \biggr]. $$

**Fig. 4:**
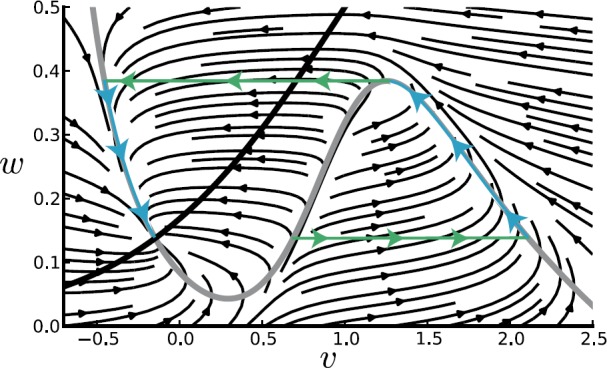
Deterministic phase plane dynamics (adapted from [114]). Thick curves show the nullclines: $\dot{v} = 0$ as grey and $\dot{w}=0$ as black. Black stream lines represent deterministic trajectories. Green/blue curves represent an action potential trajectory in the limit of slow *w*. Parameter values are $C_{m}=20~\mbox{mF}$, $V_{\mathrm{Ca}}=120~\mbox{mV}$, $V_{\mathrm{K}}=-84~\mbox{mV}$, $V_{\mathrm{L}}=-60~\mbox{mV}$, $g_{\mathrm{Ca}}=4.4~\mbox{mS}$, $g_{\mathrm{K}}=8~\mbox{mS}$, $g_{\mathrm{L}}= 2.2~\mbox{mS}$, $\beta_{\mathrm{Ca}}=0.8~\mbox{s}^{-1}$, $v_{\mathrm{Ca}, 1}=-1.2~\mbox{mV}$, $v_{\mathrm{Ca},2}=18~\mbox{mV}$, $v_{\mathrm{K},1}= 2~\mbox{mV}$, $v_{\mathrm{K},2}=30~\mbox{mV}$, and $\phi=0.04~\mbox{ms}^{-1}$

The dynamics of this system can be explored using phase-plane analysis as illustrated in Fig. 4 for an excitable regime. Exploiting the fact that the K^+^ dynamics is much slower than the voltage and Ca^2+^ dynamics, we can use a slow/fast analysis to investigate the initiation of an action potential following a perturbing stimulus [81]. The ML model can also support oscillatory solutions; see also Sect. 6.

#### Stochastic Model

The deterministic ML model holds under the assumption that the number of ion channels is very large, thus the ion channel activation can be approximated by the average ionic currents. However, it is known that channel noise does affect membrane potential fluctuations and thus neural function [146]. To account for ion channel fluctuations, we consider a stochastic version of the ML model [80, 114, 132], in which the number *N* of Ca^2+^ channels is taken to be relatively small. (For simplicity, we ignore fluctuations in the K^+^ channels by taking the number of the latter to be very large.) Let $n(t)$ be the number of open Ca^2+^ channels at time *t*, which means that there are $N-n(t)$ closed channels. The voltage and recovery variables then evolve according to the following PDMP:

3.9 $$ \begin{aligned} C_{m}\frac{dv}{dt}&= \frac{n}{N}f_{\mathrm{Ca}}(v) +wf_{\mathrm{K}}(v)+f_{\mathrm{L}}(v)+I_{\mathrm{app}}, \\ \frac{dw}{dt}&=\frac{\phi}{\tau(v)}\bigl[w_{\infty}(v)-w\bigr] \end{aligned} $$

for $n(t)=n$. Suppose that individual channels switch between open (O) and closed (C) states via a two-state Markov chain,

3.10 $$ \mathrm{C} \mathrel{\mathop{\rightleftarrows}^{\mathrm{\alpha_{Ca}(v)/\varepsilon }}_{\mathrm{\beta_{Ca}/\varepsilon }}} \mathrm{O}. $$

It follows that at the population level, the number of open ion channels evolves according to a birth–death process with

3.11 $$ \begin{aligned} n &\rightarrow n-1, \quad \omega_{n}^{-}(v)=n \beta_{\mathrm{Ca}}/\varepsilon , \\ n &\rightarrow n+1, \quad \omega_{n}^{+}(v)=(N-n) \alpha_{\mathrm{Ca}}(v)/ \varepsilon . \end{aligned} $$

Note that we have introduced the small parameter *ε* to reflect the fact that Ca^2+^ channels open and close much faster than the relaxation dynamics of the system $(v,w)$. This is consistent with the parameter values of the ML model, where the slowness of the K^+^ channels is reflected by the fact that the parameter $\phi =0.04~\mbox{ms}^{-1}$, the membrane rate constant is of order $0.05~\mbox{ms}^{-1}$, whereas the transition rates of Ca^2+^ or Na^+^ channels are of order $1~\mbox{ms}^{-1}$. The stationary density of the birth–death process is

3.12 $$ \rho_{n}(v)=\frac{N!}{n!(N-n)!}\frac{\alpha_{\mathrm{Ca}}^{n}(v)\beta _{\mathrm{Ca}}^{(N-n)}}{(\alpha_{\mathrm{Ca}}(v)+\beta_{\mathrm{Ca}})^{N}}. $$

The corresponding CK equation is

3.13 $$\begin{aligned} \frac{\partial p_{n} }{\partial t} &= -\frac{\partial}{\partial v} \biggl[ \biggl( \frac{n}{N}f_{\mathrm{Ca}}(v)+wf_{\mathrm{K}}(v)+f_{\mathrm{L}}(v)+I_{\mathrm{app}} \biggr) p_{n}(v,w,t) \biggr] \\ &\quad {} -\frac{\partial}{\partial w} \bigl[ \bigl( (1-w)\alpha _{\mathrm{K}}(v)-w \beta_{\mathrm{K}} \bigr) p_{n}(v,w,t) \bigr] \\ &\quad {}+\frac{1}{\varepsilon } \bigl( \omega_{n-1}^{+}(v)p_{n-1}(v,w,t)+ \omega _{n+1}^{-}(v)p_{n+1}(v,w,t) \bigr) \\ &\quad {} - \frac{1}{\varepsilon } \bigl( \bigl(\omega_{n}^{+}(v) + \omega _{n}^{-}(v)\bigr)p_{n}(v,w,t) \bigr) . \end{aligned}$$

Comparison with the general CK equations (2.6) shows that $x=(v,w)$, $\nabla= (\partial_{v},\partial_{w})^{\top}$,

$$ F_{n}(v,w):=\left ( \begin{matrix} f_{n}(v,w) \\ f(v,w) \end{matrix} \right ) =\left ( \begin{matrix} {n}f_{\mathrm{Ca}}(v)/N+wf_{\mathrm{K}}(v)+f_{\mathrm{L}}(v)+I_{\mathrm{app}}\\ (1-w)\alpha_{\mathrm{K}}(v)-w\beta_{\mathrm{K}} \end{matrix} \right ), $$

and **A** is the tridiagonal generator matrix of the birth–death process. Carrying out the QSS diffusion approximation of Sect. 2.2 then yields the following Ito FP equation for $C(v,w,t)=\sum_{n=0}^{N}p_{n}(v,w,t)$ (see also [27]):

3.14 $$ \frac{\partial C}{\partial t} = -\frac{\partial}{\partial v} \bigl[f_{n}(v,w)C \bigr] -\frac{\partial}{\partial w} \bigl[f(v,w)C\bigr]-\varepsilon \frac {\partial}{\partial v} \bigl[{ \mathscr {V}}(v,w)C\bigr]+\varepsilon \frac{\partial ^{2} D(v) C}{\partial v^{2}} $$

with

3.15a $$\begin{aligned} {\mathscr {V}} =& \sum_{m,n} \biggl( \overline{f}(v,w) \frac{\partial }{\partial v}\biggl[\rho_{n}(v)A_{mn}^{\dagger}(v)f_{m}(v,w) \\ &{}- \rho_{n}(v) f_{n}(v,w) \frac{\partial}{\partial v} \bigl(A_{mn}^{\dagger}(v)f_{m}(v,w) \bigr)\biggr] \biggr) \end{aligned}$$

and

3.15b $$\begin{aligned} D&=\sum_{m,n}\bigl[f_{m}(v,w)- \overline{f}(v,w)\bigr]A^{\dagger}_{mn}(v)\rho _{n}(v) \bigl[\overline{f}(v,w)-f_{n}(v,w)\bigr] \\ &=\sum_{m,n} \biggl[\frac{m-\langle m\rangle}{N}f_{\mathrm{Ca}}(v) \biggr]A^{\dagger}_{mn}(v)\rho_{n}(v) \biggl[ \frac{\langle n\rangle -n}{N}f_{\mathrm{Ca}}(v) \biggr] \\ &=\frac{1}{N}f_{\mathrm{Ca}}(v)^{2}a_{\infty}(v) \bigl[1-a_{\infty}(v)\bigr]^{2}. \end{aligned}$$

The last line follows from a calculation in [80].

Almost all previous studies of ion channel fluctuations are based on some form of diffusion approximation, thus reducing the continuous dynamics to an effective Langevin equation [32, 54, 64, 146]. However, these various approximations can lead to exponentially large errors in estimates for quantities such as the rate at which noise-driven action potentials are generated in the excitable regime. This has motivated recent work that deals directly with the CK equation (3.13). For example, Keener and Newby [80, 115] consider the simplified problem of how ion channel fluctuations affect the initiation of an action potential due to the opening of a finite number of Ca^2+^ or Na^+^ channels. The slow K^+^ channels are assumed to be frozen, so that they effectively act as a leak current, and each sodium channel is treated as a single activating subunit. The recovery variable *w* is thus fixed so the potassium current can be absorbed into the function $g(v):=-[wf_{\mathrm{K}}(v)+f_{\mathrm{L}}(v)+I_{\mathrm{app}}]$. We then have the one-dimensional PDMP

3.16 $$ \frac{dv}{dt}=\frac{n}{N}f_{\mathrm{Ca}}(v)-g(v), $$

and the CK equation (3.13) reduces to

3.17 $$\begin{aligned} \frac{\partial p_{n} }{\partial t} &= -\frac{\partial}{\partial v} \biggl( \frac{n}{N}f_{\mathrm{Ca}}(v)-g(v) \biggr) p_{n}(v,t) \\ &\quad {}+\frac{1}{\varepsilon } \bigl( \omega_{n-1}^{+}(v)p_{n-1}(v,t)+ \omega _{n+1}^{-}(v)p_{n+1}(v,t) \bigr) \\ &\quad {} - \frac{1}{\varepsilon } \bigl(\omega_{n}^{+}(v) + \omega_{n}^{-}(v) \bigr)p_{n}(v,t). \end{aligned}$$

Since the right-hand side of equation (3.16) is negative (positive) for large (small) *v*, it follows that there exists an invariant interval for the voltage dynamics. In particular, let $v_{0}$ denote the voltage for which $\dot{v}=0$ when $n=0$, and let $v_{N}$ be the corresponding voltage when $n=N$, that is, $g(v_{0})=0$ and $f_{\mathrm{Ca}}(v_{N})-g(v_{N})=0$. Then $v(t)\in[v_{0},v_{N}]$ if $v(0)\in[v_{0},v_{N}]$. In the fast switching limit $\varepsilon \rightarrow0$, we obtain the first-order deterministic rate equation

3.18 $$ \frac{dv}{dt}=a_{\infty}(v)f_{\mathrm{Ca}}(v)-g(v) \equiv-\frac{d\varPsi}{dv}. $$

**Fig. 5:**
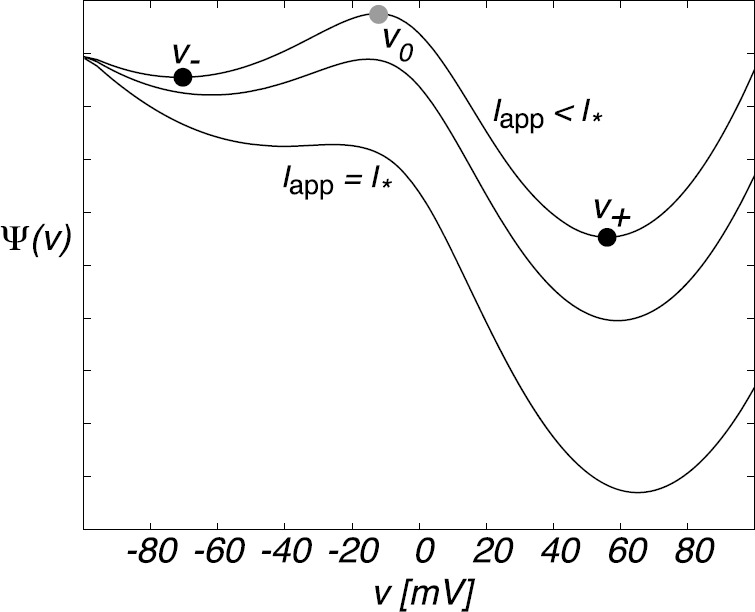
Sketch of deterministic potential $\varPsi(v)$ as a function of voltage *v* for different values of the applied stimulus current $I_{\mathrm{app}}$. At a critical current $I_{*}$, the deterministic system switches from a bistable to a monostable regime, that is, $I^{*}$ is the threshold current for action potential generation

We have introduced the effective potential $\varPsi(v)$ whose minima and maxima correspond to stable and unstable fixed points of the mean-field equation. By plotting the potential *Ψ*, it is straightforward to show that equation (3.18) exhibits bistability for a range of stimuli $I_{\mathrm{app}}$, that is, there exist two stable fixed points $v_{\pm}$ separated by an unstable fixed point $v_{0}$; see Fig. 5. The problem of the spontaneous initiation of an action potential for small but finite *ε* thus reduces to an escape problem for a stochastic hybrid system, as outlined in Sect. 2.3.

### Metastability in the Stochastic Ion Channel Model

To calculate the mean escape rate from the resting state $v_{-}$ using the Arrhenius formula (2.48), we take $v\rightarrow x$ and calculate the functions $\varPhi_{0}(x)$, $k(x)$, and $D(x)$. In the case of the stochastic ion channel model, equation (2.39) takes the explicit form

3.19 $$\begin{gathered} (N-n+1)\alpha R_{n-1} -\bigl[\varLambda_{0}+n\beta+(N-n)\alpha \bigr]R_{n} +(n+1)\beta R_{n+1} \\ \quad =-q \biggl( \frac{n}{N}f-g \biggr)R_{n} . \end{gathered}$$

Consider the trial solution

3.20 $$ R_{n}(x,q)=\frac{\varGamma(x,q)^{n}}{(N-n)!n!}, $$

which yields the following equation relating *Γ* and $\varLambda_{0}$:

$$ \frac{n\alpha}{\varGamma}+\varGamma\beta(N-n)-\varLambda_{0} -n\beta -(N-n) \alpha=-q \biggl(\frac{n}{N}f-g \biggr). $$

Collecting terms independent of *n* and terms linear in *n* yields the pair of equations

3.21 $$ q=-\frac{N}{f(x)} \biggl(\frac{1}{\varGamma(x,q)}+1 \biggr) \bigl(\alpha(x)- \beta(x) \varGamma(x,q) \bigr) $$

and

3.22 $$ \varLambda_{0}(x,q)=-N\bigl(\alpha(x)-\varGamma(x,q) \beta(x)\bigr)-qg(x). $$

Eliminating *Γ* from these equation gives

$$ q=\frac{1}{f(x)} \biggl( \frac{N\beta(x)}{\varLambda_{0}(x,q)+N\alpha (x)+qg(x)}+1 \biggr) \bigl( \varLambda_{0}(x,q)+qg(x)\bigr). $$

This yields a quadratic equation for $\varLambda_{0}$ of the form

3.23 $$ \varLambda_{0}^{2}+\sigma(x) \varLambda_{0}-h(x,q)=0 $$

with

$$\begin{aligned} \sigma(x)&=\bigl(2g(x)-f(x)\bigr)+N\bigl(\alpha(x)+\beta(x)\bigr), \\ h(x,q)&=q\bigl[-N\beta(x) g(x)+\bigl(N\alpha(x)+qg(x)\bigr) \bigl(f(x)-g(x) \bigr)\bigr]. \end{aligned}$$

Along the zero-energy surface $\varLambda_{0}(x,q)=0$, we have $h(x,q)=0$, which yields the pair of solutions

3.24 $$ q=0 \quad \mbox{and} \quad q=\varPhi_{0}'(x)\equiv-N \frac{\alpha(x) f(x)-(\alpha (x)+\beta)g(x)}{g(x)(f(x)-g(x))}. $$

The normalized eigenfunction for the nontrivial case is

3.25 $$ Z_{n}^{(0)}(x)=\frac{N!}{(N-n)!n!} \frac{(f(x)-g(x))^{N-n}g(x)^{n}}{f(x)^{N}}. $$

**Fig. 6:**
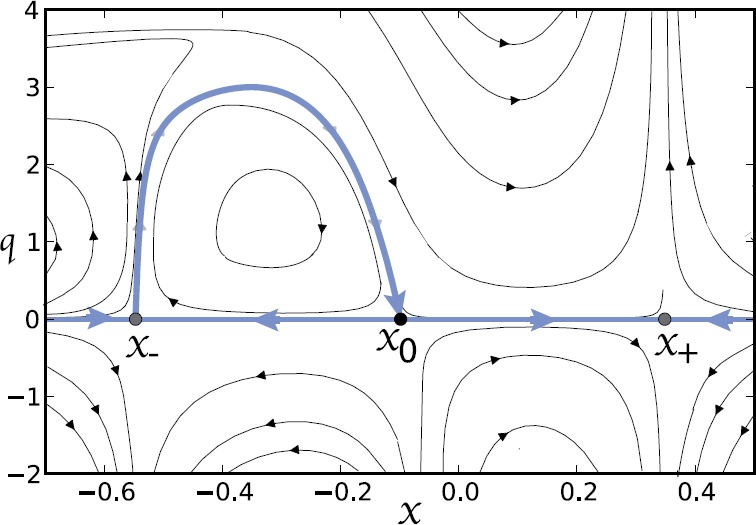
Phase portrait of Hamilton’s equations of motion for the ion channel model with Hamiltonian given by the Perron eigenvalue (3.22). (*x* and *q* are taken to be dimensionless.) The zero energy solution representing the maximum likelihood path of escape from $x_{-}$ is shown as the gray curve. (The corresponding path from $x_{+}$ is not shown.) Same parameter values as Fig. 4

Note that $\varPhi_{0}'(x)$ vanishes at the fixed points $x_{-},x_{0}$ of the mean-field equation (3.18) with $\varPhi_{0}'(x)>0$ for $0< x< x_{-}$ and $\varPhi_{0}'(x)>0 $ for $x_{-}< x< x_{0}$. In Fig. 6, we show solutions to Hamilton’s equations in the $(x,q)$-plane, highlighting the zero-energy maximum likelihood curve linking $x_{-}$ and $x_{0}$. Note that $N\varPhi(x_{0})$, where $\varPhi(x_{0})$ is the area enclosed by the heteroclinic connection from $x_{-}$ to $x_{0}$, gives the leading order contribution to log*τ*, where *τ* is the mean escape time.

The next step is to determine the null eigenfunction $S_{n}(x)$ of equation (2.43), which becomes

$$ (N-m)\alpha S_{m+1}-\bigl[(N-m)\alpha+m\beta\bigr]S_{m}+m \beta S_{m-1} =-\varPhi _{0}' \biggl( \frac{m}{N}f(x)-g(x) \biggr)S_{m} . $$

Trying a solution of the form $S_{m}(x)=\varGamma(x)^{m}$ yields

3.26 $$ (N-m)\alpha\varGamma-\bigl((N-m)\alpha+m\beta\bigr)+m\beta\varGamma^{-1}=- \varPhi _{0}' \biggl(\frac{m}{N}f(x)-g(x) \biggr). $$

*Γ* is then determined by canceling terms independent of *m*:

3.27 $$ S_{n}(x)= \biggl(\frac{\beta g(x)}{\alpha(x)(f(x)-g(x)))} \biggr)^{n}. $$

Finally, a QSS analysis of the CK equation shows that [80]

3.28 $$ D(x_{0}) =\frac{f(x_{0})^{2}\alpha(x_{0})\beta}{N(\alpha(x_{0})+\beta)^{3}}, $$

where have used the fixed point condition $g(x_{0})=f(x_{0})a_{\infty}(x_{0})$.

**Fig. 7:**
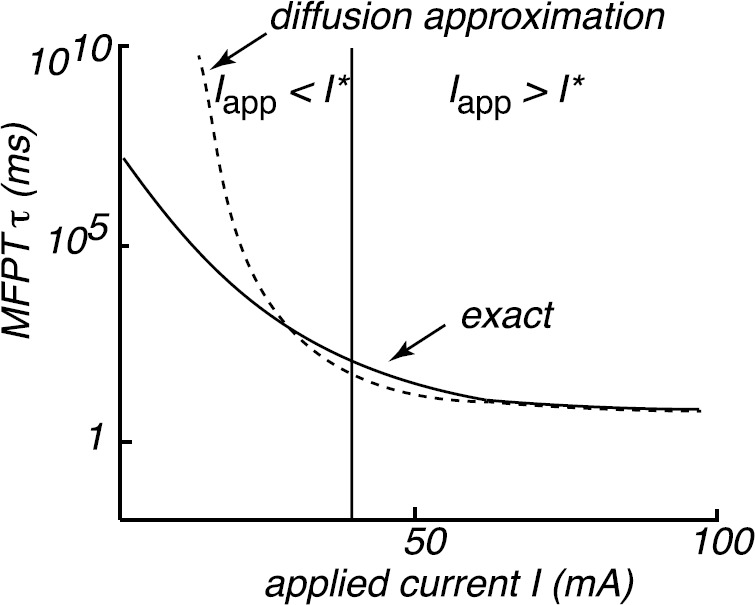
Schematic diagram comparing MFPT calculated using the diffusion approximation with the MFPT of the full system. (Redrawn from [80].) The scales of the axes are based on numerical results for $N=10$. Other parameter values as in Fig. 4

Keener and Newby [80] calculated the MFPT ($\tau= 1/\lambda _{0}$) using equation (2.48) and showed that their results agreed very well with Monte Carlo simulations of the full system, whose probability density evolves according to the CK equation (3.17). A summary of their findings is shown schematically in Fig. 7, together with the corresponding MFPT obtained using a quasi-steady-state diffusion approximation. The main observation is that although the Gaussian-like diffusion approximation does well in the superthreshold regime ($I_{\mathrm{app}}>I_{*}$), it deviates significantly from the full model results in the subthreshold regime $(I_{\mathrm{app}}< I_{*})$, where it overestimates the mean time to spike. This is related to the fact that the effective potential of the steady-state density under the diffusion approximation generates exponentially large errors in the MFPT.

In the above analysis of membrane voltage fluctuations, it was assumed that the potassium channel dynamics could be ignored during initiation of a spontaneous action potential (SAP). This corresponds to keeping the recovery variable *w* fixed. The resulting stochastic bistable model supported the generation of SAPs due to fluctuations in the opening and closing of fast Ca^2+^ or Na^+^ channels. However, it is also possible to generate a SAP due to fluctuations causing several K channels to close simultaneously, effectively decreasing *w*, and thereby causing *v* to rise. It follows that keeping *w* fixed in the stochastic model excludes the latter mechanism, and thus the resulting MFPT calculation underestimates the spontaneous rate of action potentials. To investigate this phenomenon, it is necessary to consider the full stochastic ML model given by equations (3.9) with a multiplicative noise term added to the dynamics of the recovery variable, which takes into account a finite number *M* of potassium ion channels. An additional complication is that the full model is an excitable rather than a bistable system, so it is not straightforward to relate the generation of SAPs with a noise-induced escape problem. Nevertheless, Newby et al. [110, 114] used WKB methods to identify the most probable paths of escape from the resting state and obtained the following results:

(i) The most probable paths of escape dip significantly below the resting value for *w*, indicating a breakdown of the deterministic slow/fast decomposition.
(ii) Escape trajectories all pass through a narrow region of state space (bottleneck or stochastic saddle node) so that, although there is no well-defined separatrix for an excitable system, it is possible to formulate an escape problem by determining the MFPT to reach the bottleneck from the resting state.

## Stochastic Gap Junctions and Randomly Switching Environments

**Fig. 8:**
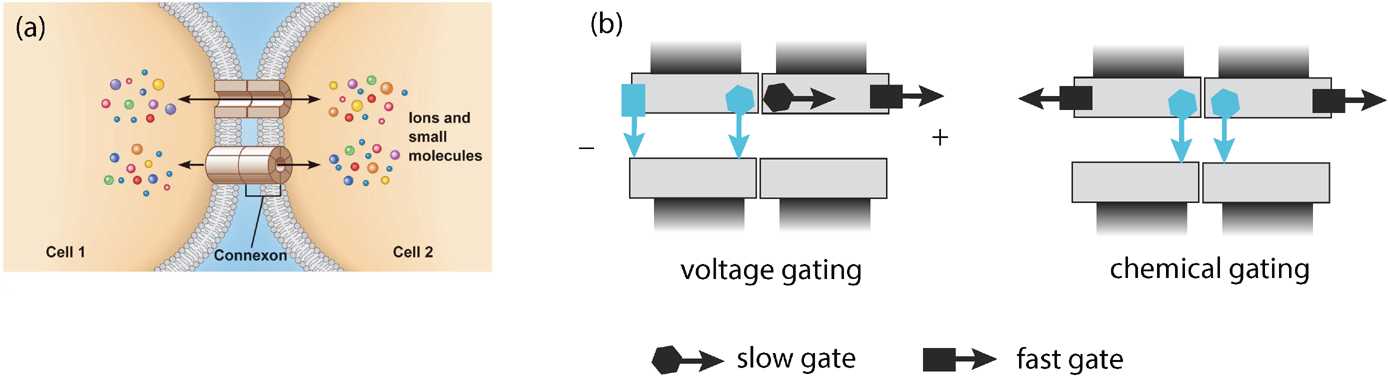
Electrical coupling via gap junctions. (**a**) Schematic diagram of gap junction coupling between two cells. (**b**) Schematic illustration of a Cx43 gap junction channel containing fast (arrow with square) and slow (arrow with hexagon) gates. Voltage gating is mediated by both fast and slow gating mechanisms. Chemical gating is mediated by the slow gating mechanism in both hemichannels

Many neurons in the mammalian central nervous system communicate via gap junctions, also known as electrical synapses [35]. Gap junctions are arrays of transmembrane channels that connect the cytoplasm (aqueous interior) of two neighboring cells and thus provide a direct diffusion pathway for ionic current and small organic molecules to move between cells. In many cases the electrical coupling is strong enough to mediate the synchronization of subthreshold and spiking activity among clusters of neurons. Cells sharing a gap junction channel each provide a hemichannel (also known as a connexon) that connect head-to-head [50, 66, 127]; see Fig. 8(a). Each hemichannel is composed of proteins called connexins that exist as various isoforms named Cx23 through Cx62, with Cx43 being the most common. Just as with the opening and closing of ion channels (see Sect. 2), gap junctions can be gated by both voltage and chemical agents. There appear to be at least two gating mechanisms associated with gap junctions [31], as illustrated in Fig. 8(b). Even when a gap junction is open, it tends to restrict the flow of molecules, and this is typically modeled by assuming that a gap junction has a certain channel permeability [81]. Given that gap junctions are gated, this suggests that thermal fluctuations could result in the stochastic opening and closing of gap junctions in an analogous fashion to ion channels. There has been relatively little work on the effects of thermal noise on gap junction diffusive coupling, beyond modeling the voltage characteristics of a single stochastically-gated gap junction [120]. Recently, however, there have been several studies on analyzing the effective permeability of stochastic gap junctions by formulating the problem as diffusion in a domain with randomly switching internal barriers, which is modeled as a piecewise deterministic PDE [12, 19].

To introduce the basic theory, we begin with the simpler problem of diffusion in a bounded interval with a randomly switching exterior boundary [11, 92]. The latter can represent the random opening and closing of a stochastic ion channel in the plasma membrane of a cell or a subcellular compartment [17].

### Diffusion on an Interval with a Switching Exterior Boundary

**Fig. 9:**
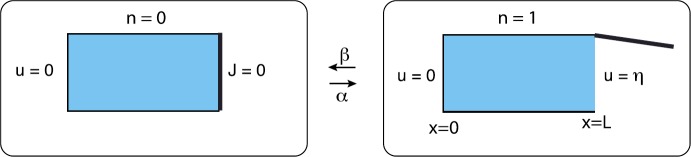
One-dimensional diffusion in a domain with a randomly switching gate on the right-hand side

Consider particles diffusing in the finite interval $[0,L]$ with a fixed absorbing boundary at $x=0$ and a randomly switching gate at $x=L$, see Fig. 9. Let $N(t)\in\{0,1\}$ denote the discrete state of the gate such that it is open when $N(t)=1$ and is closed when $N(t)=0$. Assume that $N(t)$ evolves according to a two-state Markov process with switching rates *α*, *β*:

4.1 $$ \mbox{(closed)}\underset {\beta}{\overset {\alpha}{\rightleftharpoons }} \mbox{(open)}. $$

Consider a particular realization $\sigma(T)=\{N(t), 0\leq t \leq T\}$ of the gate, and let $u(x,t)$ denote the population density of particles in state *x* at time *t* given the realization $\sigma(T)$ up to time *T*. The population density evolves according to the diffusion equation

4.2a $$ \frac{\partial u}{\partial t}=D\frac{\partial^{2}u}{\partial x^{2}}, \quad x\in(0,L), t>0, $$

with *u* satisfying the boundary conditions

4.2b $$ \begin{aligned} u(0,t)&=0, \qquad J(L,t)=0 \quad \mbox{for } N(t)=0, \\ u(L,t)&=\eta\quad \mbox{for } N(t)=1, \end{aligned} $$

and $J(x,t)=-D\partial_{x}u(x,t)$. We are assuming that when the gate is open, the system is in contact with a particle bath of density *η*. Note that equation (4.2a)–(4.2b) only holds between jumps in the state of the gate, so that it is an example of a piecewise deterministic PDE. Since each realization of the gate will typically generate a different solution $u(x,t)$, it follows that $u(x,t)$ is a random field.

#### Derivation of Moment Equations

In [18] a method has been developed for deriving moment equations of the stochastic density $u(x,t)$ in the case of particles diffusing in a domain with randomly switching boundary conditions. The basic approach is to discretize the piecewise deterministic diffusion equation (4.2a)–(4.2b) with respect to space using a finite-difference scheme and then to construct the differential CK equation for the resulting finite-dimensional stochastic hybrid system. One of the nice features of finite-differences is that we can incorporate the boundary conditions into the resulting discrete linear operators. Since the CK equation is linear in the dependent variables, we can derive a closed set of moment equations for the discretized density and then retake the continuum limit. (For an alternative, probabilistic approach to deriving moment equations, see [90].)

The first step is to introduce the lattice spacing *a* such that $(N+1)a=L$ for integer *N* and let $u_{j}=u(aj)$, $j=0,\ldots, N+1$. Then we obtain the PDMP

4.3 $$ \frac{du_{i}}{dt}=\sum_{j=1}^{N} \varDelta ^{n}_{ij}u_{j} +\eta_{a} \delta _{i,N}\delta_{n,1},\quad i=1,\ldots, N, \eta_{a}= \frac{\eta D_{0}}{a^{2}} $$

for $n=0,1$. Away from the boundaries ($i\neq1,N$), $\varDelta ^{n}_{ij}$ is given by the discrete Laplacian

4.4a $$ \varDelta ^{n}_{ij}=\frac{D}{a^{2}}[ \delta_{i,j+1}+\delta_{i,j-1}-2\delta_{i,j}]. $$

On the left-hand absorbing boundary, we have $u_{0}=0$, whereas on the right-hand boundary, we have

$$u_{N+1}=\eta\quad \mbox{for } n=1, \qquad u_{N+1}-u_{N-1}=0 \quad \mbox{for } n=0. $$

These can be implemented by taking

4.4b $$ \varDelta ^{0}_{1j}=\frac{D}{a^{2}}[ \delta_{j,2}-2\delta_{j,1}],\qquad \varDelta ^{1}_{1j}= \frac{D}{a^{2}}[\delta_{j,2}-2\delta_{j,1}] $$

and

4.4c $$ \varDelta ^{0}_{Nj}=\frac{2D}{a^{2}}[ \delta_{N-1,j}-\delta_{N,j}],\qquad \varDelta ^{1}_{Nj}= \frac{D}{a^{2}}[\delta_{N-1,j}-2\delta_{N,j}]. $$

Let $\mathbf {u}(t)=(u_{1}(t),\ldots,u_{N}(t))$ and introduce the probability density

4.5 $$ \operatorname{Prob}\bigl\{ \mathbf {u}(t)\in(\mathbf {u},\mathbf {u}+d\mathbf {u}), N(t)=n\bigr\} =p_{n}(\mathbf {u},t)\, d\mathbf {u}, $$

where we have dropped the explicit dependence on initial conditions. The probability density evolves according to the following differential CK equation for the stochastic hybrid system (4.3) (see Sect. 2.1):

4.6 $$ \frac{\partial p_{n}}{\partial t}=-\sum_{i=1}^{N} \frac{\partial }{\partial u_{i}} \Biggl[ \Biggl(\sum_{j=1}^{N} \varDelta ^{n}_{ij}u_{j}+\eta_{a} \delta_{i,N}\delta_{n,1} \Biggr)p_{n}(\mathbf {u},t) \Biggr]+\sum_{m=0,1}A_{nm}p_{m}( \mathbf {u},t), $$

where *A* is the matrix

4.7 $$ A = \left [ \begin{matrix} -\alpha& \beta\\ \alpha& -\beta \end{matrix} \right ]. $$

Since the drift terms in the CK equation (4.6) are linear in the $u_{j}$, it follows that we can obtain a closed set of equations for the moment hierarchy.

Let

4.8 $$ v_{n,k}(t)=\mathbb {E}\bigl[u_{k}(t)1_{N(t)=n}\bigr]= \int p_{n}(\mathbf {u},t)u_{k}(t)\, d\mathbf {u}. $$

Multiplying both sides of the CK equation (4.6) by $u_{k}(t)$ and integrating with respect to **u** give (after integrating by parts and using that $p_{n}(\mathbf {u},t)\rightarrow0$ as $\mathbf {u}\rightarrow\infty$ by the maximum principle)

4.9 $$ \frac{d v_{n,k}}{d t}=\sum_{j=1}^{N} \varDelta ^{n}_{kj}v_{n,j}+\eta_{a}\rho _{0}\delta_{k,N}\delta_{n,1}+ \sum _{m=0,1}A_{nm}v_{m,k}. $$

We have assumed that the initial discrete state is distributed according to the stationary distribution $\rho_{n}$, so that

$$\int p_{n}(\mathbf {u},t)\, d\mathbf {u}=\rho_{n}. $$

Equations for *r*th-order moments $r\geq2$ can be obtained in a similar fashion. Let

4.10 $$ v^{(r)}_{n,k_{1}\cdots k_{r}}(t)=\mathbb {E}\bigl[u_{k_{1}}(t)\cdots u_{k_{r}}(t)1_{N(t)=n}\bigr]= \int p_{n}(\mathbf {u},t)u_{k_{1}}(t)\cdots u_{k_{r}}(t)\, d\mathbf {u}. $$

Multiplying both sides of the CK equation (4.6) by $u_{k_{1}}(t)\cdots u_{k_{r}}(t)$ and integrating with respect to **u** give (after integration by parts)

4.11 $$\begin{aligned} \frac{d v^{(r)}_{n,k_{1}\cdots k_{r}}}{d t} =&\sum_{l=1}^{r}\sum _{j=1}^{N}\varDelta ^{n}_{k_{l}j}v^{(r)}_{n,k_{1}\cdots k_{l-1}jk_{l+1}\cdots k_{r}}+ \eta_{a} \delta_{n,1}\sum_{l=1}^{r} v^{(r-1)}_{n,k_{1}\cdots k_{l-1}k_{l+1}\cdots k_{r}}\delta_{k_{l},N} \\ &{} +\sum_{m=0,1}A_{nm}v^{(r)}_{m,k_{1}\cdots k_{r}}. \end{aligned}$$

Finally, taking the continuum limit $a\rightarrow0$ in equation (4.9) and setting

4.12 $$ V_{n}(x,t)=\mathbb {E}\bigl[u(x,t)1_{N(t)=n}\bigr], $$

we obtain the first-order moment equations

4.13a $$\begin{aligned} \frac{\partial V_{0}}{\partial t} =&D\frac{\partial^{2} V_{0}}{\partial x^{2}}-\alpha V_{0}+\beta V_{1}, \end{aligned}$$

4.13b $$\begin{aligned} \frac{\partial V_{1}}{\partial t} =& D\frac{\partial^{2}V_{1}}{\partial x^{2} }+\alpha V_{0}-\beta V_{1}, \end{aligned}$$

with

4.14 $$ V_{0}(0,t)=V_{1}(0,t)=0, \qquad \partial_{x}V_{0}(L,t)=0,\qquad V_{1}(L,t)= \rho _{1} \eta>0, $$

and

4.15 $$ \rho_{0}=\frac{\beta}{\alpha+\beta},\qquad \rho_{1}= \frac{\alpha }{\alpha+\beta}. $$

A similar procedure can be used to derive higher-order moment equations [18]. For example, the second-order moments

4.16 $$ C_{n}(x,y,t)=\mathbb {E}\bigl[u(x,t)u(y,t)1_{N(t)=n} \bigr] $$

satisfy the equations

4.17a $$\begin{aligned} \frac{\partial C_{0}}{\partial t} =&D\frac{\partial^{2} C_{0}}{\partial x^{2}}+D_{0}\frac{\partial^{2} C_{0}}{\partial y^{2}}- \alpha C_{0}+\beta C_{1}, \end{aligned}$$

4.17b $$\begin{aligned} \frac{\partial C_{1}}{\partial t} = &D\frac{\partial^{2}C_{1}}{\partial x^{2} }+D_{1}\frac{\partial^{2}C_{1}}{\partial y^{2} }+ \alpha C_{0}-\beta C_{1}, \end{aligned}$$

and couple to the first-order moments via the boundary conditions

4.18a $$ C_{0}(0,y,t)=C_{0}(x,0,t)=C_{1}(x,0,t)=C_{1}(0,y,t)=0 $$

and

4.18b $$ \begin{aligned} \partial_{x}C_{0}(L,y,t)&=\partial_{y}C_{0}(x,L,t)=0, \\ C_{1}(L,y,t)&= \eta V_{1}(y,t), \\ C_{1}(x,L,t)&= \eta V_{1}(x,t). \end{aligned} $$

One of the important points to highlight regarding the stochastic diffusion equation (4.2a)–(4.2b) is that it describes a population of particles diffusing in the same random environment. This means that although the particles are noninteracting, statistical correlations arise at the population level. The inequality follows from the observation that the second-order moment equations are nonseparabale, that is,

$$C_{n}(x,y,t)\neq V_{n}(x,t)V_{n}(y,t). $$

#### Analysis of First-Order Moments

The steady-state solution of equations (4.13a) and (4.13b) can be determined explicitly. First, note that

4.19 $$ \mathbb {E}\bigl[u(x,t)\bigr]=V_{0}(x,t)+V_{1}(x,t). $$

Since equations equations (4.13a) and (4.13b) have a globally attracting steady-state, it follows that

4.20 $$ \lim_{t\rightarrow\infty} \mathbb {E}\bigl[u(x,t)\bigr]=V(x)\equiv\sum _{n=0,1}V_{n}(x), $$

where $V_{n}(x)\equiv\lim_{t\to\infty}V_{n}(x,t)$. Adding equations (4.13a) and (4.13b) and using the boundary conditions in equation (4.14) give

4.21 $$ \frac{d^{2} V}{d x^{2}}=0,\qquad V(0)=0,\qquad V(L)=\rho_{1}\eta+\kappa, $$

where $\kappa=V_{0}(L)$ has to be determined. Hence

$$V(x)=\frac{x}{L}[\rho_{1}\eta+\kappa]. $$

Setting $V_{1}=V-V_{0}$ in equation (4.13a) then shows that

4.22 $$ D\frac{d^{2}V_{0}}{dx^{2}}-(\alpha+\beta)V_{0}=-\frac{\beta}{L} x(\rho _{1}\eta+\kappa) $$

with $V_{0}(0)=0,\partial_{x}V_{0}(L)=0$. It follows that

$$V_{0}(x)=a\mathrm{e}^{-\xi x}+b\mathrm{e}^{\xi x}+ \frac{\rho _{0}}{L}(\rho_{1}\eta+\kappa)x $$

with $\xi=\sqrt{(\alpha+ \beta)/D}$. The boundary conditions imply that

$$a=-b, \qquad 2\xi a \cosh(\xi L)=\frac{\rho_{0}}{L} (\rho_{1}\eta+ \kappa), $$

which yields the solution

4.23 $$ V_{0}(x)=\rho_{0} (\rho_{1}\eta+ \kappa) \biggl[-\frac{1}{\xi L}\frac {\sinh(\xi x)}{\cosh(\xi L)}+\frac{x}{L} \biggr]. $$

Finally, we obtain *κ* by setting $x=L$:

$$\kappa=\rho_{0} (\rho_{1}\eta+\kappa) \bigl[1-(\xi L)^{-1}\tanh(\xi L) \bigr], $$

which can be rearranged to yield

$$\kappa=\rho_{1}\rho_{0}\eta\frac{1-(\xi L)^{-1}\tanh(\xi L)}{\rho _{1}+\rho_{0}(\xi L)^{-1}\tanh(\xi L)}, $$

and thus [11, 92]

4.24 $$ V(x)=\frac{x}{L}\frac{\eta}{{1+(\rho_{0}/\rho_{1})(\xi L)^{-1}\tanh (\xi L)}}. $$

In the limit $\xi\rightarrow\infty$ (fast switching),

$$V(x)=\frac{x}{L}\eta. $$

### Diffusive Flux Along a One-Dimensional Array of Electrically Coupled Neurons

**Fig. 10:**
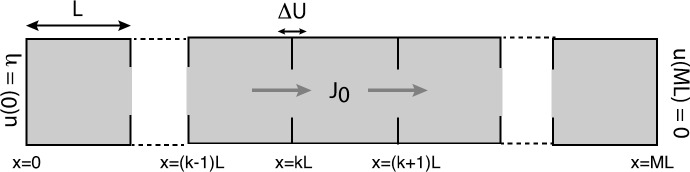
One-dimensional line of cells coupled by gap junctions. At steady-state there is a uniform flux $J_{0}$ through each cell but a jump discontinuity $\varDelta U =-J_{0}/\mu$ in the concentration across each gap junction, where *μ* is the permeability of each junction. See text for details

Let us now consider a simple one-dimensional (1D) model of molecules diffusing along a line of *M* cells that are connected via gap junctions, see Fig. 10. For the moment, we ignore the effects of stochastic gating. Since gap junctions have relatively high resistance to flow compared to the cytoplasm, we assume that each intercellular membrane junction acts like an effective resistive pore with some permeability *μ*. Suppose that we label the cells by an integer *k*, $k=1,\ldots,M$, and take the length of each cell to be *L*. Let $u(x,t)$ for $x\in([k-1]L,kL)$ denote the particle concentration within the interior of the *k*th cell, and assume that it evolves according to the diffusion equation

4.25 $$ \frac{\partial u}{\partial t}=D\frac{\partial^{2} u}{\partial x^{2}}, \quad x\in \bigl([k-1]L,kL\bigr), t>0. $$

However, at each of the intercellular boundaries $x=l_{j}\equiv jL$, $j=1,\ldots,M-1$, the concentration is discontinuous due to the permeability of the gap junctions. Conservation of diffusive flux across each boundary then implies that

4.26 $$ \begin{aligned}[b] -D\frac{\partial u(l_{k}^{-},t)}{\partial x} &=-D\frac{\partial u(l_{k}^{+},t)}{\partial x} \\ &=\mu\bigl[u \bigl(l_{k}^{-},t\bigr)-u\bigl(l_{k}^{+},t\bigr)\bigr], \quad k=1,\ldots,M-1, \end{aligned} $$

where the superscripts + and − indicate that the function values are evaluated as limits from the right and left, respectively. Finally, it is necessary to specify the exterior boundary conditions at $x=0$ and $x=ML$. We impose Dirichlet boundary conditions with $u(0,t)=\eta$ and $u(ML,t)=0$.

In steady-state, there is a constant flux $J_{0}=-DK_{0}$ through the system, and the steady-state concentration takes the form

4.27 $$ u(x)= \textstyle\begin{cases} K_{0}x+\eta, & x\in[0,L), \\ K_{0}(x-[k-1]L)+U_{k}, & x\in([k-1]L,kL), k=2,\ldots,M-1, \\ K_{0}(x-ML), &x\in([M-1]L,ML], \end{cases} $$

for the $M-1$ unknowns $K_{0},U_{k}=u((k-1)L)$, $k=2,\ldots,M-1$. These are determined by imposing the $M-1$ boundary conditions (4.26) in steady state:

4.28a $$\begin{aligned} J_{0} =&\mu[\eta+K_{0}L-U_{2}]= \mu[K_{0}L+U_{2}-U_{3}] \\ =&\cdots= \mu[K_{0}L+U_{M-2}-U_{M-1}], \end{aligned}$$

4.28b $$\begin{aligned} J_{0} =&\mu[2K_{0}L+U_{M-1}]. \end{aligned}$$

Rearranging equations (4.28a) gives

4.29 $$ U_{2}=\eta-\frac{J_{0}L}{D}-\frac{J_{0}}{\mu}, \qquad U_{k}=U_{k-1}-\frac {J_{0}L}{D}-\frac{J_{0}}{\mu}, \quad k=3,\ldots,M-1, $$

which can be iterated to give

$$U_{M-1}=\eta-(M-2)J_{0} \biggl[\frac{L}{D}+ \frac{1}{\mu} \biggr]. $$

Since we also have

$$U_{M-1}=2J_{0} \biggl[\frac{L}{D}+ \frac{1}{\mu} \biggr]-\frac {J_{0}}{\mu}, $$

it follows that [81]

4.30 $$ J_{0}=\frac{D\eta}{ML} \biggl[1+ \frac{D(M-1)}{\mu LM} \biggr]^{-1}. $$

Introducing the effective diffusion coefficient $D_{e}$ according to

4.31 $$ J_{0}=\frac{D_{e}\eta}{ML}, $$

we see that, for large *M*,

4.32 $$ \frac{1}{D_{e}}= \biggl[\frac{1}{D}+\frac{1}{\mu L} \biggr]. $$

### Effective Permeability for Cells Coupled by Stochastically Gated Gap Junctions

This deterministic model has recently been extended to incorporate the effects of stochastically gated gap junctions [12]. The resulting model can be analyzed by extending the theory of diffusion in domains with randomly switching exterior boundaries [18] (see Sect. 4.1) to the case of switching interior boundaries. Solving the resulting first-order moment equations of the stochastic concentration allows us to calculate the mean steady-state concentration and flux, and thus extract the effective single-gate permeability of the gap junctions.

**Fig. 11:**
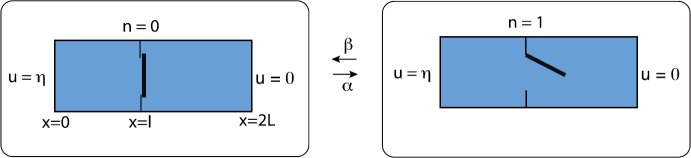
Pair of cells coupled by a stochastically gated gap junction

We start by looking at a pair of stochastically-coupled cells; see Fig. 11. For the sake of generality, we allow the two cells to have different lengths *l* and $2L-l$ with $0< l\leq L$. The basic problem can be formulated as follows: We wish to solve the diffusion equation in the open domain $\varOmega=\varOmega_{1}\cup\varOmega_{2}$ with $\varOmega_{1}=(0,l)$ and $\varOmega_{2}=(l,2L)$, with the interior boundary between the two subdomains at $x=l$ randomly switching between an open and a closed state. Let $N(t)$ denote the discrete state of the gate at time *t* with $N(t)=0$ if the gate is closed and $N(t)=1$ if it is open. Assume that transitions between the two states $n=0,1$ are described by the two-state Markov process (4.1). The random opening and closing of the gate means that particles diffuse in a random environment according to the piecewise deterministic equation

4.33 $$ \frac{\partial u}{\partial t}=D\frac{\partial^{2}u}{\partial x^{2}},\quad x\in \varOmega_{1}\cup\varOmega_{2}, t>0, $$

with *u* satisfying Dirichlet boundary conditions on the exterior boundaries of *Ω*,

4.34 $$ u(0,t)=\eta>0, \qquad u(2L,t)=0, $$

and $N(t)$-dependent boundary conditions on the interior boundary at $x=l$:

4.35 $$ \partial_{x}u\bigl(l^{-},t\bigr)=0=\partial_{x} u\bigl(l^{+},t\bigr) \quad \mbox{for } N(t)=0, $$

and

4.36 $$ u\bigl(l^{-},t\bigr)=u\bigl(l^{+},t\bigr),\qquad \partial_{x}u\bigl(l^{-},t\bigr)=\partial_{x} u\bigl(l^{+},t\bigr) \quad \mbox{for } N(t)=1, $$

where $l^{\pm}=\lim_{\varepsilon \rightarrow0^{+}}l\pm \varepsilon $. That is, when the gate is open, there is continuity of the concentration and the flux across $x=l$, whereas when the gate is closed, the right-hand boundary of $\varOmega_{1}$ and the left-hand boundary of $\varOmega_{2}$ are reflecting. For simplicity, we assume that the diffusion coefficient is the same in both compartments, so that the piecewise nature of the solution is solely due to the switching gate. For illustration, we take the exterior boundary conditions to be Dirichlet, but the analysis is easily modified, for example, in the case of a Neumann boundary condition at one of the ends.

#### First-Order Moment Equations and Effective Permeability ($M=2$)

To determine the effective permeability of a stochastically gated gap junction, we need to calculate the mean of the concentration $u(x,t)$ defined by equation (4.19). The corresponding first-order moment equations for $V_{n}$ can be derived along similar lines to the case of 1D diffusion in a domain with an exterior gate. We thus obtain equations (4.13a) and (4.13b) for $x\in\varOmega_{1}\cup\varOmega_{2}$ with exterior boundary conditions [12]

4.37 $$ V_{0}(0,t)=\rho_{0} \eta, \qquad V_{1}(0,t)=\rho_{1} \eta, \qquad V_{0}(2L,t)= V_{1}(2L,t)=0, $$

and interior boundary conditions

4.38 $$ \begin{aligned} \partial_{x}V_{0}\bigl(l^{-},t\bigr)&=0= \partial_{x}V_{0}\bigl(l^{+},t\bigr), \\ V_{1} \bigl(l^{-},t\bigr)&=V_{1}\bigl(l^{+},t\bigr), \\ \partial_{x} V_{1}\bigl(l^{-},t\bigr)&=\partial_{x}V_{1}\bigl(l^{+},t \bigr). \end{aligned} $$

As in Sect. 4.1, we will analyze the steady-state solution. From the interior boundary conditions (4.38) we set

$$\partial_{x} V_{1}\bigl(l^{-}\bigr)=\partial_{x}V_{1} \bigl(l^{+}\bigr)=K_{1} $$

with $K_{1}$ to be determined later by imposing $V_{1}(l^{-})=V_{1}(l^{+})$. Adding equations (4.13a) and (4.13b) and imposing the boundary conditions then give

4.39 $$ \frac{d^{2} V}{d x^{2}}=0,\quad x\in[0,l),\qquad V(0)=\eta, \qquad \partial_{x} V\bigl(l^{-}\bigr)=K_{1}, $$

and

4.40 $$ \frac{d^{2} V}{d x^{2}}=0, \quad x\in(l,2L],\qquad \partial_{x}V\bigl(l^{+} \bigr)=K_{1}, \qquad V(2L)=0. $$

This yields the piecewise linear solution

4.41 $$ V(x)= \textstyle\begin{cases} K_{1}x+\eta, & x\in[0,l), \\ K_{1}(x-2L), & x\in(l,2L]. \end{cases} $$

Since $V_{1}=V-V_{0}$, we can rewrite equation (4.13a) as

4.42 $$ D\frac{d^{2}V_{0}}{dx^{2}}-(\alpha+\beta)V_{0}(x)=-\beta V(x) $$

with $V_{0}(0)=\rho_{1} \eta$, $V_{0}(2L)=0$, and $\partial _{x}V_{0}(l^{-})=0=\partial_{x}V_{0}(l^{+})$. Substituting for $V(x)$ using equation (4.41), we obtain a piecewise solution of the form

4.43a $$\begin{aligned} V_{1}(x) =&B \sinh(\xi x)+{\rho_{0}(K_{1}x+ \eta)}, \quad x\in[0,l), \end{aligned}$$

4.43b $$\begin{aligned} V_{1}(x) =&C \sinh\bigl([2L-x]\xi\bigr)+{\rho_{0}K_{1}(x-2L)}, \quad x\in(l,2L], \end{aligned}$$

with $\xi=\sqrt{(\alpha+\beta)/D}$. We have imposed the exterior boundary conditions. The interior boundary conditions for $V_{0}$ then determine the coefficients *B*, *C* in terms of $K_{1}$ so that we find

4.44a $$\begin{aligned} V_{0}(x) =&-\frac{\rho_{0}K_{1}}{\xi} \frac{\sinh(\xi x)}{\cosh(\xi l)}+{ \rho_{0}(K_{1}x+\eta)},\quad x\in[0,l), \end{aligned}$$

4.44b $$\begin{aligned} V_{0}(x) =&\frac{\rho_{0}K_{1}}{\xi} \frac{\sinh(\xi[2L-x])}{\cosh(\xi [2L-l])}+{ \rho_{0}K_{1}(x-2L)},\quad x\in(l,2L]. \end{aligned}$$

Finally, we determine the unknown coefficient $K_{1}$ by requiring that $V_{1}(x)$ is continuous across $x=l$, that is,

$$K_{1}l+\eta-V_{0}\bigl(l^{-}\bigr)=K_{1}(l-2L)-V_{0} \bigl(l^{+}\bigr), $$

which yields the result

$$\frac{\rho_{0}K_{1}}{\xi} \bigl[\tanh\bigl(\xi[2L-l]\bigr)+\tanh(\xi l) \bigr]=- \rho_{1} (\eta+2K_{1}L). $$

This can be rearranged to yield the following result for the mean flux through the gate, $J_{0}=-DK_{0}$:

4.45 $$ J_{0}=\frac{D\eta}{2L}\frac{1}{1+(\rho_{0}/\rho_{1})(2\xi L)^{-1} [\tanh(\xi[2L-l])+\tanh(\xi l) ]}. $$

Comparison with equation (4.30) for $M=2$ and $l=L$ implies that the stochastically gated gap junction has the effective permeability $\mu_{e}$ with

4.46 $$ \frac{1}{\mu_{e}}=\frac{2\rho_{0}}{\rho_{1}}\frac{\tanh(\xi L)}{\xi D} . $$

It is useful to note some asymptotic properties of the solution given by equations (4.41) and (4.45). First, in the fast switching limit $\xi\rightarrow\infty$, we have $J_{0}\rightarrow\eta D/2L$, $\mu_{e}\rightarrow\infty$, and equation (4.41) reduces to the continuous steady-state solution

$$V(x)=\frac{\eta(2L-x)}{2L},\quad x\in[0,2L]. $$

The mean flux through the gate is the same as the steady-state flux without a gate. On the other hand, for finite switching rates, the mean flux $J_{0}$ is reduced. In the limit $\alpha\rightarrow0$ (gate always closed), $J_{0}\rightarrow0$, so that $V(x)=\eta$ for $x\in[0,l)$ and $V(x)=0$ for $x\in(l,L]$. Finally, in the limit $l\rightarrow2L$, we recover the result for 1D diffusion in a single domain with a switching external boundary [11, 92] (see also equation (4.24)):

4.47 $$ V(x)=\eta\frac{2L-x}{2L}\frac{1}{{1+(\rho_{0}/\rho_{1})(2\xi L)^{-1}\tanh(2\xi L)}}. $$

#### Multicell Model ($M>2$)

Let us return to the general case of a line of *M* identical cells of length *L* coupled by $M-1$ gap junctions at positions $x=l_{k}=kL$, $1\leq k \leq M-1$; see Fig. 10. (Interestingly, such a model is formally equivalent to a signaling model analyzed in [94].) The analysis is considerably more involved if the gap junctions physically switch because there are significant statistical correlations arising from the fact that all the particles move in the same random environment, which exists in $2^{M-1}$ different states if the gates switch independently [12]. Therefore we will restrict the analysis to the simpler problem in which individual particles independently switch conformational states: if a particle is in state $N(t)=0$, then it cannot pass through a gate, whereas if it is in state $N(t)=1$, then it can. Hence, from the particle perspective, either all gates are open, or all gates are closed. If $V_{n}(x,t)$ is the concentration of particles in state *n*, then we have the pair of PDEs given by equations (4.13a) and (4.13b) on the domain $x\in[0,ML]$, except now the exterior boundary conditions are

4.48 $$ V_{n}(0)=\rho_{n}\eta,\qquad V_{n}(L)=0, \quad n=0,1, $$

and the interior boundary conditions at the *j*th gate are

4.49a $$\begin{aligned} \partial_{x}V_{0}\bigl(l_{j}^{-} \bigr) =&0=\partial_{x}V_{0}\bigl(l_{j}^{+}\bigr), \end{aligned}$$

4.49b $$\begin{aligned} \bigl[V_{1}(x) \bigr]_{x=l_{j}^{-}}^{x=l_{j}^{+}} =&0, \qquad \bigl[\partial _{x}V_{1}(x) \bigr]_{x=l_{j}^{-}}^{x=l_{j}^{+}}=0. \end{aligned}$$

These equations can be solved along similar lines to the two-cell case [12]. This ultimately yields the following expression for the flux $J_{0}$:

4.50 $$ J_{0}=\frac{D\eta}{ML}\frac{1}{1+(\rho_{0}/\rho_{1})(M\xi L)^{-1} [2\tanh(\xi L)+(2M-4)\frac{ \cosh(\xi L)-1}{ \sinh(\xi L)} ]}. $$

We deduce that the effective permeability $\mu_{e}(M)$ in the case of *M* cells with $M-1$ independent, stochastically gated gap junctions is

4.51 $$ \frac{1}{\mu_{e}(M)}=\frac{\rho_{0}}{[M-1]\rho_{1} \xi D} \biggl[2\tanh (\xi L)+(2M-4)\frac{ \cosh(\xi L)-1}{ \sinh (\xi L)} \biggr]. $$

This reduces to equation (4.46) when $M=2$. We conclude that the effective single-gate permeability is *M*-dependent with

$$\lim_{M\rightarrow\infty} \frac{1}{\mu_{e}(M)}= \frac{2\rho _{0}}{\rho_{1} \xi D} \frac{ \cosh(\xi L)-1}{ \sinh(\xi L)}. $$

### Volume Neurotransmission

**Fig. 12:**
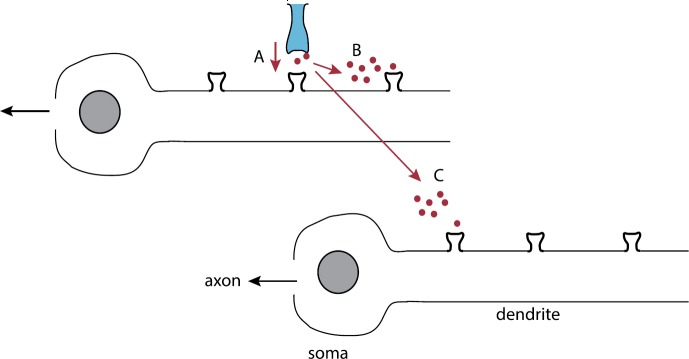
Schematic diagram illustrating volume neurotransmission. Stimulation of an axon terminal contacting a specific synapse on the dendrite of one neuron leads to the release of neurotransmitter within the corresponding synaptic cleft. (**A**) If neurotransmitter uptake is weak, then it is possible for neurotransmitters to diffuse in the extracellular space and subsequently bind to receptors at other synaptic locations of the same neuron (**B**) or of another neuron (**C**)

Although many neurons communicate via synapse-specific connections or gap junctions, it is also possible for populations of neurons to make nonspecific connections via volume neurotransmission [33, 58]; see Fig. 12. For example, neurons may send projections to some distant nucleus or subnucleus, where they increase the concentration of neurotransmitter within the extracellular space surrounding the nucleus. The resulting increase in concentration modulates the electrophysiological neural activity in the distant region by binding of neurotransmitter to receptors on the target cells. One important class of volume transmission involves axonal projections transmitting neuromodulators such as dopamine and serotonin from brain stem nuclei to other brain regions such as the striatum and cortex.

Recently, volume transmission has been formulated as another example of diffusion in a randomly switching environment [91]. Here, the environment is the extracellular volume surrounding the target cells, whereas each axonal terminal acts as a source of neurotransmitter when the source neuron fires and is a sink for neurotransmitter otherwise. The latter is due to the reuptake of neurotransmitter into the terminals. Lawley et al. [91] consider diffusion on a finite interval $[0,L]$ as in Sect. 4.1 but with modified boundary conditions. One example assumes a reflecting boundary at $x=0$ and a switching boundary at $x=L$ due to the presence of a source cell at the right-hand side. The boundary condition thus switches between absorbing when the neuron is not firing (quiescent state $N(t)=0$) and constant flux when the neuron is firing (firing state $N(t)=1$). This yields the system of equations

4.52 $$ \frac{\partial u}{\partial t}=D\frac{\partial^{2}u}{\partial x^{2}},\quad x\in(0,L), t>0, $$

with *u* satisfying the boundary conditions

4.53 $$ \begin{aligned} \partial_{x} u(0,t)&=0, \qquad u(L,t)=0\quad \mbox{for } N(t)=0, \\ \partial_{x}u(L,t)&=c \quad \mbox{for } N(t)=1. \end{aligned} $$

Analysis of the first-order moment equations for $V_{n}(x)=\mathbb {E}[u(x,t)1_{N(t)=n}]$ establishes that in steady-state the total mean concentration $V=V_{0}(x)+V_{1}(x)$ is independent of spatial location *x* with [91]

$$V=c\frac{\mu}{\eta}\mbox{coth}L\eta, $$

where

$$\mu= \frac{\alpha}{\beta},\qquad \eta=\sqrt{\frac{\alpha+\beta}{D}}. $$

Here *α* is the switching rate from the quiescent state to the firing state, and *β* is the switching rate of the reverse transition. Thus we observe the same mean concentration *V* throughout the extracellular domain, even though some parts are further away from the source than others. Consistent with intuition, *V* increases with *μ*, which reflects the fact that the neuron on the boundary fires more often. Now suppose that both *α* and *β* become large (fast switching) but their ratio *μ* is fixed. In this case, *η* becomes large, and $V\rightarrow0$. This is due to the fact that any neurotransmitter that is released is rapidly reabsorbed at the same terminal. (Note that if the left-hand boundary is taken to be absorbing rather than reflecting, $u(0,t)=0$, then the concentration is a linear function of *x*; this could represent a glial cell on the left-hand boundary, which absorbs neurotransmitter but does not fire.) The authors also consider the case where there is a source neuron at each end, so that each boundary switches according to an independent two-state Markov process. If we denote the two Markov processes by the discrete variables $M(t)\in\{0,1\}$ and $N(t)\in\{0,1\}$, respectively, then the boundary conditions become [91]

4.54 $$ u(0,t)=0 \quad \mbox{for } M(t)=0,\qquad \partial_{x}u(0,t)=-c_{0} \quad \mbox{for } M(t)=1, $$

and

4.55 $$ u(L,t)=0\quad \mbox{for } N(t)=0, \qquad \partial_{x}u(L,t)=c_{L} \quad \mbox{for } N(t)=1. $$

Now we find that the mean concentration approaches a uniform concentration, provided that the two neurons are identical; otherwise, the concentration is a linear function of *x*.

## Stochastic Vesicular Transport in Axons and Dendrites

The efficient delivery of mRNA, proteins, and other molecular products to their correct location within a cell (intracellular transport) is of fundamental importance to normal cellular function and development [1, 23]. The challenges of intracellular transport are particularly acute for neurons, which are amongst the largest and most complex cells in biology, in particular, with regards to the efficient trafficking of newly synthesized proteins from the cell body or soma to distant locations on the axon and dendrites. In healthy cells, the regulation of mRNA and protein trafficking within a neuron provides an important mechanism for modifying the strength of synaptic connections between neurons [9, 34, 72, 139], and synaptic plasticity is generally believed to be the cellular substrate of learning and memory. On the other hand, various types of dysfunction in protein trafficking appear to be a major contributory factor to a number of neurodegenerative diseases associated with memory loss, including Alzheimer’s [38].

Broadly speaking, there are two basic mechanisms for intracellular transport: passive diffusion within the cytosol or the surrounding plasma membrane of the cell, and active motor-driven transport along polymerized filaments such as microtubules and F-actin that comprise the cytoskeleton. Newly synthesized products from the nucleus are mainly transported to other intracellular compartments or the cell membrane via a microtubular network that projects radially from organizing centres (centrosomes) and forms parallel fiber bundles within axons and dendrites. The same network is used to transport degraded cell products back to the nucleus. Moreover, various animal viruses including HIV take advantage of microtubule-based transport in order to reach the nucleus from the cell surface and release their genome through nuclear pores [36]. Microtubules are polarized filaments with biophysically distinct plus and minus ends. In general, a given molecular motor will move with a bias toward a specific end of the microtubule; for example, kinesin moves toward the (+) end and dynein moves toward the (−) end. Microtubules are arranged throughout an axon or dendrite with a distribution of polarities: in axons and distal dendrites, they are aligned with the (−) ends pointing to the soma (plus-end-out), and in proximal dendrites, they have mixed polarity.

Axons of neurons can extend up to 1 m in large organisms, but synthesis of many of its components occurs in the cell body. Axonal transport is typically divided into three main categories based upon the observed speed [29]: fast transport (1–9 *μ*m/s) of organelles and vesicles and slow transport (0.004–0.6 *μ*m/s) of soluble proteins and cytoskeletal elements. Slow transport is further divided into two groups; actin and actin-bound proteins are transported in slow component A, whereas cytoskeletal polymers such as microtubules and neurofilaments are transported in slow component B. It had originally been assumed that the differences between fast and slow components were due to differences in transport mechanisms, but direct experimental observations now indicate that they all involve fast motors but differ in how the motors are regulated. Membranous organelles, which function primarily to deliver membrane and protein components to sites along the axon and at the axon tip, move rapidly in a unidirectional manner, pausing only briefly. In other words, they have a high duty ratio—the proportion of time a cargo complex is actually moving. On the other hand, cytoskeletal polymers and mitochondria move in an intermittent and bidirectional manner, pausing more often and for longer time intervals, and sometimes reversing direction. Such a transport has a low duty ratio.

**Fig. 13:**
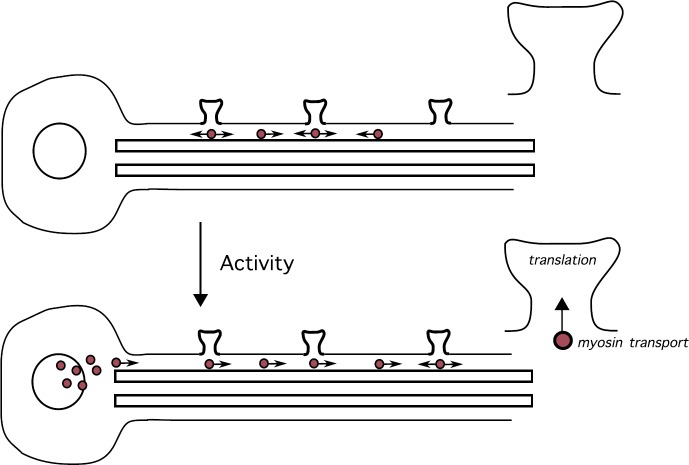
Schematic diagram illustrating mRNA granule mobility in dendrites. Under basal conditions, most granules are either stationary (or exhibit localized oscillations), whereas a minority exhibit bidirectional transport. Depolarization by bathing in extracellular KCl solution activates transcription of mRNA at the cell body and converts existing stationary granules into anterograde granules [125]

Another example of a transport process in neurons that exhibits bidirectionality is the trafficking of mRNA containing granules within dendrites. There is increasing experimental evidence that local protein synthesis in the dendrites of neurons plays a crucial role in mediating persistent changes in synaptic structure and function, which are thought to be the cellular substrates of long-term memory [8, 82, 133]. This is consistent with the discovery that various mRNA species and important components of the translational machinery, such as ribosomes, are distributed in dendrites. Although many of the details concerning mRNA transport and localization are still unclear, a basic model is emerging. First, newly transcribed mRNA within the nucleus binds to proteins that inhibit translation, thus allowing the mRNA to be sequestered away from the protein-synthetic machinery within the cell body. The repressed mRNAs are then packaged into ribonucleoprotein granules that are subsequently transported into the dendrite via kinesin and dynein motors along microtubules. Finally, the mRNA is localized to an activated synapse by actin-based myosin motor proteins, and local translation is initiated following neutralization of the repressive mRNA-binding protein. Details regarding the motor-driven transport of mRNA granules in dendrites have been obtained by fluorescently labeling either the mRNA or mRNA-binding proteins and using live-cell imaging to track the movement of granules in cultured neurons [44, 86, 125]. It has been found that, under basal conditions, the majority of granules in dendrites are stationary or exhibit small oscillations around a few synaptic sites. However, other granules exhibit rapid retrograde (toward the cell body) or anterograde (away from the cell body) motion consistent with bidirectional transport along microtubules. These movements can be modified by neuronal activity as illustrated in Fig. 13. In particular, there is an enhancement of dendritically localized mRNA due to a combination of newly transcribed granules being transported into the dendrite, and the conversion of stationary or oscillatory granules already present in the dendrite into anterograde-moving granules.

### Intracellular Transport as a Velocity Jump Process

In terms of the general theme of this review, intracellular transport models are relevant because they consist of a special type of PDMP known as a velocity jump process [57, 112, 113, 122, 123]. In the case of one-dimensional transport along a filament, an individual particle moves according to the piecewise deterministic ODE

5.1 $$ \frac{dx}{dt}=v_{n(t)}, $$

where the discrete random variable $n(t)\in\varGamma$ indexes the current velocity state $v_{n(t)}$. The simplest example is a particle switching between an anterograde state with velocity $v_{1}>0$ and a retrograde state of velocity $v_{0} <0$, so that we have

$$\frac{dx}{dt}=\xi(t)\equiv[v_{1}-v_{0}]n(t)+v_{0}, \quad n(t)\in\{0,1\}. $$

In the physics literature, $\xi(t)$ is called a dichotomous Markov noise process (DMNP); see the review [5]. The corresponding CK equation is

5.2a $$\begin{aligned} \frac{\partial p_{0}}{\partial t} =&-v_{0}\frac{\partial p_{0}}{\partial x}-\alpha p_{0}+\beta p_{1}, \end{aligned}$$

5.2b $$\begin{aligned} \frac{\partial p_{1}}{\partial t} =&-v_{1}\frac{\partial p_{1}}{\partial x}+\alpha p_{0}-\beta p_{1}, \end{aligned}$$

where *α*, *β* are the corresponding switching rates, which can depend on the current position *x*. In applications, we are typically interested in the marginal density $p(x,t)=p_{0}(x,t)+p_{1}(x,t)$, which can be used to calculate moments of *p* such as the mean and variance,

$$\bigl\langle x(t)\bigr\rangle = \int xp(x,t)\,dx,\qquad \operatorname{Var}\bigl[x(t)\bigr]= \int x^{2} p(x,t)\,dx-\bigl\langle x(t)\bigr\rangle ^{2}. $$

In the unbiased case, $v_{1}= v$, $v_{0}=-v$, $\alpha=\beta$, the marginal probability density $p(x,t)$ satisfies the telegrapher’s equation

5.3 $$ \biggl[\frac{\partial^{2}}{\partial t^{2}}+2\alpha\frac{\partial }{\partial t}-v^{2} \frac{\partial^{2}}{\partial x^{2}} \biggr]p(x,t)=0. $$

(The individual densities $p_{0,1}$ satisfy the same equations.) The telegrapher’s equation can be solved explicitly for a variety of initial conditions. More generally, the short-time behavior (for $t\ll 1/\alpha$) is characterized by wave-like propagation with $\langle x(t)\rangle^{2}\sim(vt)^{2}$, whereas the long-time behavior ($t\gg 1/\alpha$) is diffusive with $\langle x^{2}(t)\rangle\sim2Dt$, $D=v^{2}/2\alpha$. As an explicit example, the solution for the initial conditions $p(x,0)=\delta(x)$ and $\partial_{t}p(x,0)=0$ is given by

$$\begin{aligned} p(x,t)&=\frac{e^{-\alpha t}}{2}\bigl[\delta(x-vt)+\delta(x+vt)\bigr] \\ &\quad {}+\frac{\alpha\mathrm{e}^{-\alpha t}}{2v} \biggl[I_{0}\bigl(\alpha\sqrt {t^{2}-x^{2}/v^{2}}\bigr)+\frac{t}{\sqrt{t^{2}-x^{2}/v^{2}}}I_{0} \bigl(\alpha\sqrt {t^{2}-x^{2}/v^{2}}\bigr) \biggr] \\ &\quad {} \times\bigl[\varTheta(x+vt)-\varTheta(x-vt)\bigr], \end{aligned}$$

where $I_{n}$ is the modified Bessel function of *n*th order, and *Θ* is the Heaviside function. The first two terms clearly represent the ballistic propagation of the initial data along characteristics $x=\pm vt$, whereas the Bessel function terms asymptotically approach Gaussians in the large time limit. The steady-state equation for $p(x)$ is simply $p''(x)=0$, which from integrability means that $p(x)=0$ pointwise. This is consistent with the observation that the above explicit solution satisfies $p(x,t)\rightarrow0$ as $t\rightarrow \infty$.

One of the first examples of modeling intracellular transport as a velocity jump process was within the context of the slow axonal transport of neurofilaments [6, 57, 123]. Neurofilaments are space-filling cytoskeletal polymers that increase the cross-sectional area of axons, which then increases the propagation speed of action potentials. Radioisotopic pulse labeling experiments provide information about the transport of neurofilaments at the population level, which takes the form of a slowly moving Gaussian-like wave that spreads out as it propagates distally. Blum and Reed [6] considered the following system on the semiinfinite domain $0\leq x <\infty$:

5.4a $$\begin{aligned} \varepsilon \biggl[\frac{\partial p_{1}}{\partial t} +v\frac{\partial p_{1}}{\partial x} \biggr]&= \sum _{j=1}^{n}A_{1j}p_{j}, \end{aligned}$$

5.4b $$\begin{aligned} \varepsilon \frac{\partial p_{i}}{\partial t}&= \sum_{j=1}^{n}A_{ij}p_{j} ,\quad 1< i\leq N, \end{aligned}$$

where $p_{1}$ represents the concentration of moving neurofilament proteins, and $p_{i}$, $i>1$, represent the concentrations in $n-1$ distinct stationary states. In contrast to the two-state model of bidirectional transport, the system jumps between a single anterograde state and a set of stationary states. Conservation of mass implies that $A_{jj}=-\sum_{i\neq j}A_{ij}$. The initial condition is $p_{i}(x,0)=0$ for all $1\leq i \leq n$, $0< x<\infty$. Moreover $p_{1}(0,t)=1$ for $t >0$. Reed et al. [123] carried out an asymptotic analysis of equations (5.4a)–(5.4b) that is related to the QSS reduction method of Sect. 2.2. Suppose that $p_{1}$ is written in the form

$$p_{1}(x,t)=Q_{\varepsilon } \biggl(\frac{x-ut}{\sqrt{ \varepsilon }},t \biggr), $$

where *u* is the effective speed, $u=v{p_{1}^{\mathrm{ss}}}/{\sum_{j=1}^{n}p_{j}^{\mathrm{ss}}}$, and $\mathbf{p}^{\mathrm{ss}}$ is the steady-state solution for which $\mathbf{A}\mathbf{p}^{\mathrm{ss}}=0$. They then showed that $Q_{\varepsilon }(s,t)\rightarrow Q_{0}(s,t)$ as $\varepsilon \rightarrow0$, where $Q_{0}$ is a solution to the diffusion equation

$$\frac{\partial Q_{0}}{\partial t} =D \frac{\partial^{2} Q_{0}}{\partial x^{2}} , \qquad Q_{0}(s,0)=H(-s), $$

with *H* the Heaviside function. The diffusivity *D* can be calculated in terms of *v* and the transition matrix **A**. Hence the propagating and spreading waves observed in experiments could be interpreted as solutions to an effective advection–diffusion equation. More recently, [56, 57] have developed a more rigorous analysis of spreading waves. Note that the large time behavior is consistent with the solution of the diffusion equation obtained in the fast switching limit.

**Fig. 14:**
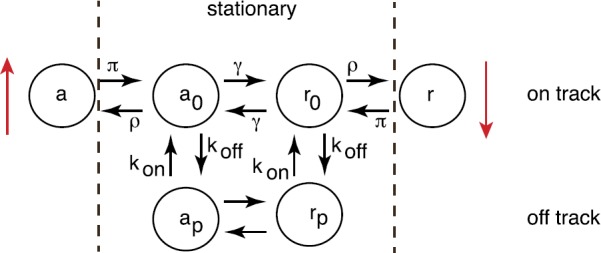
Transition diagram of “stop-and-go” model for the slow axonal transport of neurofilaments. See text for definition of different states

In contrast to these population models, direct observations of neurofilaments in axons of cultured neurons using fluorescence microscopy has demonstrated that individual neurofilaments are actually transported by fast motors but in an intermittent fashion [142]. Hence, it has been proposed that the slow rate of movement of a population is an average of rapid bidirectional movements interrupted by prolonged pauses, the so-called stop-and-go hypothesis [28, 77, 93]. Computational simulations of an associated system of PDEs shows how fast intermittent transport can account for the slowly spreading wave seen at the population level. One version of the model assumes that the neurofilaments can be in one of six states [28, 93]: anterograde moving on track (state *a*), anterograde pausing on track ($a_{0}$ state), anterograde pausing off track (state $a_{p}$), retrograde pausing on track (state $r_{0}$), retrograde pausing off track (state $r_{p}$), and retrograde moving on track (state *r*). The state transition diagram is shown in Fig. 14.

### Tug-of-War Model of Bidirectional Motor Transport

The observation that many types of motor-driven cargo move bidirectionally along microtubules suggests that cargo is transported by multiple kinesin and dynein motors. In proximal dendrites, it is also possible that one or more identical motors move a cargo bidirectionally by switching between microtubules with different polarities. In either case, it is well established that multiple molecular motors often work together as a motor-complex to pull a single cargo [144]. An open question concerns how the set of molecular motors pulling a vesicular cargo are coordinated. One possibility is that the motors compete against each other in a tug-of-war where an individual motor interacts with other motors through the force it exerts on the cargo. If the cargo places a force on a motor in the opposite direction it prefers to move, then it will be more likely to unbind from the microtubule. A recent biophysical model has shown that a tug-of-war can explain the coordinated behavior observed in certain animal models [101, 102].

**Fig. 15:**
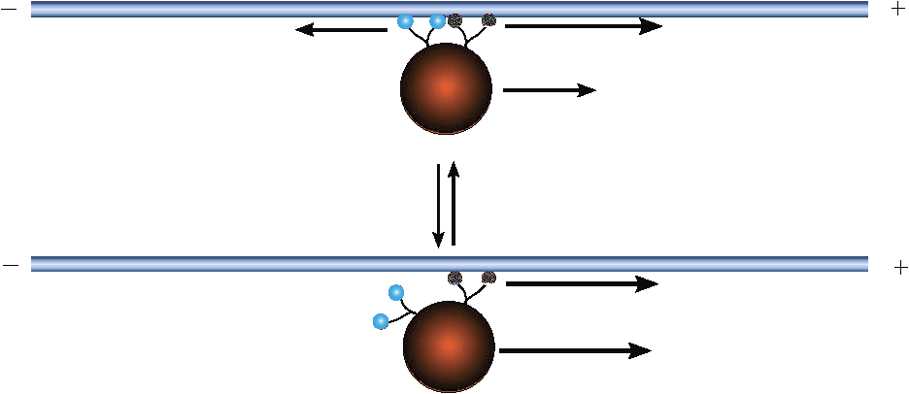
Schematic diagram of an asymmetric tug-of-war model. Two kinesin and two dynein motors transport a cargo in opposite directions along a single polarized microtubule track. Transitions between two possible motor states are shown

Suppose that a certain vesicular cargo is transported along a one-dimensional track via $N_{+}$ right-moving (anterograde) motors and $N_{-}$ left-moving (retrograde motors). At a given time *t*, the internal state of the cargo-motor complex is fully characterized by the numbers $n_{+}$ and $n_{-}$ of anterograde and retrograde motors that are bound to a microtubule and thus actively pulling on the cargo. Assume that over the time-scales of interest all motors are permanently bound to the cargo, so that $0 \leq n_{\pm}\leq N_{\pm}$. The tug-of-war model of Muller et al. [101, 102] assumes that the motors act independently, other than exerting a load on motors with the opposite directional preference. (However, some experimental work suggests that this is an oversimplification, that is, there is some direct coupling between motors [42]). Thus the properties of the motor complex can be determined from the corresponding properties of the individual motors together with a specification of the effective load on each motor. There are two distinct mechanisms whereby such bidirectional transport could be implemented [102]. First, the track could consist of a single polarized microtubule filament (or a chain of such filaments) on which up to $N_{+}$ kinesin motors and $N_{-}$ dynein motors can attach; see Fig. 15. Since individual kinesin and dynein motors have different biophysical properties, with the former tending to exert more force on a load, it follows that even when $N_{+}=N_{-}$, the motion will be biased in the anterograde direction. Hence, this version is referred to as an asymmetric tug-of-war model. Alternatively, the track could consist of two parallel microtubule filaments of opposite polarity such that $N_{+}$ kinesin motors can attach to one filament and $N_{-}$ to the other. In the latter case, if $N_{+}=N_{-}$, then the resulting bidirectional transport is unbiased resulting in a symmetric tug-of-war model.

When bound to a microtubule, the velocity of a single molecular motor decreases approximately linearly with force applied against the movement of the motor [141]. Thus, each kinesin is assumed to satisfy the linear force–velocity relation

5.5 $$ v(F) = \textstyle\begin{cases} v_{f}(1-F/F_{s})& \mbox{for } F\leq F_{s}, \\ v_{b}(1-F/F_{s})& \mbox{for } F\geq F_{s}, \end{cases} $$

where *F* is the applied force in the retrograde direction, $F_{s}$ is the stall force satisfying $v(F_{s})=0$, $v_{f}$ is the forward motor velocity in the absence of an applied force in the preferred direction of the particular motor, and $v_{b}$ is the backward motor velocity when the applied force exceeds the stall force. Dynein motors will also be taken to satisfy a linear force-velocity relation:

5.6 $$ \widehat{v}(F) = \textstyle\begin{cases} \widehat{v}_{f}(1-F/\widehat{F}_{s})& \mbox{for } F\leq\widehat{F}_{s}, \\ \widehat{v}_{b}(1-F/\widehat{F}_{s})& \mbox{for } F\geq\widehat{F}_{s}, \end{cases} $$

where now *F* is the force in the anterograde direction. Since the parameters associated with kinesin and dynein motors are different, we distinguish the latter by taking $F_{s}\rightarrow\widehat{F}_{s}$ etc. The original tug-of-war model assumes that the binding rate of kinesin is independent of the applied force, whereas the unbinding rate is taken to be an exponential function of the applied force:

5.7 $$ \pi(F) = \pi_{0},\qquad \gamma(F) = \gamma_{0}\mathrm{e}^{{F}/{F_{d}}}, $$

where $F_{d}$ is the experimentally measured force scale on which unbinding occurs. The force dependence of the unbinding rate is based on measurements of the walking distance of a single kinesin motor as a function of load [129], in agreement with Kramer’s rate theory [70]. Similarly, for dynein, we take

5.8 $$ \widehat{\pi}(F) = \widehat{\pi}_{0},\qquad \widehat{ \gamma}(F) = \widehat{\gamma}_{0}\mathrm{e}^{{F}/{\widehat{F}_{d}}}. $$

Let $F_{c}$ denote the net load on the set of anterograde motors. Suppose that the molecular motors are not directly coupled to each other, so that they act independently and share the load; however, see [42]. It follows that a single anterograde motor feels the force $F_{c}/n_{+}$. Equation (5.7) implies that the binding and unbinding rates for $n_{+}$ kinesin motors take the form

5.9 $$ \gamma_{+}(n_{+},F_{c}) = n_{+}\gamma(F_{c}/n_{+}), \qquad {\pi}_{+}(n_{+}) = (N_{+}-n_{+})\pi_{0}. $$

Similarly, each dynein motor feels the opposing force $-F_{c}/n_{-}$, so that the binding and unbinding rates for $n_{-}$ dynein motors take the form

5.10 $$ \gamma_{-}(n_{-},F_{c}) = n_{-}\widehat{ \gamma}(F_{c}/n_{-}),\qquad {\pi}_{-}(n_{-}) = (N_{-}-n_{-})\widehat{ \pi}_{0}. $$

The cargo force $F_{c}$ is determined by the condition that all the motors move with the same cargo velocity $v_{c}$. Suppose that the net velocity is in the anterograde direction, which implies $F_{c}/(n_{-}\widehat{F}_{s}) > 1 > F_{c}/(n_{+}F_{s})$. It follows from equations (5.5) and (5.6) that

5.11 $$ v_{c}= v_{f}\bigl(1-F_{c}/(n_{+}F_{s}) \bigr)= -\widehat{v}_{b}\bigl(1-F_{c}/(n_{-} \widehat {F}_{s})\bigr). $$

This generates a unique solution for the load $F_{c}$ and cargo velocity $v_{c}$:

5.12 $$ F_{c}(n_{+},n_{-}) = \bigl({\mathscr {F}} n_{+} F_{s}+ (1-{\mathscr {F}})n_{-}\widehat{F}_{s}\bigr), $$

where

5.13 $$ {\mathscr {F}} =\frac{n_{-}\widehat{F}_{s} v_{f}}{n_{-}\widehat {F}_{s}v_{f}+n_{+}F_{s}\widehat{v}_{b}}, $$

and

5.14 $$ v_{c}(n_{+},n_{-}) = \frac{n_{+}F_{s}-n_{-}\widehat {F}_{s}}{n_{+}F_{s}/v_{f+}+n_{-}\widehat{F}_{s}/\widehat{v}_{b}}. $$

The corresponding expressions when the backward motors are stronger, $n_{+}F_{s+} < n_{-}\widehat{F}_{s}$, are found by interchanging $(v_{f},\widehat{v}_{b})$ with $(\widehat{v}_{f},v_{b})$.

The original study of [101, 102] considered the stochastic dynamics associated with transitions between different internal states $(n_{+},n_{-})$ of the motor complex, without specifying the spatial position of the complex along a 1D track. This defines a Markov process with a corresponding master equation for the time evolution of the probability distribution $P(n_{+},n_{-},t)$. They determined the steady-state probability distribution of internal states and found that the motor complex exhibited at least three different modes of behavior: (i) the motor complex spends most of its time in states with approximately zero velocity; (ii) the motor complex exhibits fast backward and forward movement interrupted by stationary pauses, which is consistent with experimental studies of bidirectional transport; and (iii) the motor complex alternates between fast backward and forward movements. The transitions between these modes of behavior depend on motor strength, which primarily depends upon the stall force. The tug-of-war model can also be formulated as a velocity jump process [112, 113]. This version of the tug-of-war model simultaneously keeps track of the internal state of the motor complex and its location along a 1D track. That is, the position along the track evolves according to piecewise deterministic ODE

5.15 $$ \frac{dx}{dt}=v_{c}\bigl(n_{+}(t),n_{-}(t)\bigr), $$

in between changes in the number of bound kinesin and dynein motors. The various state transitions are

$$\begin{aligned} (n_{+}+1,n_{-})&\underset {\gamma_{+}(n_{+})}{\overset {\pi_{+}(n_{+}+1)}{\rightleftharpoons }}(n_{+},n_{-}),\qquad (n_{+}-1,n_{-}) \underset {\pi_{+}(n_{+})}{\overset {\gamma_{+}(n_{+}-1)}{\rightleftharpoons }}(n_{+},n_{-}), \\ (n_{+},n_{-}+1)&\underset {\gamma_{-}(n_{-})}{\overset {\pi_{-}(n_{-}+1)}{\rightleftharpoons }}(n_{+},n_{-}),\qquad (n_{+},n_{-}-1) \underset {\pi_{-}(n_{-})}{\overset {\gamma_{-}(n_{-}-1)}{\rightleftharpoons }}(n_{+},n_{-}). \end{aligned}$$

As in previous examples, the corresponding CK equation can be reduced to an effective advection–diffusion equation in the limit that the rates of binding and unbinding of molecular motors are sufficiently fast [112, 113].

**Fig. 16:**
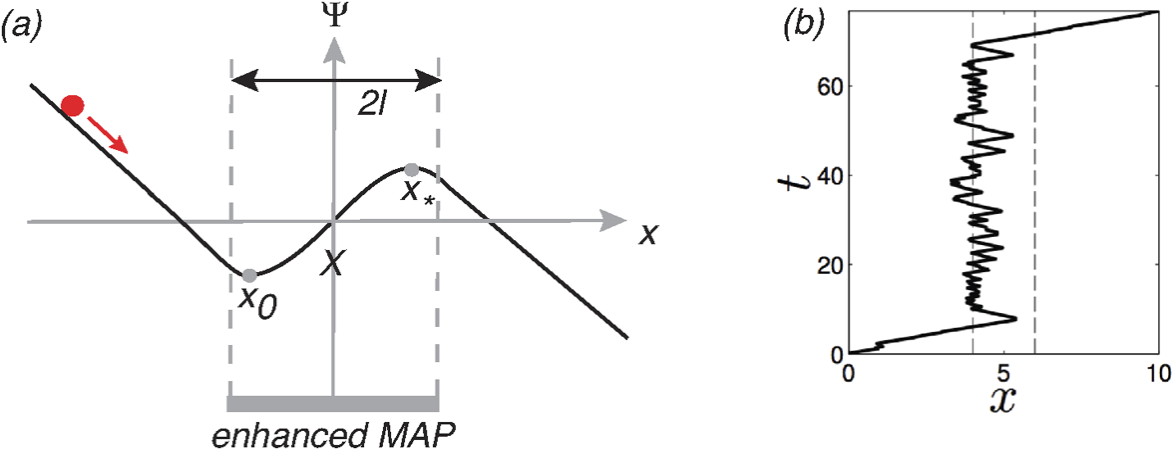
Diagram showing (**a**) the effective potential well created by a region of tau coating an MT, and (**b**) a representative trajectory showing random oscillations within the well. (Adapted from [113])

One of the useful features of the tug-of-war model is that it allows various biophysical processes to be incorporated into the model. For example, a convenient experimental method for changing the stalling force (and hence the mode of motor behavior) is to vary the level of ATP available to the motor complex. At low $[\mathrm {ATP}]$ the motor has little fuel and is weaker, resulting in mode (i) behavior; then, as $[\mathrm {ATP}]$ increases and more fuel is available, mode (ii) behavior is seen until the stall force saturates at high values of $[\mathrm {ATP}]$ where mode (iii) behavior takes over. Thus, $[\mathrm {ATP}]$ provides a single control parameter that tunes the level of intermittent behavior exhibited by a motor complex [112]. Another potentially important signaling mechanism involves microtubule associated proteins (MAPs). These molecules bind to microtubules and effectively modify the free-energy landscape of motor-microtubule interactions [134]. For example, tau is a MAP found in the axon of neurons and is known to be a key player in Alzheimer’s disease [88]. Another important MAP, called MAP2, is similar in structure and function to tau, but is present in dendrites; MAP2 has been shown to affect dendritic cargo transport [95]. Experiments have shown that the presence of tau or MAP2 on the microtubule can significantly alter the dynamics of kinesin; specifically, by reducing the rate at which kinesin binds to the microtubule [140]. This could be implemented by taking the binding rate $\gamma_{0}$ of kinesin to decrease within the domain of enhanced MAP concentration. This means that in the fast switching limit, we obtain the deterministic equation (2.8) with $\overline{F}(x)$ corresponding to an *x*-dependent mean velocity. Suppose, for example, that $\overline{F}(x)=\bar{v}>0$ for $x\notin [X-l,X+l]$ and $\overline{F}(x)$ a unimodal function for $x\in [X-l,X+l]$ with a negative minimum at $x=X$. Here we are taking the region of enhanced *τ* to be an interval of length 2*l* centered about $x=X$. Writing $\overline{F}(x)=-\varPsi'(x-X)$, the corresponding deterministic potential has the form shown in Fig. 16. Since the mean velocity switches sign within the domain $[X-l,X+l]$, it follows that there exists one stable fixed point $x_{0}$ and an unstable fixed point $x_{*}$.

One interesting effect of a local increase in MAPs is that it can generate stochastic oscillations in the motion of the motor-complex [113]. As a kinesin driven cargo encounters the MAP-coated trapping region, the motors unbind at their usual rate and can’t rebind. Once the dynein motors are strong enough to pull the remaining kinesin motors off the microtubule, the motor-complex quickly transitions to (−) end directed transport. After the dynein-driven cargo leaves the MAP-coated region, kinesin motors can then reestablish (+) end directed transport until the motor-complex returns to the MAP-coated region. This process repeats until the motor-complex is able to move forward past the MAP-coated region. Interestingly, particle tracking experiments have observed oscillatory behavior during mRNA transport in dendrites [44, 125]. In these experiments, motor-driven mRNA granules move rapidly until encountering a fixed location along the dendrite where they slightly overshoot then stop, move backward, and begin to randomly oscillate back and forth. After a period of time, lasting on the order of minutes, the motor-driven mRNA stops oscillating and resumes fast ballistic motion. Calculating the mean time to escape, the target can be formulated as an FPT problem, in which the particle starts at $x=x_{0}$ and has to make a rare transition to the unstable fixed point at $x=x_{*}$. As in the analogous problem of stochastic action potential generation (Sect. 3), the QSS diffusion approximation breaks down for small *ε*, and we have to use the asymptotic methods of Sect. 2.3. The details can be found elsewhere [115].

**Fig. 17:**
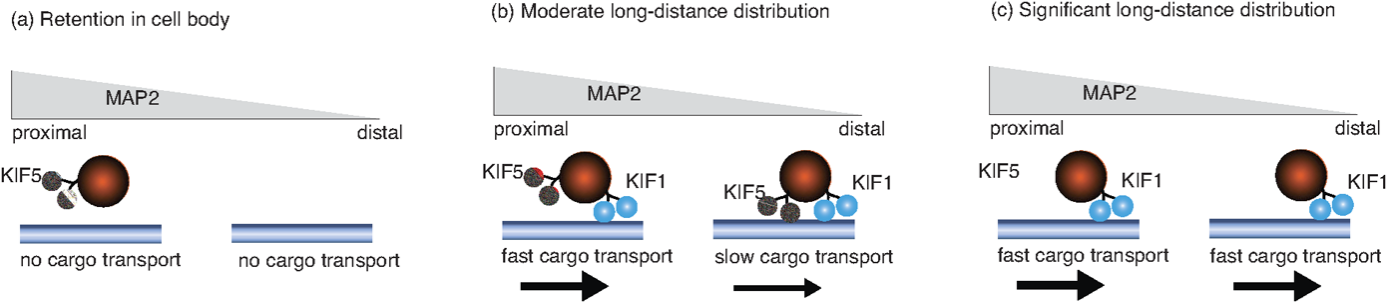
Schematic illustration of how MAP2 regulation of kinesin motor activities leads to cargo sorting and trafficking in axons. (Redrawn from [68])

Interestingly, there is recent evidence that the selective transport of cargo into the axon depends on the localized restriction of MAP2 to the proximal axon [67]. It is known that in both mammalian and Drosophila axons, secretory vesicles are trafficked by the cooperative action of two types of kinesin motors, KIF5 and KIF1 motors. Experimental studies of their motility indicate that MAP2 directly inhibits KIF5 motor activity and that axonal cargo entry and distribution depend on the balanced activities between KIF5 and KIF1 bound to the same cargo. That is, cargoes bound to the dominant motor KIF5 are unable to enter the axon, whereas those bound to motors that are not influenced by MAP2 are able to quickly enter the axon and move to the distal terminals. Moreover, cargoes bound to both KIF1 and KIF5 will enter the axon, but their axonal distribution will be affected by the reactivation of KIF5 past the proximal axon as the inhibition by MAP2 wears off, which slows down the transport; see Fig. 17.

### Synaptic Democracy

A number of recent experimental studies of intracellular transport in axons of *C. elegans* and *Drosophila* have shown that (i) motor-driven vesicular cargo exhibits “stop and go” behavior, in which periods of ballistic anterograde or retrograde transport are interspersed by long pauses at presynaptic sites, and (ii) the capture of vesicles by synapses during the pauses is reversible in the sense that the aggregation of vesicles can be inhibited by signaling molecules resulting in dissociation from the target [96, 148]. It has thus been hypothesized that the combination of inefficient capture at presynaptic sites and the back-and-forth motion of motor-cargo complexes between proximal and distal ends of the axon facilitates a more uniform distribution of resources, that is, greater “synaptic democracy” [96].

The idea of synaptic democracy has previously arisen within the context of equalizing synaptic efficacies, that is, ensuring that synapses have the same potential for affecting the postsynaptic response regardless of their locations along the dendritic tree [71, 126]. An analogous issue arises within the context intracellular transport, since vesicles are injected from the soma (anterograde transport) so that one might expect synapses proximal to the soma to be preferentially supplied with resources. In principle, this could be resolved by routing cargo to specific synaptic targets, but there is no known form of molecular address system that could support such a mechanism, particularly in light of the dynamically changing distribution of synapses. From a mathematical perspective, the issue of synaptic democracy reflects a fundamental property shared by the one-dimensional advection–diffusion equation used to model active transport and the cable equation used to model ionic current flow, namely, they generate an exponentially decaying steady-state solution in response to a localized source of active particles or current.

**Fig. 18:**
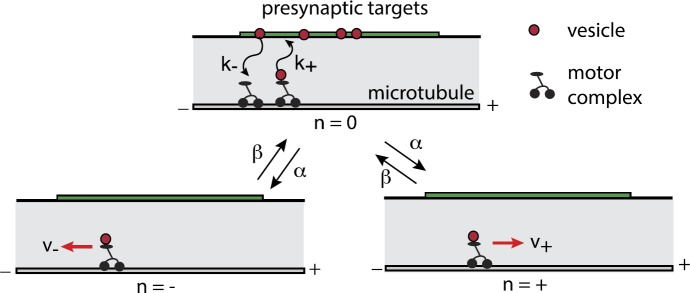
Three-state model of the bidirectional transport of a single motor-cargo complex. The particle switches between an anterograde state ($n=+$) of speed $v_{+}$, a stationary or slowly diffusing state ($n=0$), and a retrograde state ($n=-$) of speed $v_{-}$. The motor-complex can only deliver a vesicle to a presynaptic target in the state $n=0$

The hypothesized mechanism of synaptic democracy that combines bidirectional transport with reversible delivery of cargo to synaptic targets has recently been investigated in a series of modeling studies [13, 16, 20, 78]. Consider a simple three-state transport model of a single motor-complex moving on a semiinfinite 1D track as shown in Fig. 18. The motor complex is taken to be in one of three motile states labeled by $n=0,\pm$: stationary or slowly diffusing with diffusivity $D_{0}$ ($n=0$), moving to the right (anterograde) with speed $v_{+}$ ($n=+$), or moving to the left (retrograde) with speed $-v_{-}$ ($n=-$); transitions between the three states are governed by a discrete Markov process. In addition, the motor complex can carry a single vesicle, which is reversibly exchanged with membrane-bound synaptic targets when in the state $n=0$. Let $p_{n}(x,t) $ denote the probability density that at time *t* the complex is at position *x*, $x\in(0,\infty)$, is in motile state *j*, and a vesicle is not bound to the complex. Similarly, let $\widehat {p}_{n}(x,t)$ be the corresponding probability density when a vesicle is bound. We allow for the possibility that the velocities and diffusivity are different for the bound state by taking $v_{\pm}\rightarrow \widehat{v}_{\pm}$ and $D_{0}\rightarrow\widehat{D}_{0}$. The evolution of the probability density is described by the following system of partial differential equations:

5.16a $$\begin{aligned} \frac{\partial p_{\pm}}{\partial t} =& \mp v_{\pm}\frac{\partial p_{\pm}}{\partial x} -\beta p_{\pm}+\alpha p_{0}, \end{aligned}$$

5.16b $$\begin{aligned} \frac{\partial\widehat{p}_{\pm}}{\partial t} =& \mp\widehat {v}_{\pm}\frac{\partial\widehat{p}_{\pm}}{\partial x} -\beta \widehat{p}_{\pm}+\alpha\widehat{p}_{0}, \end{aligned}$$

5.16c $$\begin{aligned} \frac{\partial p_{0}}{\partial t} =&D_{0}\frac{\partial ^{2}p_{0}}{\partial x^{2}}+ \beta p_{+}+ \beta p_{-} -2\alpha p_{0} +k_{+} \widehat{p}_{0}-k_{-}cp_{0}, \end{aligned}$$

5.16d $$\begin{aligned} \frac{\partial\widehat{p}_{0}}{\partial t} =&\widehat{D}_{0}\frac {\partial^{2} \widehat{p}_{0}}{\partial x^{2}}+ \beta \widehat{p}_{+}+ \beta\widehat{p}_{-} -2\alpha \widehat{p}_{0} -k_{+} \widehat {p}_{0}+k_{-}cp_{0}. \end{aligned}$$

Here *α*, *β* are the transition rates between the slowly diffusing and ballistic states. We also assume that there is a uniform distribution *c* of presynaptic targets along the axon, which can exchange vesicles with the motor-complex at the rates $k_{\pm}$.

Now suppose that the transition rates *α*, *β* are fast compared to the exchange rates $k_{\pm}$ and the effective displacement rates of the complex on a fundamental microscopic length-scale such as the size of a synaptic target ($l\sim1~\mu\mbox{m}$). Following Sect. 2.2, we can then use a QSS diffusion approximation to derive an advection–diffusion equation for the total probability densities

5.17 $$ p(x,t)=\sum_{n=0,\pm} p_{n}(x,t) ,\qquad \widehat{p}(x,t)=\sum_{n=0,\pm} \widehat{p}_{n}(x,t). $$

That is, we obtain the equations

5.18a $$ \frac{\partial p}{\partial t}=- v\frac{\partial p}{\partial x}+D \frac{\partial^{2} p}{\partial x^{2}}+k_{+} \widehat{p}-k_{-}cp $$

and

5.18b $$ \frac{\partial\widehat{p}}{\partial t}=- \widehat{v}\frac{\partial \widehat{p}}{\partial x}+\widehat{D} \frac{\partial^{2} \widehat {p}}{\partial x^{2}}-k_{+}\widehat{p}+k_{-}cp, $$

where

$$\begin{aligned} v =&(v_{+}-v_{-})\rho_{+},\qquad \widehat{v}=( \widehat{v}_{+}-\widehat {v}_{-})\rho_{+}, \\ D =&D_{0}\rho_{0}+\frac{\alpha}{\beta(2\alpha+\beta)} \bigl({(v_{+}-v)^{2}}+{(v_{-}+v)^{2}} \bigr), \end{aligned}$$

and

$$ \widehat{D} =\widehat{D}_{0}\rho_{0}+\frac{\alpha}{\beta(2\alpha +\beta)} \bigl({(\widehat{v}_{+}-\widehat{v})^{2}}+{(\widehat {v}_{-}+\widehat{v})^{2}} \bigr). $$

Here

5.19 $$ \rho_{0}=\frac{\beta}{2\alpha+\beta},\qquad \rho_{\pm}= \frac {\alpha}{2\alpha+\beta} $$

are the stationary probabilities of the three-state Markov process describing transitions between the motile states $n=0$ and $n=\pm$, respectively. We have also absorbed a factor $\rho_{0}$ into $k_{\pm}$.

To investigate how the above form of intracellular transport can lead to synaptic democracy, we consider a population of identical, noninteracting motor complexes. Let $u(x,t)$ and $\widehat{u}(x,t)$ denote the density of motor-complexes without and with an attached vesicle, respectively. From the reduced equations (5.18a)–(5.18b) we have

5.20a $$ \frac{\partial u}{\partial t}=- v\frac{\partial u}{\partial x}+D \frac{\partial^{2} u}{\partial x^{2}}-\gamma u+k_{+} \widehat{u}-k_{-}cu $$

and

5.20b $$ \frac{\partial\widehat{u}}{\partial t}=- \widehat{v}\frac{\partial \widehat{u}}{\partial x}+\widehat{D} \frac{\partial^{2} \widehat {u}}{\partial x^{2}}-\widehat{\gamma} u-k_{+}\widehat{u}+k_{-}cu $$

for $x>0$. In the population model, we have included the degradation terms *γu* and *γû*, which account for the fact that motor-complexes may dysfunction and no longer exchange cargo with synaptic targets. Equations (5.20a)–(5.20b) are supplemented by the following boundary conditions at $x=0$:

$$J\bigl(u(0,t)\bigr)= J_{0}, \qquad J\bigl(\widehat{u}(0,t)\bigr)= \widehat{J}_{0}, $$

where $J(u)= -D\partial_{x} u+ vu$ etc. That is, motor-complexes without and with cargo are injected at the somatic end $x=0$ at constant rates $J_{0}$, and $\widehat{J}_{0}$, respectively. It is important to emphasize that the injected motor complexes are not necessarily newly synthesized from the cell body. For it has been found experimentally that motor-complexes recycle between the distal and somatic ends of the soma [96, 148]. In the case of a finite axon, we could model recycling by imposing an absorbing boundary condition at the distal end and reinjecting the distal flux into the somatic end. Since most of these complexes would be without a vesicle, this would mainly contribute to $J_{0}$. Moreover, if the axon is much longer than the range of vesicular delivery necessary to supply en passant synapses, then the effects of the absorbing boundary can be ignored, and we can treat the axon as semiinfinite. Finally, at the population level, the concentration of vesicles within the presynaptic targets is no longer constant, that is, $c=c(x,t)$ with

5.21 $$ \frac{\partial c}{\partial t}= k_{+} u(x,t)-k_{-}c(x,t)\widehat {u}(x,t)-\gamma_{c}c(x,t). $$

We have also allowed for the possibility that synaptic vesicles degrade at a rate $\gamma_{c}$.

Let us begin by considering the case $k_{-}>0$ (reversible delivery) and $\gamma_{c}=0$ (no vesicular degeneration); the distribution *c* of presynaptic vesicles will remain bounded, provided that $J_{0}>0$. Equation (5.21) implies that, at steady state,

5.22 $$ c(x)=\frac{k_{+}\widehat{u}(x)}{k_{-}u(x)}. $$

Then substituting equation (5.22) into the steady-state versions of equations (5.20a)–(5.20b) gives

5.23a $$ u(x)=\frac{J_{0}\mathrm{e}^{-x/\xi}}{ D/\xi+v},\quad \xi=\frac {2D}{-v+ \sqrt{v^{2}+4D \gamma}}, $$

and

5.23b $$ \widehat{u}(x)=\frac{\widehat{J}_{0}\mathrm{e}^{-x/\widehat{\xi}}}{ \widehat{D}/\widehat{\xi}+\widehat{v}},\quad \widehat{\xi}=\frac {2\widehat{D}}{-\widehat{v}+ \sqrt{\widehat{v}^{2}+4\widehat{D} \gamma}}. $$

Combining with equation (5.22) then yields the following result for the steady-state density of synaptic vesicles:

5.24 $$ c(x)=\frac{k_{+}}{k_{-}}\frac{\widehat{J}_{0}}{J_{0}}\frac{ D/\xi+v}{ \widehat{D}/\widehat{\xi}+\widehat{v}} \mathrm{e}^{-\varGamma x}, $$

where

$$\varGamma= \widehat{\xi}^{-1}-\xi^{-1}. $$

In particular, if the transport properties of the motor-complex are independent of whether or not a vesicle is bound ($v=\widehat{v}$, $D=\widehat{D}$), then $\xi=\widehat{\xi}$, and we have a uniform vesicle distribution

$$c(x)=\bar{c}:=\frac{k_{+}}{k_{-}}\frac{\widehat{J}_{0}}{J_{0}}. $$

**Fig. 19:**
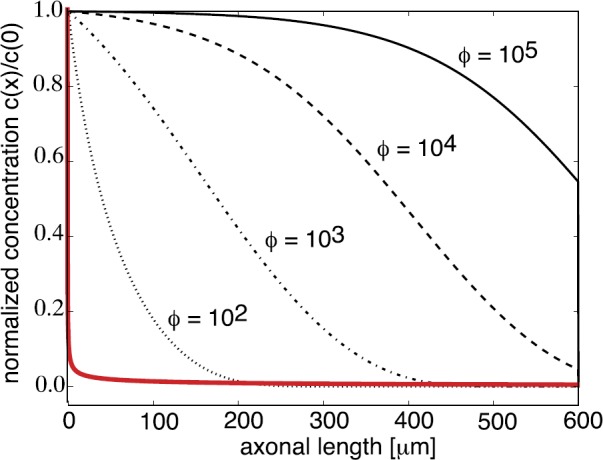
Numerical solutions for steady-state vesicle concentration as a function of axonal distance for different values of $\phi=k_{-}/\gamma _{c}$ and $J_{0} = 1.5$. (Adapted from [20].) For comparison, the corresponding concentration profile when $J_{0}=0$ (which is insensitive to *ϕ*) is shown by the thick line (red line in color online). We have also set $\gamma= 10^{-2}~\mbox{s}^{-1} $, $\widehat{J}=1.5$, $k_{+} = 0.5~\mbox{s}^{-1}$, $k_{-}=1.0~\mu\mbox{ms}^{-1}$, $v= \widehat{v} = 1~\mu\mbox{ms}^{-1}$ and $D =\widehat {D}= 0.1~\mu\mbox{ms}^{-2}$

To further explore the ability of this model to produce a democratic cargo distribution, equation (5.20a)–(5.20b) can be solved numerically for a range of parameter values. Following [20], suppose that $\gamma_{c}$ is small (relative to $k_{\pm}$) but nonzero and consider how the normalized distribution $c(x)/c(0)$ varies with $\phi\equiv k_{-}/\gamma_{c}$, which determines the proportion of vesicles that are recycled into the system after leaving the targets. Figure 19 displays the normalized concentration profiles for a variety of $k_{-}/\gamma_{c}$ values with either $J_{0}=\widehat{J}_{0}$ or $J_{0}=0$. (The domain size is taken to be sufficiently large to avoid boundary effects.) It can be seen that when $J_{0}>0$, the length scale over which nonexponential decay occurs is an increasing function of $k_{-}/\gamma_{c}$, whereas when $J_{0}=0$, the model fails to distribute cargo across a substantial region of the axon. Hence an additional component of a delivery mechanism that includes recapture is a source of motors which are able to receive vesicles. It should be emphasized that this does not require additional motors to be synthesized in the soma; instead, motors may return to the beginning of the axon after delivering their cargo. From the perspective of synaptic democracy it seems desirable to maximize $k_{-}$; however, increasing the recapture rate decreases the efficiency of the delivery mechanism and can result in a overall loss of vesicles due to motor degradation.

This mechanism for synaptic democracy appears to be quite robust. For example, it can be extended to the case where each motor carries a vesicular aggregate rather than a single vesicle, assuming that only one vesicle can be exchanged with a target at any one time [13]. The effects of reversible vesicular delivery also persist when exclusion effects between between motor-cargo complexes are taken into account [16] and when higher-dimensional cell geometries are considered [78].

## Phase Reduction of Stochastic Hybrid Oscillators

In Sects. 2.3 and 3 we assumed that, in the adiabatic limit $\varepsilon \rightarrow0$, the resulting deterministic dynamical system exhibited bistability, and we explored how random switching of the associated PDMP for small *ε* can lead to noise-induced transitions between metastable states. In this section, we assume that the deterministic system supports a stable limit cycle so that the corresponding PDMP acts as a stochastic limit cycle oscillator, at least in the weak noise regime. There is an enormous literature on the analysis of stochastic limit cycle oscillators for SDEs (for recent surveys, see the reviews [3, 47, 105]). On the other hand, as far as we are aware, there has been very little numerical or analytical work on limit cycle oscillations in PDMPs. A few notable exceptions are [21, 27, 52, 89, 137]. One possible approach would be to carry out a QSS diffusion approximation of the PDMP along the lines of Sect. 2.2 and then use stochastic phase reduction methods developed for SDEs. In this section, we review an alternative, variational method that deals directly with the PDMP [21], thus avoiding additional errors arising from the diffusion approximation. Another major advantage of the variational method is that it allows us to obtain rigorous exponential bounds on the expected time to escape from a neighborhood of the limit cycle [21, 22].

Let us first briefly consider SDEs. Suppose that a deterministic smooth dynamical system $\dot{x}=F(x)$, $x \in \mathbb {R}^{d}$, supports a limit cycle $x(t)=\varPhi(\theta(t))$ of period $\varDelta _{0}$, where $\theta(t)$ is a uniformly rotating phase, $\dot{\theta}=\omega_{0}$, and $\omega_{0}=2\pi/\varDelta _{0}$. The phase is neutrally stable with respect to perturbations along the limit cycle; this reflects invariance of an autonomous dynamical system with respect to time shifts. Now suppose that the dynamical system is perturbed by weak Gaussian noise such that $dX=F(X)\,dt+\sqrt{2\varepsilon } G(X) \, dW(t)$, where $W(t)$ is a *d*-dimensional vector of independent Wiener processes. If the noise amplitude *ε* is sufficiently small relative to the rate of attraction to the limit cycle, then deviations transverse to the limit cycle are also small (up to some exponentially large stopping time). This suggests that the definition of a phase variable persists in the stochastic setting, and we can derive a stochastic phase equation by decomposing the solution to the SDE according to

**Fig. 20:**
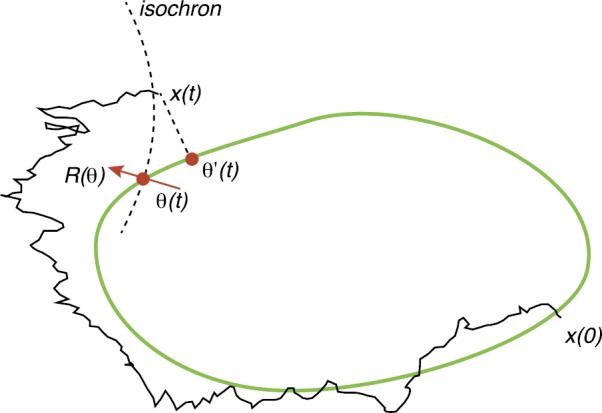
Different choices of amplitude-phase decomposition. Two possibilities are orthogonal projection with phase $\theta'(t)$ and isochronal projection with phase $\theta(t)$. In the latter case, the response to perturbations depends on the phase response curve ${R}(\theta)$, which is normal to the isochron at the point of intersection with the limit cycle

6.1 $$ X(t)=\varPhi\bigl(\beta(t)\bigr)+\sqrt{ \varepsilon }v(t) $$

with $\beta(t)$ and $v(t)$ corresponding to the phase and amplitude components, respectively. However, there is not a unique way to define the phase *β*, which reflects the fact that there are different ways of projecting the exact solution onto the limit cycle [7, 21, 65, 87, 147]; see Fig. 20. One well-known approach is to use the method of isochrons [47, 62, 106, 135, 136, 149]. Recently, a variational method for carrying out the amplitude-phase decomposition for SDEs has been developed, which yields exact SDEs for the amplitude and phase [22]. Within the variational framework, different choices of phase correspond to different choices of the inner product space $\mathbb {R}^{d}$. By taking an appropriately weighted Euclidean norm the minimization scheme determined the phase by projecting the full solution on to the limit cycle using Floquet vectors. Hence, in a neighborhood of the limit cycle the phase variable coincided with the isochronal phase [7]. This had the advantage that the amplitude and phase decoupled to leading order. In addition, the exact amplitude and phase equations could be used to derive strong exponential bounds on the growth of transverse fluctuations. It turns out that an analogous variational method can be applied to PDMPs [21], which will be outlined in the remainder of this section.

Suppose that the deterministic dynamical system (2.8), obtained in the adiabatic limit $\varepsilon \rightarrow0$, supports a stable periodic solution $x=\varPhi(\omega_{0} t)$ with $\varPhi(\omega _{0}t)=\varPhi(\omega_{0}[t+\varDelta _{0}])$, where $\omega_{0}=2\pi/\varDelta _{0}$ is the natural frequency of the oscillator. In the state space of the continuous variable, the solution is an isolated attractive trajectory called a limit cycle. The dynamics on the limit cycle can be described by a uniformly rotating phase such that

6.2 $$ \frac{d\theta}{dt}=\omega_{0}, $$

and $x={\varPhi}(\theta(t))$ with a 2*π*-periodic function *Φ*. Note that the phase is neutrally stable with respect to perturbations along the limit cycle—this reflects invariance of an autonomous dynamical system with respect to time shifts. By definition, *Φ* must satisfy the equation

6.3 $$ \omega_{0} \frac{d\varPhi}{d\theta} = \overline{F}\bigl( \varPhi(\theta)\bigr). $$

Differentiating both sides with respect to *θ* gives

6.4 $$ \frac{d}{d\theta} \biggl(\frac{d\varPhi}{d\theta} \biggr)=\omega _{0}^{-1}\overline{J}( \theta)\cdot\frac{d\varPhi}{d\theta}, $$

where *J̅* is the 2*π*-periodic Jacobian matrix

6.5 $$ \overline{J}_{jk}(\theta)\equiv \frac{\partial\overline {F}_{j}}{\partial x_{k}} \bigg|_{x=\varPhi(\theta)}. $$

**Fig. 21:**
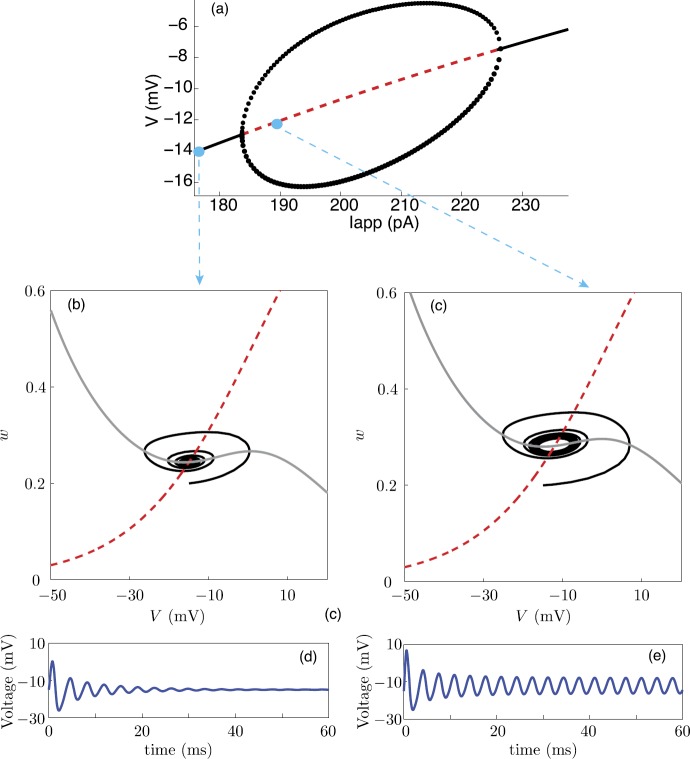
ML model for subthreshold oscillations. (Adapted from [27].) (**a**) Bifurcation diagram of the deterministic ML model. As $I_{\mathrm{app}}$ is increased, the system undergoes a supercritical Hopf bifurcation (H) at $I_{\mathrm{app}}^{*}=183$ pA, which leads to the generation of stable oscillations. The maximum and minimum values of oscillations are plotted as black (solid) curves. Oscillations disappear via another supercritical Hopf bifurcation. (**b**), (**c**) Phase plane diagrams of the deterministic model for (b) $I_{\mathrm{app}}=170~\mbox{pA}$ (below the Hopf bifurcation point) and (c) $I_{\mathrm{app}}=190~\mbox{pA}$ (above the Hopf bifurcation point). The red (dashed) curve is the w-nullcline and the solid (gray) curve represents the v-nullcline. (**d**), (**e**) Corresponding voltage time courses. In contrast to Sect. 3.1, we now take $\alpha_{\mathrm{K}}=\beta_{\mathrm{K}}\mathrm{e}^{2[v-v_{\mathrm{K},1}]/v_{\mathrm{K},2}}$. *Sodium parameters*: $g_{\mathrm{Na}}= 4.4~\mbox{mS}$, $V_{\mathrm{Na}}= 55~\mbox{mV}$, $\beta_{\mathrm{Na}} = 100~\mbox{ms}^{-1}$, $v_{\mathrm{Na},1}= -1.2~\mbox{mV}$, $v_{\mathrm{Na},2}=18~\mbox{mV}$. *Leak parameters*: $g_{\mathrm{L}}=2.2~\mbox{mS}$, $V_{\mathrm{L}}= -60~\mbox{mV}$. *Potassium parameters*: $g_{\mathrm{K}}=8~\mbox{mS}$, $V_{\mathrm{K}}=-84~\mbox{mV}$, $\beta_{\mathrm{K}}= 0.35~\mbox{ms}^{-1}$, $v_{\mathrm{K},1}= 2~\mbox{mV}$, $v_{\mathrm{K},2}= 30~\mbox{mV}$. Also $C_{m}=1~\mbox{mF}$

One concrete example of a PDMP that supports a limit cycle oscillation in the fast switching limit is a version of the stochastic Morris–Lecar model that has been applied to sodium-based subthreshold oscillations [27, 145]; the corresponding deterministic model is given by equations (3.5). Numerical solutions of the latter are shown in Fig. 21.

The isochronal phase map has been the most popular means of decomposing the phase of stochastic oscillators evolving according to an SDE (and also studying their synchronization) [3, 47, 105]. Let $\mathscr {U}$ be the neighborhood of the limit cycle consisting of all points that eventually converge to the limit cycle under the deterministic dynamics of (2.8). The isochronal phase map $\varTheta: \mathscr {U} \to\mathbb{S}^{1}$ is defined to be the phase that a point converges to. That is, $\varTheta (y)$ is the unique *α* such that if $x(0) = y$ and

6.6 $$ \frac{dx}{dt} = \overline{F} \bigl(x(t) \bigr), $$

then $\lim_{t\to\infty} \Vert x(t) - \varPhi(\alpha+t\omega_{0}) \Vert = 0$. Hence, in a neighborhood of the deterministic limit cycle, we have

$$ {\omega}_{0} = \frac{d\varTheta(x)}{dt}=\nabla\varTheta(x)\cdot \frac {dx}{dt} =\nabla\varTheta(x)\cdot\overline{F}(x) . $$

Now let $\alpha= \varTheta(x)$ for $x(t)$ evolving according to the PDMP (2.1), assuming for the moment that $x(t)\in \mathscr {U}$. From the chain rule of calculus it follows that the isochronal phase evolves according to the piecewise deterministic dynamics

6.7 $$ \frac{d\alpha}{dt}= \bigl\langle \nabla\varTheta(x),F_{n}(x) \bigr\rangle , $$

with switching events occurring at the same times $\lbrace t_{k} \rbrace$ as $x(t)$. The gradient of the isochronal phase,

6.8 $$ R(\theta)=\nabla\varTheta(x)|_{x=\varPhi(\theta)}, $$

is known as the infinitesimal phase resetting curve. It can be shown that $R(\theta)$ satisfies the adjoint equation [48]

6.9 $$ \omega_{0}\frac{dR(\theta)}{d\theta}=-\overline{J}( \theta)^{\top }\cdot R(\theta) $$

under the normalization condition

6.10 $$ R(\theta)\cdot\frac{d\varPhi(\theta)}{d\theta}=1. $$

As it stands, equation (6.7) is not a closed equation for the isochronal phase, since the right-hand side depends on the full set of variables $x(t)$.

### Floquet Decomposition

Suppose that we fix a particular realization $\sigma_{T}$ of the Markov chain up to some time *T*, $\sigma_{T}=\{N(t),0\leq t \leq T\}$. Suppose that there is a finite sequence of jump times $\{t_{1},\ldots, t_{r}\}$ within the time interval $(0,T)$ and let $n_{j}$ be the corresponding discrete state in the interval $(t_{j},t_{j+1})$ with $t_{0}=0$. Introduce the set

$${\mathscr {T}}=[0,T]\bigm\backslash \bigcup_{j=1}^{r} \{t_{j}\}. $$

We wish to decompose the piecewise deterministic solution $x_{t}$ to the PDMP (2.1) for $t\in{ \mathscr {T}}$ into two components as in equation (6.1):

6.11 $$ x_{t}=\varPhi(\beta_{t})+\sqrt{ \varepsilon }v_{t} $$

with $\beta_{t}$ and $v_{t}$ corresponding to the phase and amplitude components, respectively. The phase $\beta_{t}$ and amplitude $v_{t}$ evolve according to a PDMP, involving the vector field $F_{n_{j}}$ in the time intervals $(t_{j},t_{j+1})$, analogous to $x_{t}$; see Fig. 1. (It is notationally convenient to switch from $x(t)$ to $x_{t}$ etc.) However, such a decomposition is not unique unless we impose an additional mathematical constraint. We will adapt a variational principle recently introduced to analyze the dynamics of limit cycles with Gaussian noise [21]. To construct the variational principle, we first introduce an appropriate weighted norm on $\mathbb {R}^{d}$, based on a Floquet decomposition.

For any $0 \leq t$, define $\varPi(t) \in \mathbb {R}^{d\times d}$ to be the fundamental matrix for the following ODE:

6.12 $$ \frac{d z}{dt} = A(t)z, $$

where $A(t)=\overline{J}(\omega_{0} t)$. That is, $\varPi(t):= ( z_{1}(t) | z_{2}(t) | \cdots|z_{d}(t) )$, where $z_{i}(t)$ satisfies (6.12), and $\lbrace z_{i}(0) \rbrace_{i=1}^{d}$ is an orthogonal basis for $\mathbb {R}^{d}$. Floquet theory states that there exists a diagonal matrix $\mathscr {S}=\operatorname{diag}(\nu_{1},\ldots,\nu_{d})$ whose diagonal entries are the Floquet characteristic exponents such that

6.13 $$ \varPi ( t ) = P (\omega_{0} t )\exp (t\mathscr {S} )P^{-1}(0), $$

with $P(\theta)$ a 2*π*-periodic matrix whose first column is proportional to $\varPhi'(\omega_{0}t)$, and $\nu_{1} = 0$. That is, $P(\theta)^{-1}\varPhi'(\theta) =c_{0}\mathbf{e}$ with $\mathbf{e}_{j}=\delta _{1,j}$ and $c_{0}$ an arbitrary constant; we set $c_{0}=1$ for convenience. To simplify the following notation, we will assume throughout this paper that the Floquet multipliers are real and hence $P(\theta)$ is a real matrix. We can readily generalize these results to the case that $\mathscr {S}$ is complex. The limit cycle is taken to be stable, meaning that, for a constant $b > 0$ and all $2\leq i \leq d$, we have $\nu_{i} \leq- b$. Furthermore, $P^{-1}(\theta)$ exists for all *θ*, since $\varPi ^{-1}(t)$ exists for all *t*.

The above Floquet decomposition motivates the following weighted inner product: For any $\theta\in \mathbb {R}$, we denote the standard Euclidean dot product on $\mathbb{R}^{d}$ by $\langle\cdot, \cdot\rangle$,

$$\langle x,y \rangle_{\theta}= \bigl\langle P^{-1}(\theta )x,P^{-1}(\theta)y \bigr\rangle , $$

and $\Vert x \Vert _{\theta}= \sqrt{\langle x,x\rangle_{\theta }}$. In the case of SDEs, it has been shown that this choice of weighting yields a leading order separation of the phase from the amplitude and facilitates strong bounds on the growth of $v_{t}$ [21]. The former is a consequence of the fact that the matrix $P^{-1}(\theta )$ generates a coordination transformation in which the phase in a neighborhood of the limit cycle coincides with the asymptotic phase defined using isochrons (see also [7]). In particular, we can show that the PRC $R(\theta)$ is related to the tangent vector $\varPhi'(\theta)$ according to [21]

6.14 $$ P^{\top}(\theta){R}(\theta)=\mathfrak {M}_{0}^{-1}P^{-1}( \theta)\varPhi'(\theta), $$

where

6.15 $$ \mathfrak {M}_{0}:= \bigl\Vert \varPhi'(\theta) \bigr\Vert ^{2}_{\theta}= \bigl\Vert P^{-1}(\theta)\varPhi '(\theta) \bigr\Vert ^{2} =1. $$

### Defining the Piecewise Deterministic Phase Using a Variational Principle

We can now state the variational principle for the stochastic phase $\beta_{t}$ [21]. First, we consider a variational problem for an arbitrary prescribed function $\theta_{t}$ (not to be confused with the phase on the limit cycle), which specifies the weighted Euclidean norm. Given $\theta_{t}$, we determine $\beta_{t}$ for $t \in{ \mathscr {T}}$ by requiring $\beta_{t}=\varphi_{t}(\theta_{t})$, where $\varphi_{t}(\theta_{t})$ is a local minimum of the following variational problem:

6.16 $$ \underset{\varphi\in{ \mathscr {N}}\bigl(\varphi_{t}( \theta_{t})\bigr)}{\inf}\bigl\Vert x_{t}-\varPhi(\varphi) \bigr\Vert _{\theta_{t}} = \bigl\Vert x_{t}-\varPhi\bigl( \varphi_{t}(\theta_{t})\bigr) \bigr\Vert _{\theta_{t}} , \quad t \in{ \mathscr {T}}, $$

with ${\mathscr {N}} (\varphi_{t}(\theta_{t}) )$ denoting a sufficiently small neighborhood of $\varphi_{t} (\theta_{t} )$. The minimization scheme is based on the orthogonal projection of the solution onto the limit cycle with respect to the weighted Euclidean norm. We will derive an exact SDE for $\beta_{t}$ (up to some stopping time) by considering the first derivative

6.17 $$ \mathscr {G}_{0}(z,\varphi,\theta):=\frac{\partial}{\partial\varphi} \bigl\Vert z-\varPhi(\varphi) \bigr\Vert ^{2}_{\theta} =-2 \bigl\langle z-\varPhi(\varphi),\varPhi '(\varphi) \bigr\rangle _{\theta}. $$

At the minimum,

6.18 $$ \mathscr {G}_{0}(x_{t},\beta_{t}, \theta_{t})=0. $$

We then determine $\theta_{t}$ (and hence $\beta_{t}$) self-consistently by imposing the condition $\theta_{t} = \varphi_{t}(\theta_{t})=\beta_{t}$. It follows that the stochastic phase $\beta_{t}$ satisfies the implicit equation

6.19 $$ \mathscr {G}(x_{t},\beta_{t}):= \mathscr {G}_{0}(x_{t}, \beta_{t},\beta_{t})=0. $$

It will be seen that, up to a stopping time *τ*, there exists a unique continuous solution to this equation. Define $\mathfrak {M}(z,\varphi) \in \mathbb {R}$ as

6.20 $$\begin{aligned} \mathfrak {M}(z,\varphi)&:=\frac{1}{2}\frac{\partial \mathscr {G}(z,\varphi)}{\partial \varphi} \\ &=\frac{1}{2}\frac{\partial \mathscr {G}_{0}(z,\varphi,\theta )}{\partial\varphi} \bigg|_{\theta=\varphi}+\frac{1}{2} \frac{\partial \mathscr {G}_{0}(z,\varphi,\theta)}{\partial\theta} \bigg|_{\theta=\varphi } \\ &=\mathfrak {M}_{0} - \bigl\langle z-\varPhi(\varphi), \varPhi''( \varphi) \bigr\rangle _{\varphi} \\ &\quad {} - \biggl\langle z-\varPhi(\varphi),\frac{d}{d\varphi} \bigl\{ \bigl[{P( \varphi)P^{\top}(\varphi)} \bigr]^{{-1}} \bigr\} \varPhi '(\varphi)\biggr\rangle . \end{aligned}$$

Assume that initially $\mathfrak {M}(u_{0},\beta_{0})>0$. We then seek a PDMP for $\beta_{t}$ that holds for all times less than the stopping time

6.21 $$ \tau=\inf\bigl\{ s\geq0: \mathfrak {M}(u_{s},\beta_{s})=0 \bigr\} . $$

The implicit function theorem guarantees that a unique continuous $\beta_{t}$ exists until this time.

To derive the PDMP for $\beta_{t}$, we consider the equation

6.22 $$ \frac{d \mathscr {G}_{t} }{dt} \equiv\frac{d \mathscr {G}(x_{t},\beta_{t})}{dt}=0,\quad t \in{ \mathscr {T}}, $$

with $x_{t}$ evolving according to the PDMP (2.1). From the definition of $\mathscr {G}(x_{t},\beta_{t})$ it follows that

6.23 $$ 0 =-2 \biggl\langle \frac{dx_{t}}{dt},\varPhi'(\beta_{t}) \biggr\rangle _{\beta_{t}} +\frac{\partial \mathscr {G}_{t}}{\partial\varphi} \bigg|_{\varphi=\beta_{t}} \frac{d\beta_{t}}{dt},\quad t \in{ \mathscr {T}}. $$

Rearranging, we find that the phase $\beta_{t}$ evolves according to the exact, but implicit, PDMP

6.24 $$ \frac{d\beta_{t}}{dt}={\mathfrak {M}}(x_{t},\beta_{t})^{-1} \bigl\langle F_{n}(x_{t}),\varPhi'( \beta_{t}) \bigr\rangle _{\beta_{t}} , $$

with $n=n_{j}$ for $t \in(t_{j},t_{j+1})$. Finally, recalling that the amplitude term $v_{t}$ satisfies $\sqrt{ \varepsilon }v_{t}=x_{t}-\varPhi_{\beta_{t}}$, we have

6.25 $$\begin{aligned} \sqrt{ \varepsilon }\frac{dv_{t}}{dt}&=\frac{dx_{t}}{dt}-\varPhi'( \beta _{t})\frac{d\beta_{t}}{dt} \\ &=F_{n}(x_{t})- \mathfrak{M}(x_{t}, \beta_{t})^{-1}\varPhi'(\beta_{t}) \bigl\langle F_{n}(x_{t}),\varPhi'(\beta_{t}) \bigr\rangle _{\beta_{t}}. \end{aligned}$$

### Weak Noise Limit

Equation (6.24) is a rigorous, exact implicit equation for the phase $\beta_{t}$. We can derive an explicit equation for $\beta_{t}$ by carrying out a perturbation analysis in the weak noise limit. Let $0 <\varepsilon \ll1$ and set $x_{t}=\varPhi(\beta_{t})$ on the right-hand side of (6.24), that is, $v_{t}=0$. Writing $\beta_{t}\approx\theta _{t}$, we have the piecewise deterministic phase equation

6.26 $$\begin{aligned} \frac{d\theta_{t}}{dt}&=Z_{n}(\theta_{t}):= \mathfrak {M}_{0}^{-1} \bigl\langle F_{n}\bigl(\varPhi( \theta_{t})\bigr),\varPhi'(\theta_{t}) \bigr\rangle _{\theta} \\ &= \mathfrak {M}_{0}^{-1} \bigl\langle P(\theta_{t})^{-1}F_{n} \bigl(\varPhi(\theta _{t})\bigr),P^{-1}(\theta_{t}) \varPhi'(\theta_{t}) \bigr\rangle \\ &= \mathfrak {M}_{0}^{-1} \bigl\langle F_{n}\bigl(\varPhi( \theta_{t})\bigr),\bigl(P(\theta_{t})P(\theta _{t})^{\top}\bigr)^{-1}\varPhi'( \theta_{t}) \bigr\rangle \\ &= \bigl\langle F_{n}\bigl(\varPhi(\theta_{t})\bigr),R( \theta_{t}) \bigr\rangle ,\quad n=n_{j} \mbox{ for } t \in(t_{j},t_{j+1}) \\ &=\omega_{0}+ \bigl\langle F_{n}\bigl(\varPhi( \theta_{t})\bigr)-\overline{F}\bigl(\varPhi (\theta_{t})\bigr),R( \theta_{t}) \bigr\rangle \end{aligned}$$

after using ${\mathfrak {M}}(\varPhi(\theta),\theta)=\mathfrak {M}_{0}$ and equation (6.14). The last line follows from the observation

$$\begin{aligned} \bigl\langle \overline{F}\bigl(\varPhi(\theta)\bigr),R(\theta) \bigr\rangle &= \omega_{0} \bigl\langle \varPhi'(\theta),R(\theta) \bigr\rangle \\ &= \omega_{0} \mathfrak {M}_{0}^{-1} \bigl\Vert \varPhi'(\theta) \bigr\Vert _{\theta}^{2} = \omega_{0}. \end{aligned}$$

Hence, a phase reduction of the PDMP (2.1) yields a PDMP for the phase $\theta_{t}$. Of course, analogously to the phase reduction of SDEs, there are errors due to the fact that we have ignored $O(\varepsilon )$ terms arising from amplitude-phase coupling; see below. This leads to deviations of the phase $\theta_{t}$ from the exact variational phase $\beta_{t}$ over $O(1/\varepsilon )$ time-scales.

**Fig. 22:**
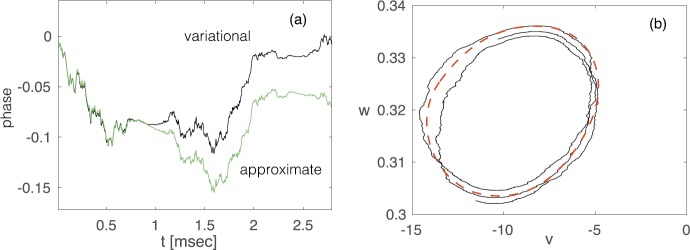
Simulation of the stochastic Morris–Lecar model for subthreshold Na^+^ oscillations with $N=10$ and $\varepsilon = 0.01$. (Adapted from Ref. [21].) Other parameter values as in Fig. 21. (**a**) Plot of the approximate phase $\theta_{t} - t\omega_{0}$ in green (with $\theta_{t}$ satisfying equation (6.26) and the exact variational phase (satisfying (6.19)) $\beta_{t} - t\omega_{0}$ in black. On the scale $[-\pi,\pi]$ the two phases are in strong agreement. However, zooming in, we can see that the phases slowly drift apart as noise accumulates. The diffusive nature of the drift in both phases can be clearly seen with the typical deviation of the phase from $\omega_{0} t$ increasing in time. (**b**) Stochastic trajectory around limit cycle (dashed curve) in the $v,w$-plane. The stable attractor of the deterministic limit cycle is quite large, which is why the system can tolerate quite substantial stochastic perturbations

In Fig. 22, we show results of numerical simulations of the stochastic ML model for $N=10$ and $\varepsilon =0.01$ with other parameters as in Fig. 21. We compare solutions of the explicit phase equation (6.26) with the exact phase defined using the variational principle; see Eq. (6.24). We also show sample trajectories for $(v,w)$. It can be seen that initially the phases are very close and then very slowly drift apart as noise accumulates. The diffusive nature of the drift in both phases can be clearly seen, with the typical deviation of the phase from $\omega_{0} t$ increasing in time.

### Decay of Amplitude Vector

If we are interested in higher-order contributions to the phase equation, then it is necessary to consider the coupling between the phase and amplitude in both the continuous dynamics and the discrete switching process. Hence, the phase equation (6.26) will only be a reasonable approximation for small *ε* if the dynamics remains within some attracting neighborhood of the limit cycle, that is, the amplitude remains small. Since the amplitude $v_{t}$ satisfies $\sqrt{ \varepsilon }v_{t}=x_{t}-\varPhi_{\beta_{t}}$, we have

6.27 $$\begin{aligned} \sqrt{ \varepsilon }\frac{dv_{t}}{dt}&=\frac{dx_{t}}{dt}-\varPhi'( \beta _{t})\frac{d\beta_{t}}{dt} \\ &=F_{n}(x_{t})- \mathfrak{M}(x_{t}, \beta_{t})^{-1}\varPhi'(\beta_{t}) \bigl\langle F_{n}(x_{t}),\varPhi'(\beta_{t}) \bigr\rangle _{\beta_{t}}. \end{aligned}$$

Now define $w_{t} = \sqrt{ \varepsilon }P(\beta_{t})^{-1}v_{t}$. Using the fact that $\dot{P}\omega_{0} = J(t)P(t)-P(t)\mathscr {S}$, we find that

6.28 $$\begin{aligned} &\frac{1}{2}\frac{d}{dt} \bigl( \Vert w_{t} \Vert ^{2} \bigr) \\ &\quad = \biggl\lbrace \langle w_{t}, \mathscr {S}w_{t} \rangle\frac{d}{dt}\beta_{t} + \biggl\langle w_{t}, P(\beta_{t})^{-1} \biggl(F_{n}(x_{t}) - J_{n} P( \beta_{t})w_{t} \frac{d\beta_{t}}{dt} \biggr) \biggr\rangle \biggr\rbrace . \end{aligned}$$

In the fast switching limit (as $\varepsilon \to0$), we can show that the dynamics of $\Vert w_{t} \Vert ^{2}$ decays to leading order [21]. That is, there exists a constant *C* such that the probability that the expected time to leave an $O(a)$ neighborhood of the limit cycle is less than *T* scales as $T\exp (-{Ca}/{\varepsilon } )$. An interesting difference between this bound and the corresponding one obtained for SDEs [22] is that in the latter the bound is of the form $T\exp (-{Cba}/{\varepsilon } )$, where *b* is the rate of decay toward the limit cycle. In other words, in the SDE case, the bound is still powerful in the large *ε* case, as long as $b\varepsilon ^{-1} \gg1$, that is, as long as the decay toward the limit cycle dominates the noise. However, this no longer holds in the PDMP case. Now, if *ε* is large, then the most likely way that the system can escape the limit cycle is that in stays in any particular state for too long without jumping, and the time that it stays in one state is not particularly affected by *b* (in most cases).

### Synchronization of Hybrid Oscillators

As we have outlined previously, it is possible to apply phase reduction techniques to PDMPs that support a limit cycle in the fast switching limit [21]. One of the important consequences of this reduction is that it provides a framework for studying the synchronization of a population of PDMP oscillators, either through direct coupling or via a common noise source. In the case of SDEs, there there have been considerable recent interest in noise-induced phase synchronization [47, 62, 106, 135, 136, 149]. This concerns the observation that a population of oscillators can be synchronized by a randomly fluctuating external input applied globally to all of the oscillators, even if there are no interactions between the oscillators. Evidence for such an effect has been found in experimental studies of neural oscillations in the olfactory bulb [59] and the synchronization of synthetic genetic oscillators [151]. A related phenomenon is the reproducibility of a dynamical system response when repetitively driven by the same fluctuating input, even though initial conditions vary across trials. One example is the spike-time reliability of single neurons [60, 98].

**Fig. 23:**
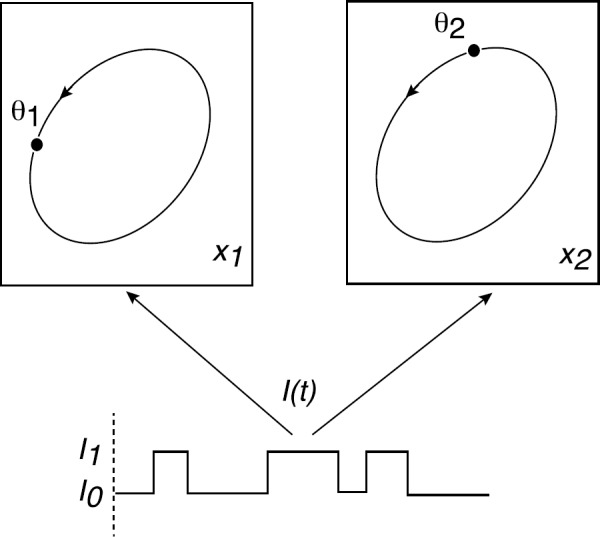
Pair of noninteracting limit cycle oscillators with phases $\theta_{j}(t)$, $j=1,2$, driven by a common switching external input $I(t)$

Most studies of noise-induced synchronization take the oscillators to be driven by common Gaussian noise. Typically, phase synchronization is established by constructing the Lyapunov exponent for the dynamics of the phase difference between a pair of oscillators and averaging with respect to the noise. If the averaged Lyapunov exponent is negative definite, then the phase difference is expected to decay to zero in the large time limit, establishing phase synchronization. However, it has also been shown that common Poisson-distributed random impulses, dichotomous or telegrapher noise, and other types of noise generally induce synchronization of limit-cycle oscillators [63, 104, 107]. Consider, in particular, the case of an additive dichotomous noise signal $I(t)$ driving a population of *M* identical noninteracting oscillators according to the system of equations $\dot{x}_{j}=F(x_{j})+I(t)$, where $x_{j}\in \mathbb {R}^{d}$ is the state of the *j*th oscillator, $j=1,\ldots,M$ [104]; see Fig. 23. Here $I(t)$ switches between two values $I_{0}$ and $I_{1}$ at random times generated by a two-state Markov chain [5]. (In the case of the classical ML model, $I(t)$ could represent a randomly switching external current.) That is, $I(t)=I_{0}(1-N(t))+I_{1}N(t)$ for $N(t)\in\{0,1\}$, with the time *T* between switching events taken to be exponentially distributed with mean switching time *τ*. Suppose that each oscillator supports a stable limit cycle for each of the two input values $I_{0}$ and $I_{1}$. It follows that the internal state of each oscillator randomly jumps between the two limit cycles. Nagai et al. [104] show that in the slow switching limit (large *τ*), the dynamics can be described by random phase maps. Moreover, if the phase maps are monotonic, then the associated Lyapunov exponent is generally negative, and phase synchronization is stable.

More generally, let $N(t) \in\varGamma\equiv\{0,\ldots,N_{0}-1\}$ denote the state of a randomly switching environment. When the environmental state is $N(t)=n$, each oscillator $x_{i}(t)$ evolves according to the piecewise deterministic differential equation $\dot{x}_{i}=F_{n}(x_{i})$ for $i=1,\ldots,M$. The additive dichotomous noise case is recovered by taking $N_{0}=2$ and $F_{n}(x)=F(x)+I_{n}$. In the slow switching limit, we can generalize the approach of Nagai et al. [104] by assuming that each of the vector fields $F_{n}(x_{i})$, $n\in\varGamma$, supports a stable limit cycle and constructing the associated random phase maps. Here we briefly discuss the fast switching regime, assuming that in the adiabatic limit $\varepsilon \rightarrow0$, the resulting deterministic system $\dot {x}_{i}=\overline{F}(x_{i})$ supports a stable limit cycle. Since there is no coupling or remaining external drive to the oscillators in this limit, their phases are uncorrelated. This then raises the issue as to whether or not phase synchronization occurs when $\varepsilon >0$.

Again, one approach would be to carry out a QSS analysis along the lines of Sect. 2.2, in which each oscillator is approximated by an SDE with a common Gaussian input. We could then adapt previous work on the phase reduction of stochastic limit cycle oscillators [62, 106, 135, 136] and thus establish that phase synchronization occurs under the diffusion approximation. However, the QSS approximation is only intended to be accurate over time-scales that are longer than $O(\varepsilon )$. Hence, it is unclear whether or not the associated Lyapunov exponent is accurate, since it is obtained from averaging the fluctuations in the noise over infinitesimally small time-scales. Therefore, it would be interesting to derive a more accurate expression for the Lyapunov exponent by working directly with an exact implicit equation for the phase dynamics such as the population analog of equation (6.24).

## Conclusion

In recent years, it has become clear that stochastic switching processes are prevalent in a wide range of biological systems. Such processes are typically modeled in terms of stochastic hybrid systems, also known as PDMPs. In this review, we provided a basic introduction to stochastic hybrid systems and illustrated the theory by considering applications to cellular neuroscience. (In a companion review paper, we focus on applications to switching gene regulatory networks [14].) We showed that although the theory of stochastic hybrid systems is underdeveloped compared to SDEs and discrete Markov processes, analogous techniques can be applied, including large deviations and WKB methods, diffusion approximations, and phase reduction methods. We end by listing several outstanding issues that are worthy of further exploration.

1. Solving the stationary version of the CK equation (2.6) for higher-dimensional stochastic hybrid systems with multiple discrete states; developing an ergodic theory of PDMPs. (See also the recent paper by Lawley et al. [4])
2. Calculating the Perron eigenvalue (Hamiltonian) of equation (2.39) for a wider range of models; currently, only a few exact solutions are known such as the ion channel model of Sect. 3; extending the theory of metastability to PDMPs with infinite Markov chains, where the Perron–Frobenius theorem does not necessarily hold.
3. Developing more detailed biophysical models of the transfer of vesicles between motor-complexes and synaptic targets; identifying local signaling mechanisms for synaptic targeting; incorporating the contribution of intracellular stores; coupling mRNA transport to long-term synaptic plasticity.
4. Solving the diffusion equation with randomly switching boundary conditions when the switching of a gate depends, for example, on the local particle concentration; solving higher-dimensional boundary value problems; analyzing higher-order moments of the stochastic concentration.
5. Analyzing the synchronization of stochastic hybrid oscillators driven by a common environmental switching process; extending the theory to take into account a partial dependence of the switching process on the continuous dynamics of each oscillator.
6. Modeling synaptically coupled neural networks as a stochastic hybrid system, where the individual spikes of a neural population are treated as the discrete process, and the synaptic currents driving the neurons to fire correspond to the continuous process. So far, stochastic hybrid neural networks are phenomenologically based [11, 24]. Can such networks be derived from a more fundamental microscopic theory, and is there a way of distinguishing the output activity of hybrid networks from those driven, for example, by Gaussian noise?
